# Mechanoreceptor plexin D1 regulates lymphatic valve morphogenesis and lymphedema pathogenesis

**DOI:** 10.1172/JCI193385

**Published:** 2026-07-01

**Authors:** Kar-Lai Pang, Vedanta Mehta, Claire Aitken, Sara E. Dobbins, Jing Yu, Gabriele Bonetti, Adam N. Keen, Feiran Zhang, Amélie Sabine, Tatiana V. Petrova, Paul R. Riley, E. Yvonne Jones, Sandro Michelini, Matteo Bertelli, John S. Reader, Pia Ostergaard, Ellie Tzima

**Affiliations:** 1 Division of Cardiovascular Medicine, Radcliffe Department of Medicine, and; 2 Centre for Human Genetics, University of Oxford, Oxford, United Kingdom.; 3 School of Health and Medical Sciences, City St George’s University of London, London, United Kingdom.; 4 Division of Structural Biology, Centre for Human Genetics, University of Oxford, Oxford, United Kingdom.; 5 MAGI Euregio, Bolzano, Italy.; 6 Department of Oncology, University of Lausanne and Ludwig Institute for Cancer Research, Lausanne, Switzerland.; 7 Department of Physiology, Anatomy and Genetics, University of Oxford, Oxford, United Kingdom.; 8 Diagnostic and rehabilitative Service, San Giovanni Battista Hospital, Rome, Italy.

**Keywords:** Cell biology, Vascular biology, Endothelial cells, Lymph

## Abstract

Lymphatic valves are essential for maintaining tissue fluid homeostasis, and their dysfunction leads to lymphedema, a morbid and disfiguring disease without a cure. Mechanical forces due to lymph flow are required for proper lymphatic valve development, yet it remains unclear how lymphatic endothelial cells (LECs) sense and decode mechanical signals. In this study, we identify the cell guidance semaphorin receptor plexin D1 (PLXND1) as a lymphatic mechanosensor required for lymphatic valve morphogenesis. Conditional genetic ablation of *Plxnd1* in LECs caused major defects in lymphatic valve development in 2 different lymphatic vascular beds. Mechanistically, PLXND1 acted as a mechanosensor within a lymphatic mechanocomplex, initiating distinct mechanical signals and activating the lymphatic valve transcriptional program through an unconventional pathway. Screening of patients with primary lymphedema identified *PLXND1* missense variants, and functional analysis established 2 pathogenic variants that selectively disrupt the ligand versus mechanosensing functions of this receptor. Variants associated with lymphedema in members of the mechanocomplex disrupted its formation, underscoring the central role of this complex in lymphatic valve biology. Our work uncovers a mechanosensing mechanism guiding lymphatic valve development, and has profound implications for the understanding and treatment of primary lymphedema in humans.

## Abstract 2

Plexin D1 regulates lymphatic valve morphogenesis and lymphedema

## Graphical abstract

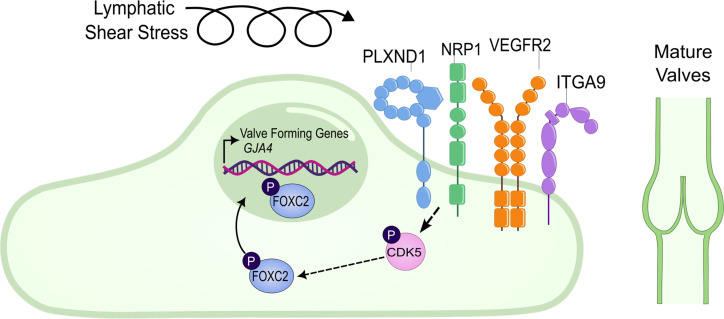

## Introduction

Lymphatic vessels transport interstitial fluid to the blood circulation and are central to tissue fluid homeostasis and additional recognized roles in lipid metabolism, inflammation, and immunosurveillance (1, 2). The flow of lymph against gravity is made possible by lymphatic pumping and the presence of specialized and regularly spaced bileaflet lymphatic valves that prevent lymph backflow (3, 4). Lymphatic valve malfunction, when genetic in nature, is associated with primary lymphedema, a morbid and disfiguring disease with no cure (5–7).

In concert with biochemical signals, biomechanical cues have emerged as critical regulators of lymphatics (3, 4). Lymph flow exerts a mechanical frictional force (shear stress) on the lymphatic endothelial cells (LECs) that line the valves that is low and oscillatory due to the forward and retrograde fluid movements (8–10). Notably, lymphatic vessels respond to lymph flow by growing valves, and exposure of LECs to oscillatory shear stress in vitro turns on transcription of key valve genes (10). Conversely, without lymph flow, valves do not grow in the developing embryo (11). There is therefore a reciprocal relationship between lymph flow and lymphatic valve formation, but the underpinning molecular mechanisms are incompletely understood.

Primary lymphedema is associated with variants in over 20 genes; however, the overwhelming majority of patients remain without a genetic diagnosis (6, 12). Furthermore, the primary lymphedema-associated genes identified so far code primarily for either cytoplasmic or transcription factors, therefore leaving unanswered the key question of how LECs sense and decode the mechanical stimulus of lymph flow to activate mechanotransduction pathways. Insights gained from studies of mechanotransduction in blood ECs have informed subsequent work in lymphatics (13–16), however, there is limited information on the lymphatic mechanosensors that detect and transduce the mechanical cues driving lymphatic valve development. We previously discovered that the cell guidance receptor plexin D1 (PLXND1), which typically operates via binding to its semaphorin ligands, has an additional function in mechanotransduction by sensing shear stress in arterial ECs and thus regulating the focal distribution of atherosclerosis (17). In this study, we identify a mechanosensitive pathway by which LECs turn physical stimuli into functional lymphatic valves. We demonstrate that PLXND1 is a lymphatic mechanosensor, forming a lymphatic mechanocomplex that instructs the development of lymphatic valves via an unconventional pathway. We have identified variants in *PLXND1* in patients with primary lymphedema and demonstrate that these variants selectively disrupted the ligand versus mechanosensing functions of this receptor. Thus, decoding of mechanical cues by PLXND1 is essential for lymphatic valve morphogenesis, and variants that interfere with either its mechanical or ligand activation are associated with primary lymphedema.

## Results

### Cell-autonomous Plxnd1 signaling controls lymphatic valve morphogenesis.

**Figure 1:**
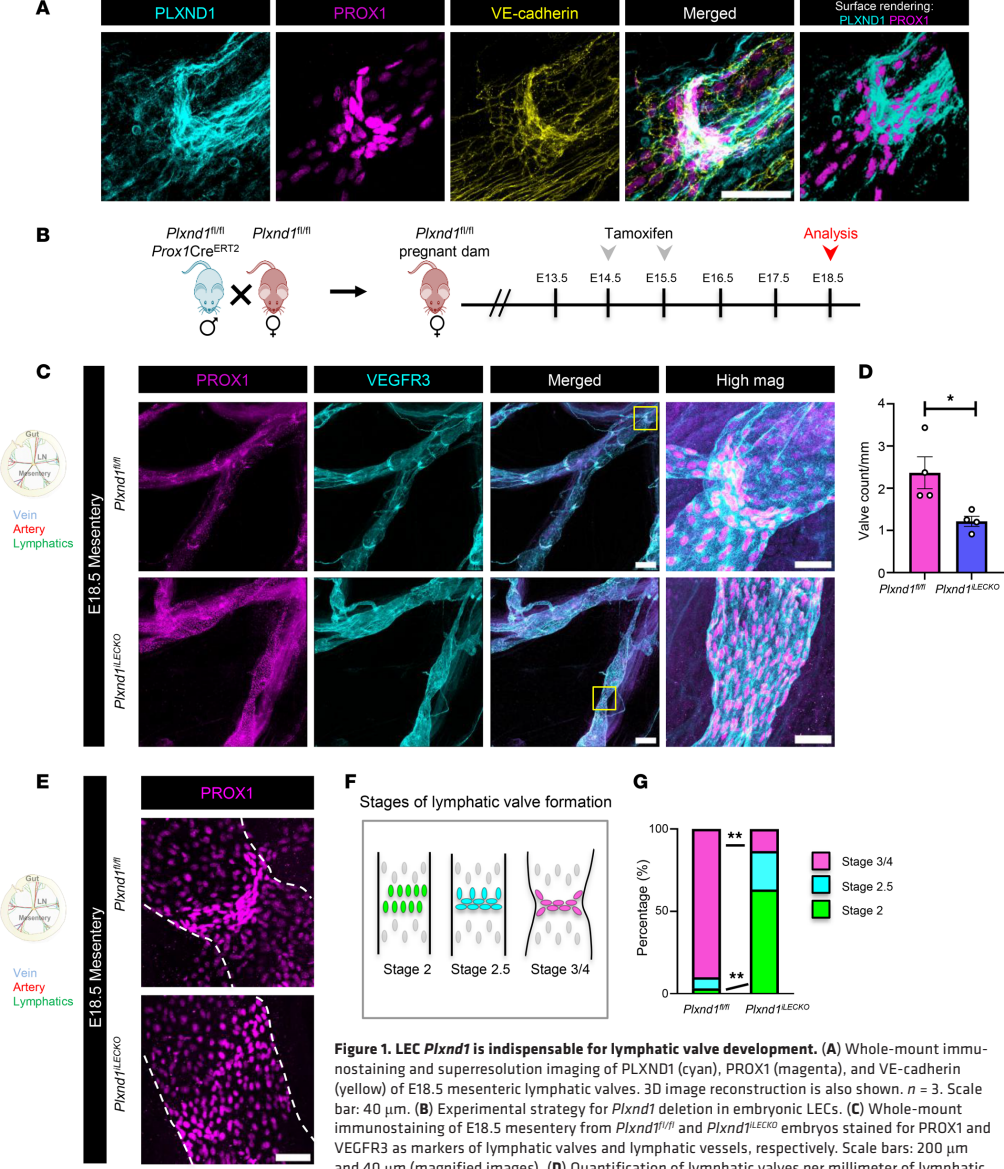
LEC *Plxnd1* is indispensable for lymphatic valve development. (**A**) Whole-mount immunostaining and superresolution imaging of PLXND1 (cyan), PROX1 (magenta), and VE-cadherin (yellow) of E18.5 mesenteric lymphatic valves. 3D image reconstruction is also shown. *n* = 3. Scale bar: 40 μm. (**B**) Experimental strategy for *Plxnd1* deletion in embryonic LECs. (**C**) Whole-mount immunostaining of E18.5 mesentery from *Plxnd1^fl/fl^* and *Plxnd1^iLECKO^* embryos stained for PROX1 and VEGFR3 as markers of lymphatic valves and lymphatic vessels, respectively. Scale bars: 200 μm and 40 μm (magnified images). (**D**) Quantification of lymphatic valves per millimeter of lymphatic collecting vessel in *Plxnd1^fl/fl^* and *Plxnd1^iLECKO^* E18.5 mesenteries (*n* = 4, data are the mean ± SEM; 2-tailed Student’s *t* test, *P < 0.05). (**E**) Representative images of E18.5 mesenteric lymphatic valves stained for PROX1 in *Plxnd1^fl/fl^* and *Plxnd1^iLECKO^* embryos. Scale bar: 50 μm. (**F**) Schematic diagram showing different stages of lymphatic valve formation. (**G**) Stage distribution within VFRs in E18.5 mesenteries. (*n* = 3; 10 VFRs from each embryo were analyzed; 2-tailed Student’s *t* test, **P < 0.01).

Single-cell transcriptomics data (18) show expression of *Plxnd1* in LECs, particularly valve LECs (Supplemental Figure 1, A and B; supplemental material available online with this article; https://doi.org/10.1172/JCI193385DS1). We confirmed these data by whole-mount immunostaining, superresolution microscopy, and 3D image reconstruction of mesenteric lymphatic valves at E18.5, which showed that PLXND1 protein was prominently expressed in valve LECs and colocalized with the lymphatic transcription factor PROX1 and the junctional adhesion molecule VE-cadherin (Figure 1A). To study the role of *Plxnd1* in the lymphatic vasculature, we crossed *Plxnd1^fl/fl^* mice with the tamoxifen-inducible lymphatic endothelial- cell specific *CreER^T2^* (*Prox1*) mice to generate *Plxnd1^fl/fl^* controls and *Plxnd1^fl/fl^* *Prox1^Cre^ER^T2^* mice (referred to hereafter as *Plxnd1^iLECKO^*) (Figure 1B). To ensure that there were no effects on embryonic development due to maternal *Plxnd1* deletion, we administered tamoxifen to control *Plxnd1^fl/fl^* pregnant dams (that had mated with *Plxnd1^fl/fl^* *Prox1^Cre^ER^T2^* males and therefore had a mix of *Plxnd1^fl/fl^* control embryos and *Plxnd1^iLECKO^* embryos). We chose to induce recombination (and therefore deletion of *Plxnd1* in LECs) at E14.5, when lymph flow starts and after lymphatic vessels have formed, as we wanted to decouple possible roles of *Plxnd1* in lymph flow–specific events from roles in early LEC specification (10). We observed specific deletion of *Plxnd1* in E18.5 LECs within lymphatic, but not blood vessels, thereby validating *Cre* efficiency and specificity (Supplemental Figure 1, C and D). Whole-mount staining of the mesentery showed regularly spaced lymphatic valves in control embryos, with characteristic V-shape morphology at high magnification, as shown by PROX1 and VEGFR3 staining (Figure 1, C and D) and integrin α 9 (ITGA9) (Supplemental Figure 2). In contrast, *Plxnd1^iLECKO^* embryos had markedly fewer valves made up of PROX1^+^ cells that failed to remodel into defined lymphatic valves (Figure 1, C and D). We also studied lymphatic valve formation in the embryonic dorsal skin, which also revealed defective and reduced numbers of lymphatic valves with loss of *Plxnd1* (Supplemental Figure 3, A and B). Next, we determined the developmental stage at which the lymphatic valve defect appears in *Plxnd1^iLECKO^* embryos, using PROX1^hi^-expressing cells as a marker for prospective valve leaflet cells (13). In stage 1, a few PROX1^hi^ LECs appeared in the vessel; by stage 2, their number increased and they formed a circumferential ring. As development proceeds, these cells reoriented perpendicularly to the vessel axis (stage 2.5) and migrated toward the lumen, forming disk-like protrusions and a local constriction (stage 3). The valves then elongated to form V-shaped leaflets with a dense extracellular matrix core (stage 4, Supplemental Figure 2). Examination of valve-forming regions (VFRs) in E18.5 mesenteries revealed a shift toward stage 2 and reduced stages 3–4 in *Plxnd1*^iLECKO^ embryos compared with *Plxnd1^fl/fl^* embryos (Figure 1, E–G). In control embryos, PROX^hi^ nuclei and VE-cadherin were detected at both the vessel wall and centrally, consistent with collective cell migration and valve protrusion (13, 19, 20) (Supplemental Figure 4). In contrast, *Plxnd1^iLECKO^* embryos showed PROX1^hi^ nuclei and VE-cadherin restricted to the vessel wall, indicating defective cell migration (Supplemental Figure 4). Analysis of lymphatic vascular patterning in the skin showed that the lymphatic vessels of *Plxnd1^iLECKO^* embryos were similar to those of *Plxnd1^fl/fl^* controls with regard to vessel width, branch points, and surface area in both the mesentery and embryonic dorsal skin (Supplemental Figure 5, A and B, and Supplemental Figure 6, A–D). These data therefore show that loss of *Plxnd1* in LECs, at a time when lymph flow starts, led to specific defects in lymphatic valve development. To understand whether PLXND1 is also required for valve maintenance, we deleted *Plxnd1* postnatally using tamoxifen-inducible, endothelial-cell specific *CreER^T2^* mice (*Plxnd1^iECKO^*). Similar to the findings in *Cdk5*-KO pups using endothelial-cell specific *CreER^T2^* mice (21), lymphatic vessels and valves appeared to be normal, and chylothorax was not observed in the *Plxnd1^iECKO^* pups (Supplemental Figure 7, A and B). Furthermore, we observed normal smooth coverage in the *Plxnd1^iECKO^* pups compared with control littermates (Supplemental Figure 8, A and B).

### PLXND1 regulates the lymphatic valve program via an unconventional pathway.

**Figure 2:**
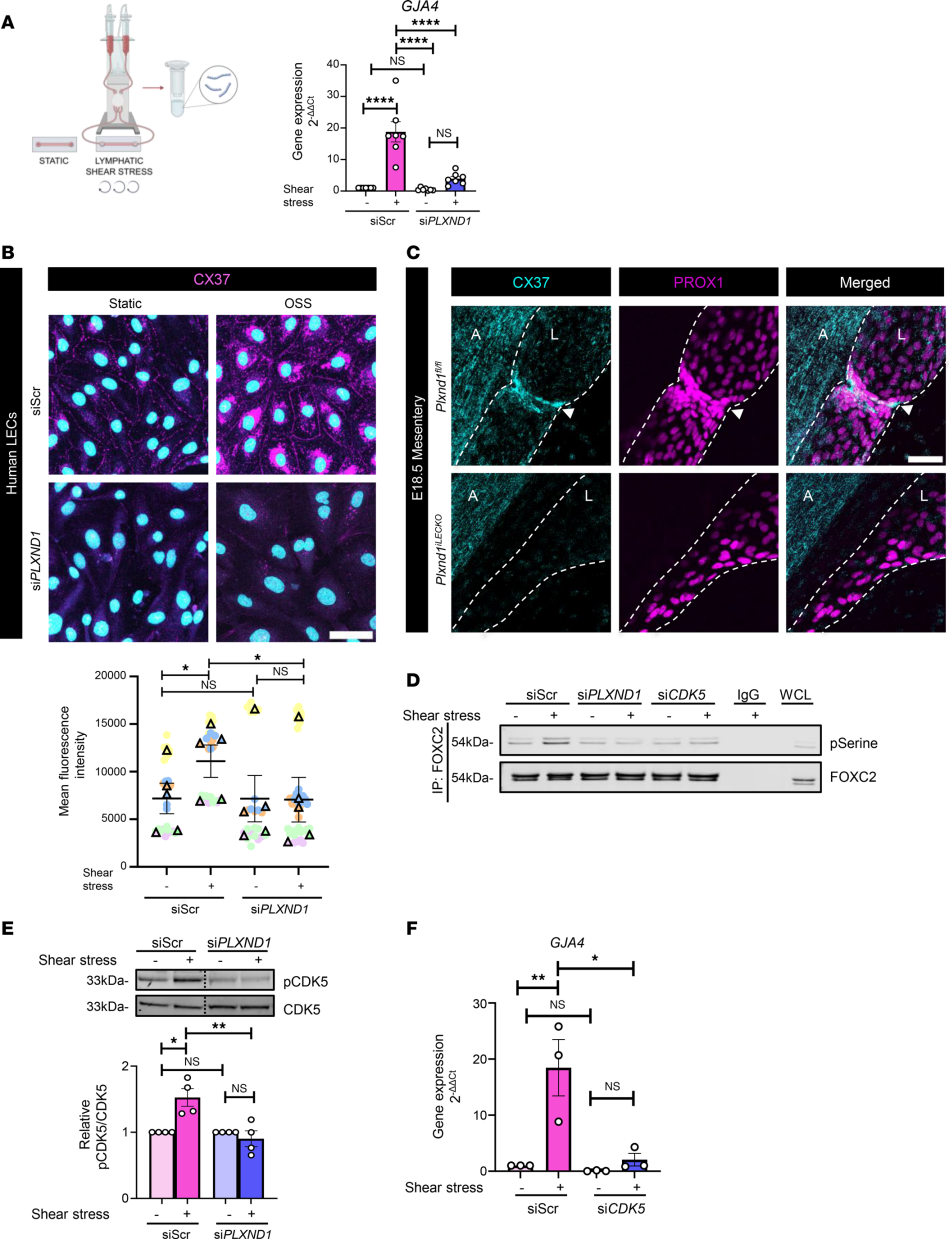
PLXND1 regulates the lymphatic valve program via an unconventional pathway. (**A**) Schematic of the lymphatic shear stress profile. Human LECs (HLECs) transfected with either scrambled or *PLXND1* siRNAs were left static or exposed to shear stress for 48 hours, RNA was isolated, and quantitative PCR (qPCR) was performed to quantify the expression of *GJA4* (*n* = 7, data are the mean ± SEM; 2-way ANOVA with Tukey’s multiple-comparison test, ****P < 0.0001). (**B**) HLECs transfected with scrambled or *PLXND1* siRNAs were left static or exposed to shear stress for 48 hours. Cells were fixed and immunostained to quantify the expression of CX37 (300–350 cells were analyzed per condition; *n* = 5 biological replicates; 2-way ANOVA with Tukey’s multiple-comparison test, *P < 0.05). Scale bar: 50 μm. (**C**) Immunofluorescence staining of *Plxnd1^fl/fl^* and *Plxnd1^iLECKO^* E18.5 mesentery for CX37 (cyan) and PROX1 (magenta). A, artery, L, lymphatic vessel. Arrowheads indicate lymphatic valves (*n* = 4, scale bar: 40 μm). (**D**) HLECs transfected with either scrambled, *PLXND1,* or *CDK5* siRNAs were left static or exposed to shear stress before immunoprecipitating FOXC2 and analyzing serine phosphorylation (*n* = 3). Immunoprecipitation with nonspecific IgG was used as a control. WCL, whole-cell lysate. (**E**) Phosphorylation of CDK5 (pY15) in HLECs transfected with scrambled or *PLXND1* siRNAs and exposed to shear stress (*n* = 4, data are the mean ± SEM; 2-way ANOVA with Tukey’s multiple-comparison test, *P < 0.05, **P < 0.01). Dashed line indicates where the blot was spliced. (**F**) HLECs transfected with either scrambled or *CDK5* siRNAs were left static or exposed to shear stress for 48 hours, and qPCR was performed to quantify the expression of *GJA4* (*n* = 3, data are the mean ± SEM; 2-way ANOVA with Tukey’s multiple-comparison test, *P < 0.05, **P < 0.01).

Mechanotransduction due to lymph flow is a critical regulator of the lymphatic valve transcriptional program (3, 4). To determine if the lymphatic valve defects observed with loss of lymphatic *Plxnd1* are associated with altered mechanotransduction, we assayed the expression of key lymphatic regulators in human LECs (transfected with either scrambled or *PLXND1* siRNAs) (Supplemental Figure 9A) in response to lymphatic valve shear stress (oscillatory shear stress [OSS]; ± 4 dynes/cm^2^, ¼ Hz) (10, 22). Our results showed that loss of *PLXND1* did not affect the mRNA levels (Supplemental Figure 9, B–D), protein levels, or nuclear localization (Supplemental Figure 10, A–C) of the key lymphatic transcriptional regulators *GATA2*, *PROX1*, or *FOXC2*; however, expression of the downstream target gene *GJA4* (which codes for CX37) was dramatically reduced in *PLXND1*-depleted LECs in response to lymphatic valve shear stress in vitro (Figure 2A). Notably, we confirmed reduced CX37 expression at the protein level in vitro (Figure 2B) and in vivo in lymphatic valves, but not arteries, of *Plxnd1^iLECKO^* mice (Figure 2C).

To explore the molecular mechanisms by which PLXND1 regulates mechanosensitive *GJA4* expression, we assayed serine phosphorylation of FOXC2, as it is critical for the transcriptional activation of this protein (10, 23). The results showed that shear stress increased serine phosphorylation of FOXC2 in control LECs, but this was abrogated with loss of *PLXND1* (Figure 2D). FOXC2 protein levels remained unchanged upon *PLXND1* deletion in vitro (Supplemental Figure 10C) and in vivo (Supplemental Figure 11, A and B). To explore this further, we investigated the kinase responsible for FOXC2 serine phosphorylation in response to lymphatic valve shear stress. We examined the role of CDK5, whose loss in LECs (21) phenocopies the *Plxnd1^iLECKO^* mice, by assaying its phosphorylation on Tyr15; our results showed that PLXND1 was required for activation of CDK5 in response to lymphatic valve shear stress (Figure 2E). In agreement with a role for CDK5 in this pathway, we found that CDK5 itself was essential for flow-induced serine phosphorylation of FOXC2 (Figure 2D) and upregulation of *GJA4* at both the mRNA level and CX37 at the protein level (Figure 2F and Supplemental Figure 12, A and B) (21). These results, therefore, demonstrate that PLXND1 regulates the lymphatic valve gene program via an unconventional mechanism that involves posttranslational modification of FOXC2 via CDK5-dependent phosphorylation and downstream CX37 upregulation.

### PLXND1 is a mechanosensor in LECs.

**Figure 3:**
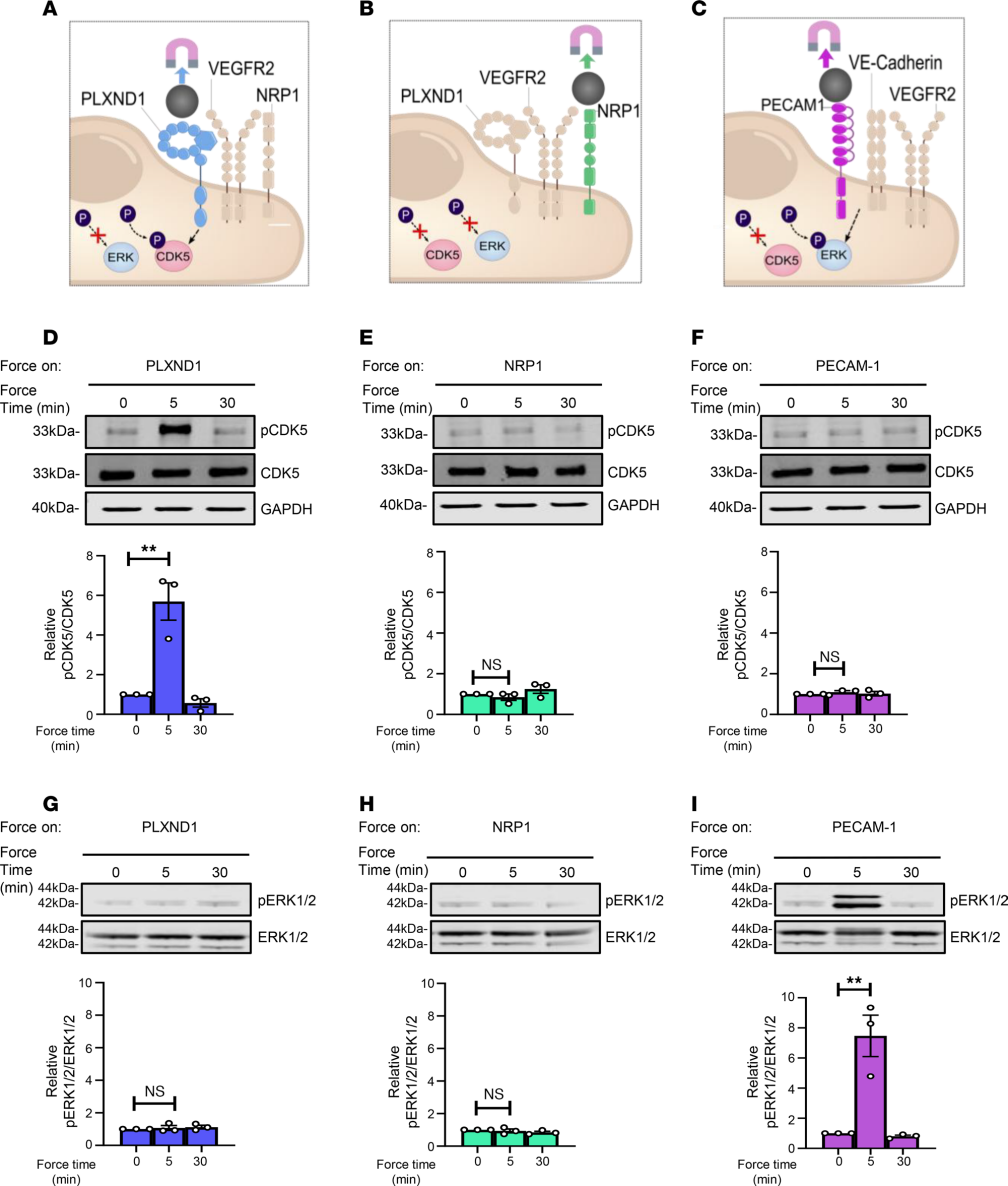
PLXND1 is a lymphatic force sensor. Schematic showing the tensional force experimental strategy using magnetic beads coated with antibodies against the extracellular domain of PLXND1 (**A**), NRP1 (**B**), or PECAM1 (**C**) in HLECs. HLECs were incubated with anti-PLXND1 (**D** and **G**), anti-NRP1 (**E** and **H**), or anti–PECAM-1 (**F** and **I**) antibody magnetic beads and subjected to 10 pN force for the indicated durations. Phosphorylation of CDK5 (pY15) and ERK1/2 (pT202/Y204) were determined by Western blotting, and quantification of the Western blots is shown (*n* = 3, data are the mean ± SEM; 1-way ANOVA with Kruskal-Wallis test, **P < 0.01).

The requirement for PLXND1 in LEC mechanotransduction raised the question of whether PLXND1 functions as a mechanosensor in these cells. To address this question, we applied tensional force on endogenous PLXND1 using magnetic beads coated with an antibody that recognizes the PLXND1 ectodomain (17). Tensional force on PLXND1 in LECs induced phosphorylation of CDK5, the kinase responsible for phosphorylation (and activation) of FOXC2 (Figure 3, A and D). This mechanosensing ability was specific to PLXND1, as force application on its coreceptor, NRP1, did not elicit a response (Figure 3, B and E). Crucially, the force-induced activation of the CDK5 pathway was unique to PLXND1, as force on another mechanosensor also expressed in LECs, PECAM-1, did not induce CDK5 phosphorylation (Figure 3, C and F). Instead, force on PECAM-1 induced activation of ERK1/2 (Figure 3I), whereas force on PLXND1 or NRP1 did not (Figure 3, G and H). These data therefore identify PLXND1 as a mechanosensor in LECs that is uniquely capable of activating the CDK5/FOXC2/CX37 pathway in response to mechanical force.

### A lymphatic mechanocomplex induced by lymph flow.

**Figure 4:**
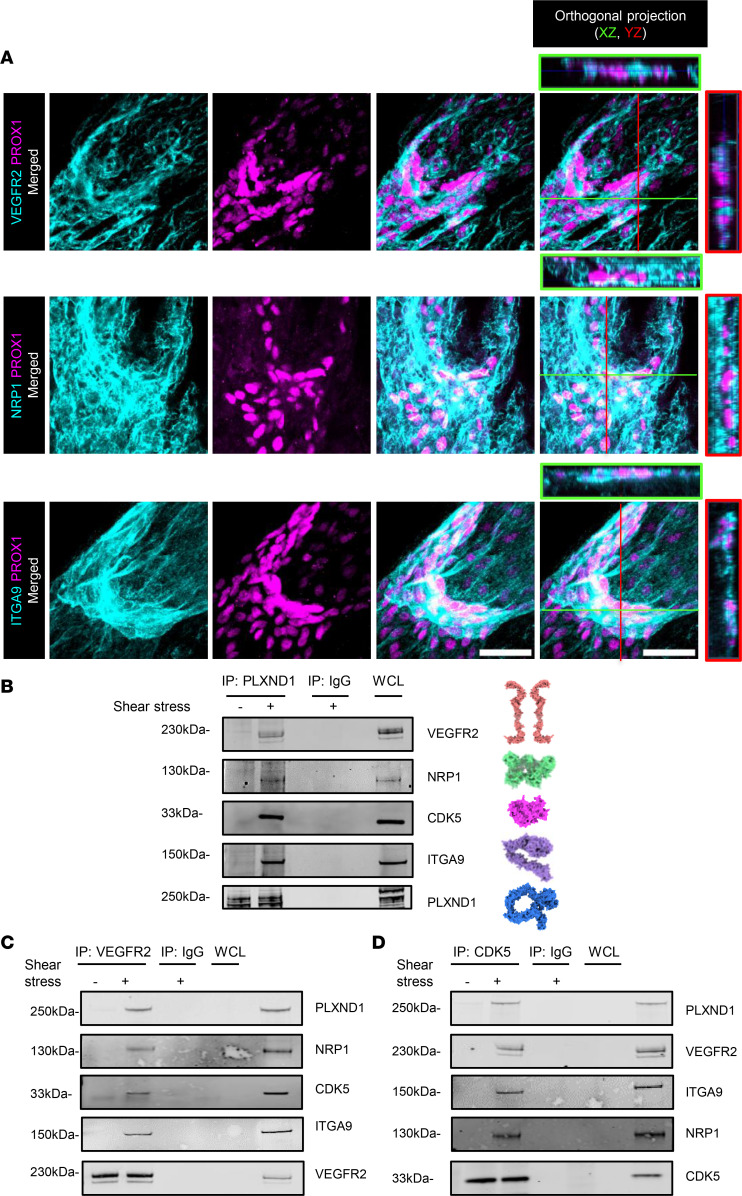
Force induces the formation of a lymphatic mechanocomplex. (**A**) Whole-mount immunostaining and superresolution imaging of E18.5 mesentery lymphatic valves for VEGFR2, NRP1, and ITGA9 and staining for the lymphatic valve marker PROX1 (*n* = 3. Scale bar: 40 μm). Orthogonal views in 2 planes are also shown. (**B**) HLECs were left static or exposed to shear stress before immunoprecipitating PLXND1 and analyzing for the presence of VEGFR2, NRP1, CDK5, and ITGA9 (*n* = 2). Immunoprecipitation with nonspecific IgG was used as a control. HLECs were left as static or exposed to shear stress before immunoprecipitating VEGFR2 (**C**) or CDK5 (**D**) and analyzed for its association with PLXND1, NRP1, CDK5 and ITGA9, or PLXND1, VEGFR2, ITGA9 and NRP1, respectively. Immunoprecipitation with nonspecific IgG was used as a control. *n* = 2 (**C** and **D**).

An emerging paradigm in mechanosensation is that mechanosensors form complexes with auxiliary receptors (24); however, to our knowledge, no mechanically induced complex has ever been reported in lymphatics. In considering possible interactors of PLXND1, we first assessed lymphatic valves in vivo and human LECs in vitro for the expression of known PLXND1 coreceptors. Consistent with previous reports, we found that coreceptors of PLXND1, including VEGFR2 (25) and NRP1 (26) (Figure 4A), NRP2 (27), and VEGFR3 (28) (Supplemental Figure 13) were also expressed in lymphatic valves, although expression of NRP2 was much lower in the valve compared with the lymphangion (Supplemental Figure 13) (10). We found that ITGA9, a signature integrin of LECs required for lymphatic valve formation (29), was also highly expressed (Figure 4A). Human LECs express VEGFR2, NRP1, NRP2, and VEGFR3 (at varying levels compared with human aortic ECs); expression of ITGA9 was exclusive to LECs (Supplemental Figure 14). To determine whether PLXND1 forms mechanical force–induced interactions in LECs, PLXND1 immunoprecipitates from static and sheared LECs were immunoprobed for candidate coreceptors. Surprisingly, in response to lymphatic valve shear stress, PLXND1 formed a mechanocomplex with NRP1 and VEGFR2 (Figure 4B), but not with NRP2 and VEGFR3 (Supplemental Figure 15), despite the well-recognized roles of NRP2 and VEGFR3 in LECs (27, 30, 31). The kinase CDK5 and ITGA9 were also part of the mechanocomplex (Figure 4B). These interactions were not observed in static cells or when a control IgG antibody was used for immunoprecipitations. To solidify the existence of this lymphatic mechanocomplex, we also performed reverse immunoprecipitations of both VEGFR2 and CDK5 and saw the same flow-dependent complex formation (Figure 4, C and D). Knockdown of the PLXND1 coreceptors VEGFR2 or NRP1 abrogated the formation of the flow-dependent mechanocomplex (Supplemental Figure 16, A and B). Notably, SEMA3E treatment did not induce formation of the mechanocomplex (Supplemental Figure 17). These results therefore demonstrate the discovery of a PLXND1-based lymphatic mechanocomplex that formed in response to lymphatic valve shear stress.

### Rare variants in the PLXND1 gene in patients with lymphedema.

**Figure 5:**
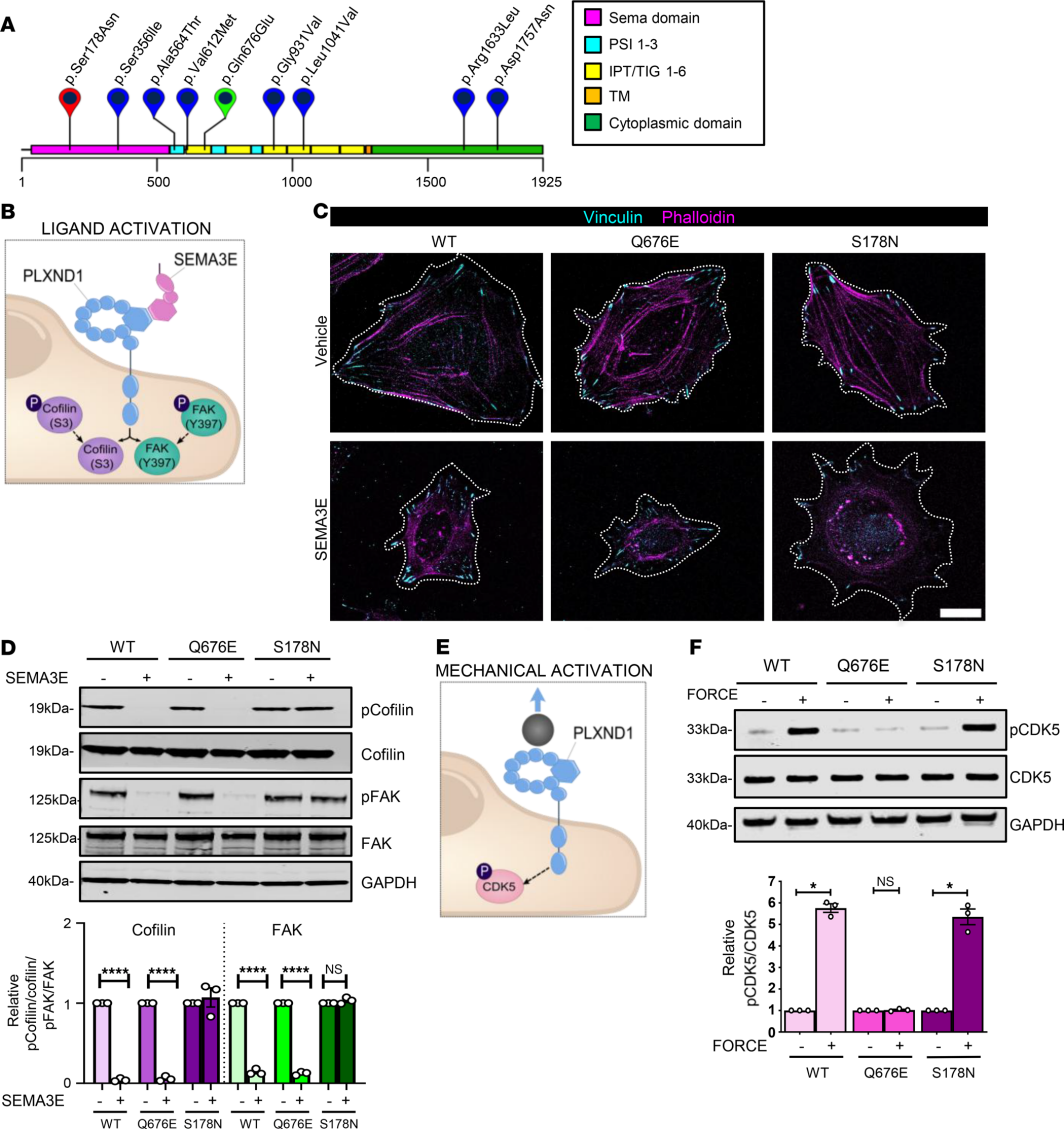
*PLXND1* variants associated with lymphedema selectively disrupt ligand versus mechanical activation of the receptor. (**A**) Lollipop plot of *PLXND1* variants showing a 2D representation of their location in the PLXND1 protein. The red variant (S178N) resides within the SEMA binding domain, the green variant (Q676E) is located distal from this domain, and the blue variants correspond to mutations that have not yet been functionally characterized. PSI, plexin-semaphorin-integrin homology domains; IPT/TIG, immunoglobulin plexin transcription/transcription factor immunoglobulin domains; TM, transmembrane domain. (**B**) Schematic showing ligand activation of PLXND1 via SEMA3E and the downstream signaling investigated. (**C**) SEMA3E-induced cell collapse assays. HLECs in which endogenous *PLXND1* was knocked down were transduced with adenoviruses expressing WT or mutant Q676E or S178N PLXND1 and were subsequently treated with SEMA3E and immunostained with anti-vinculin antibody (cyan) and phalloidin (magenta). Representative images from 3 biological replicates are shown. Scale bar: 20 μm. (**D**) HLECs in which endogenous *PLXND1* was knocked down were transduced with adenoviruses expressing WT or mutant PLXND1 and treated with SEMA3E before assaying phosphorylation of FAK (pY397) and cofilin (pS3) (*n* = 3, data are the ± SEM; 2-way ANOVA with Tukey’s multiple-comparison test, ****P < 0.0001). (**E**) Schematic showing mechanical activation of PLXND1 via tensional force application using magnetic beads and the downstream signaling investigated. (**F**) HLECs in which endogenous *PLXND1* was knocked down were transduced with adenoviruses expressing WT or mutant PLXND1 and incubated with anti-PLXND1 magnetic beads followed by force application. Phosphorylation of CDK5 (pY15) was determined (*n* = 3, data are the mean ± SEM; 2-way ANOVA with Tukey’s multiple-comparison test, *P < 0.05).

Patients with primary lymphedema often present with variants in genes important for lymphatic valve formation (2, 6). We hypothesized that since *Plxnd1* regulates lymphatic valve formation in mice, we might identify variants in this gene in humans with lymphatic disease. We examined exome sequencing data from 235 individuals from hospitals in Italy; crucially, these patients tested negative for variants in known lymphedema and lymphatic malformation–causing genes. We identified potential candidate variants in the *PLXND1* gene in 2 patients with primary lymphedema (Supplemental Table 4, variants 1 and 5, which was confirmed by Sanger sequencing (Supplemental Figure 18, A and B). Patient RX626.2021 was diagnosed with bilateral (right>left) lower limb lymphedema at 59 years of age, while patient RX528.2021 was diagnosed with bilateral (right>left) toe and dorsal foot edema at birth (Supplemental Figure 18). Both patients tested positive on the Stemmer sign (toe skin pinch and lift) test and showed more edema in the orthostatic (standing) position than in the clinostatic (lying down) position; clinical data and lymphoscintigraphy results are shown in Supplemental Figure 18, C–E. In silico analysis revealed that the c.533G>A; pSer178Asn variant is located within the semaphorin (SEMA) binding domain of PLXND1, and the c.2026C>G; p.Gln676Glu variant is distal from its SEMA domain (Figure 5A). The 2 variants were chosen for further investigation and functional validation.

To further determine the relevance of this finding, we examined exome sequencing data from 272 patients with primary lymphedema from St George’s University Hospitals NHS Foundation Trust, London, in conjunction with whole-genome data from 163 additional patients recruited for the 100,000 Genomes Project. An additional 7 potential candidate variants in the *PLXND1* gene were identified (Figure 5A and Supplemental Table 4), which supports a potential link between *PLXND1* variants and susceptibility to lymphedema.

### Function of lymphedema variants in ligand and mechanical activation of PLXND1.

To investigate the functional significance of the putative c.533G>A; pSer178Asn and c.2026C>G; p.Gln676Glu lymphedema variants, we transduced human LECs (depleted of endogenous *PLXND1* via siRNAs) with adenoviruses expressing either WT PLXND1 or the PLXND1 Ser178Asn (S178N) or Gln676Glu (Q676E) mutants. Immunoblotting revealed similar expression levels of WT and mutant PLXND1 in LECs (Supplemental Figure 19A). To examine possible effects of the mutations on the subcellular localization of PLXND1, we performed confocal imaging of transduced Cos7 cells. Both WT and mutant PLXND1 proteins showed similar subcellular localization at the plasma membrane in Cos7 cells (Supplemental Figure 19B), suggesting that the mutations do not affect the expression or subcellular localization of PLXND1. We therefore carried out assays that focused on assessing the effect of the mutations on both the canonical, semaphorin-binding role of PLXND1 and its newly identified mechanical role in lymphatics.

To assay possible effects of the mutations on the canonical ligand activation of PLXND1, we examined responses to semaphorin 3E (SEMA3E) (Figure 5B). LECs (depleted of endogenous *PLXND1* via siRNAs) transduced with adenovirus expressing WT PLXND1 displayed the typical cell collapse/retraction in response to ligand activation of PLXND1, also observed in other cell types (32–34) (Figure 5C); LECs expressing mutant Q676E PLXND1 also collapsed in response to SEMA3E, suggesting that the mutation does not interfere with the ligand activation of the receptor. In contrast, LECs expressing the S178N PLXND1 mutant failed to collapse, thus showing that this mutation interferes with the normal ligand activation response. To further explore the role of the mutations in PLXND1 ligand activation, we established 2 additional assays to measure ligand activation of PLXND1: we reasoned that SEMA3E-induced cell collapse is preceded by changes in the activation of cytoskeletal proteins. The actin-severing protein cofilin, which is activated through dephosphorylation at Ser3, regulates RhoA-dependent actin stress fiber formation (35, 36), while the focal adhesion protein FAK (autophosphorylated at Tyr397) regulates focal adhesion growth (37, 38). In response to SEMA3E, both WT and Q676E PLXND1 mutant–expressing LECs showed similar changes to the phosphorylation of cofilin and FAK (Figure 5D), consistent with similar cytoskeleton remodeling, ultimately leading to cell collapse (Figure 5C). Strikingly, these responses were absent in the S178N PLXND1–expressing LECs. Collectively, these experiments showed that ligand activation of PLXND1 was specifically affected by the S178N mutation (located within the SEMA domain of PLXND1) but not the Q676E mutation, away from the SEMA domain.

To analyze the effects of the lymphedema variants on the mechanical activation of PLXND1, we used the tensional force system to apply mechanical force on PLXND1 (Figure 5E). LECs depleted of endogenous PLXND1 and transduced with adenoviruses expressing WT, S178N, or Q676E PLXND1 were subjected to force, and the phosphorylation of CDK5 was assayed by Western blotting using phosphorylated Y15 (pY15) CDK5–specific antibodies. Like endogenous PLXND1, the WT PLXND1 transduced in LECs was able to detect mechanical force and activate the CDK5 pathway, as evidenced by increased phosphorylation of CDK5 on Y15 (Figure 5F). The S178N mutant showed a similar response. In contrast, Q676E PLXND1–expressing LECs failed to activate CDK5 in response to force, indicating that this lymphedema variant suppressed the mechanical activation of PLXND1. Taken together, these results show that 1 lymphedema variant selectively affected the ligand activation of PLXND1, while the other variant selectively affected the mechanical activation of the receptor.

### Lymphedema variants interfere with lymphatic mechanocomplex formation.

**Figure 6:**
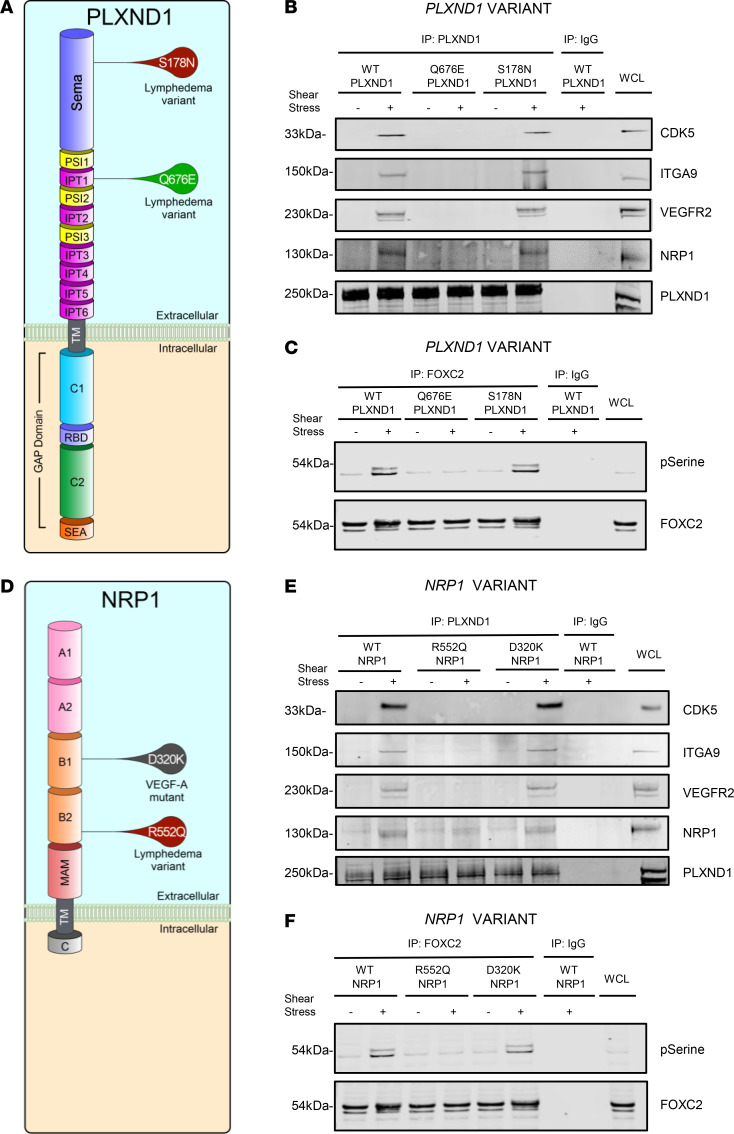
Lymphedema-associated variants disrupt lymphatic mechanocomplex formation and impair FOXC2 phosphorylation. (**A**) Schematic of the PLXND1 domain structure showing the positions of the lymphedema-associated S178N and Q676E mutations. (**B**) HLECs in which endogenous *PLXND1* was knocked down were transduced with adenoviruses expressing WT or mutant PLXND1 and subjected to shear stress before immunoprecipitating PLXND1 and analyzing for its association with CDK5, ITGA9, VEGFR2, and NRP1 (*n* = 3). (**C**) HLECs in which endogenous *PLXND1* was knocked down were transduced with adenoviruses expressing WT or mutant PLXND1 and exposed to shear stress before immunoprecipitating FOXC2 and analyzing for serine phosphorylation. (**D**) Schematic of the NRP1 domain structure showing position of the lymphedema-associated R552Q mutation and the VEGF-A D320K mutation. (**E**) HLECs in which endogenous *NRP1* was knocked down were transduced with adenoviruses expressing WT or mutant NRP1 were subjected to shear stress before immunoprecipitating PLXND1 and analyzing for its association with CDK5, ITGA9, VEGFR2, and NRP1 (*n* = 3). (**F**) HLECS in which endogenous *NRP1* was knocked down were transduced with adenoviruses expressing WT or mutant NRP1 and exposed to shear stress before immunoprecipitating FOXC2 and analyzing for serine phosphorylation.

Given that lymphatic valve shear stress induces formation of a PLXND1-NRP1-VEGFR2-ITGA9 lymphatic mechanocomplex and the Q676E PLXND1 variant (Figure 6A) disrupts the mechanical activation of PLXND1, we tested the hypothesis that this variant affects formation of the lymphatic mechanocomplex. We transduced LECs (depleted of endogenous *PLXND1*) with adenoviruses expressing either WT, Q676E, or S178N PLXND1 and subjected them to lymphatic shear stress before immunoprecipitating PLXND1. Western blotting of immunoprecipitates revealed that, while WT PLXND1 associated with NRP1, VEGFR2, and ITGA9 in response to shear stress, the Q676E PLXND1 mutant did not support formation of the mechanocomplex (Figure 6B). To further explore the role of *PLXND1* variants in the mechanosensitive CDK5/FOXC2/CX37 pathway, we examined 2 key downstream events: FOXC2 phosphorylation and *GJA4* expression. WT PLXND1 induced serine phosphorylation of FOXC2 (Figure 6C) and *GJA4* expression (Supplemental Figure 20, A and B), however, Q676E PLXND1 failed to support FOXC2 phosphorylation (Figure 6C) and upregulate *GJA4* expression (Supplemental Figure 20B). Neither FOXC2 phosphorylation nor *GJA4* expression was affected by S178N PLXND1 (Figure 6C and Supplemental Figure 20A). Thus, this mutation not only abolished the mechanical activation of the receptor (Figure 5F), but also disrupted formation of the lymphatic mechanocomplex. Crucially, the S178N mutant, required for the ligand response, did not interfere with the formation of the mechanocomplex.

In addition to *PLXND1*, variants in another member of the lymphatic mechanocomplex have also been associated with lymphatic diseases; a missense variant c.1655G>A, corresponding to R552Q in *NRP1* segregates with lymphedema (39), although its functional relevance and mechanism of action have not been investigated. To test if the *NRP1* lymphedema-associated variant affects the formation of the lymphatic mechanocomplex, we generated this variant in a human NRP1 adenovirus expression vector. We also generated an additional NRP1 mutation (D320K) (Figure 6D), which inhibits its ability to bind to VEGF-A but does not affect its expression (40), as we hypothesized that disruption of NRP1-VEGFA binding would not interfere with mechanical responses. We transduced LECs depleted of endogenous NRP1 with adenoviruses encoding WT, R552Q, or D320K NRP1 and analyzed expression levels of the various constructs by Western blotting. Their subcellular localization was assessed in Cos7 cells by confocal imaging. We found that both the lymphedema R552Q variant and the VEGF-A D320K NRP1 mutant were expressed at levels similar to those of WT NRP1 (Supplemental Figure 21A). Similarly, the surface expression was not affected by the mutations (Supplemental Figure 21B). We therefore tested the effects of the mutants on formation of the lymphatic mechanocomplex. LECs depleted of endogenous NRP1 and transduced with adenoviruses expressing WT, R552Q, or D320K NRP1 were subjected to lymphatic shear stress, and formation of the mechanocomplex was assessed following immunoprecipitation of PLXND1. Our data showed that LECs expressing WT NRP1 formed the mechanocomplex, however, the lymphedema missense variant R552Q led to a complete loss of the mechanocomplex. Interestingly, LECs expressing the D320K NRP1 mutant showed normal mechanocomplex formation in response to lymphatic shear stress, suggesting that the NRP1 mutation that interferes with VEGF-A binding does not affect the mechanical response (Figure 6E). We next examined how NRP1 mutants affect the mechanosensitive CDK5/FOXC2/CX37 cascade. WT NRP1 induced robust FOXC2 serine phosphorylation and *GJA4* expression, whereas the R552Q variant failed to induce FOXC2 phosphorylation (Figure 6F) or *GJA4* expression (Supplemental Figure 20C). PLXND1 expression level remained unchanged by the NRP1 mutation (Supplemental Figure 20D). Collectively, these results identify disruption of the lymphatic mechanocomplex as the shared biological mechanism underlying rare variants in *PLXND1* and *NRP1*.

## Discussion

Here, we report the discovery of a molecular mechanism that lymphatic vessels use to sense and decode mechanical signals that govern lymphatic valve development, providing evidence from cellular, murine, and human genetic studies. We show that the guidance receptor PLXND1 regulated mechanosensitive valve development via an unconventional CDK5/FOXC2/CX37 pathway that involves posttranslational modification of FOXC2 (Supplemental Figure 22). In response to lymph flow, PLXND1 formed a complex with NRP1, VEGFR2, ITGA9, and CDK5, which is central. Tensional force experiments demonstrated that PLXND1 is a lymphatic mechanosensor that is uniquely capable of mechanically activating the CDK5/FOXC2 pathway and downstream CX37 expression. In vivo, deletion of *Plxnd1* specifically in LECs resulted in reduced CX37 expression and defective lymphatic valve formation. Genetic studies revealed *PLXND1* variants underlying human lymphatic disease, and functional analysis identified 2 lymphedema *PLXND1* variants that selectively perturbed the ligand versus mechanical functions of this receptor.

A role for semaphorin/plexin signaling in lymphatics has been reported previously, although mechanisms focused exclusively on classical ligand-repulsive effects (26, 41, 42). PLXND1 is canonically activated by semaphorins, which in turn cause cell collapse. Our findings confirm that this activation also holds true in LECs but identify that PLXND1 can also sense and respond to mechanical force. Our studies demonstrate that LEC PLXND1 can be activated through 2 distinct mechanisms — ligand-dependent and mechanically induced — each essential for proper LEC function. Biochemical and genetic analyses revealed that these 2 modes of activation are mediated by separable molecular processes. Notably, we identified naturally occurring *PLXND1* variants that selectively disrupted each pathway: the Ser178Asn variant impaired ligand-mediated activation, whereas the Gln676Glu variant specifically interfered with mechanical responsiveness. Together, these findings establish PLXND1 as a dual-mode receptor that integrates biochemical and biomechanical cues to coordinate lymphatic valve morphogenesis. Disruption of either mode of activation led to valve malformation, highlighting the requirement for both ligand-dependent and mechanosensitive PLXND1 signaling in proper lymphatic valve development and function.

It is widely accepted that mechanotransduction due to lymph flow regulates lymphatic function, however, information on the molecular sensors responsible is scarce. The Piezo-type mechanosensitive ion channel component 1 (PIEZO1) plays a critical role in lymphatics (13, 14, 43), and variants in *PIEZO1* are associated with lymphatic diseases (44, 45). Cell-surface receptors, including PECAM-1, VE-cadherin, VEGFR2/3, and Notch 1, are essential for proper lymphatic development, partly via mechanotransduction mechanisms (15, 16, 46, 47), but direct mechanosensing roles for these receptors in lymphatics have not been demonstrated. Our work identifies a mechanosensor in lymphatics, PLXND1, and defines the mechanotransduction pathway via which it activates the lymphatic valve genetic program.

Our discovery that PLXND1 partners with ITGA9 suggests that, while PLXND1 prefers the same core partners (NRP1 and VEGFR2) as in arterial ECs (17), the lymphatic mechanocomplex matches the organ-specific microenvironment of lymphatics. The core components of the mechanocomplex are therefore universal but with angiotypic elements, such as ITGA9, which is specifically expressed in LECs but not blood ECs (29, 48) (Supplemental Figure 14). Crucially, ITGA9 is essential for lymphatic development (29) and is also a potential candidate in primary lymphedema (49); here, we reveal a role for ITGA9 as an auxiliary receptor, forming part of the PLXND1-NRP1-VEGFR2 core mechanocomplex. Crucially, lymphedema-associated variants in either *PLXND1* or *NRP1* disrupted formation of the mechanocomplex, thus raising the possibility that interference with this complex might represent a common mechanism by which deleterious mutations disrupt lymphatic function. More work is needed to determine whether the remaining 7 variants are pathogenic and the potential molecular mechanisms responsible. Cumulatively, our work reveals a mechanosensing mechanism that guides lymphatic valve development, with fundamental implications in primary lymphedema.

## Methods

### Sex as a biological variable.

Both male and female embryos were included across all experiments in this study.

### Experimental mice.

For specific deletion of *Plxnd1* from LECs, *Plxnd1^fl/fl^* mice were crossed with *Prox1^Cre^ER^T2^* mice (strain 022075 [ref. 50]). Mice lacking *Plxnd1* in LECs are designated as *Plxnd1^iLECKO^*. For specific deletion of *Plxnd1* from both BECs and LECs, *Plxnd1^fl/fl^* mice were crossed with *Cdh5-^Cre^ER^T2^* mice. Mice lacking *Plxnd1* in ECs are designated as *Plxnd1^iECKO^*. Adult *Plxnd1^fl/fl^* female mice were subjected to timed mating by crossing them with adult *Plxnd1^fl/fl^ Prox1^Cre^ER^T2^* male mice. Female mice were checked every morning for a copulation plug. The day of copulation plug detection was assigned as E0.5. For the induction of Cre-mediated recombination in embryos, pregnant mice were fed 2 mg tamoxifen dissolved in 100 μL corn oil via oral gavage at E14.5 and E15.5, and embryos were harvested at E18.5 (embryos were a mixture of *Plxnd1^fl/fl^* and *Plxnd1^fl/fl^Prox1^Cre^ER^T2^* and comprised the control and experimental groups comparison). For the induction of Cre-mediated recombination in pups, pups were injected with 50 μg tamoxifen dissolved in 20 μL corn oil via the intragastric route on P1, P2, and P3, and pups were sacrificed at P6 (pups were a mixture of *Plxnd1^fl/fl^* and *Plxnd1^fl/fl^ Cdh5-^Cre^ER^T2^*).

### Whole-mount immunostaining of mesenteries.

Mesenteries were dissected from embryos and pups and fixed in 4% PFA or 1% PFA for 2 hours to overnight (depending on the antibody) at 4°C. They were permeabilized in 0.5% Triton X-100 in PBS for 15 minutes at room temperature (RT) and blocked in 3% BSA/0.3% Triton X-100 or 5% donkey serum/0.5% BSA/0.1% Triton X-100 for 2 hours at RT. Immunostaining was performed by incubating primary antibodies at 1:200 in blocking buffer at 4°C overnight. Tissues were washed in 0.1% Triton X-100/PBS 3 times at 4°C for 1 hour each time and incubated with Alexa Fluor 405–, 488–, 568–, and 647–conjugated secondary antibodies at a 1:300 dilution for 2 hours in the dark at RT. After washing, mesenteries were removed from the intestine using microscissors by cutting in close proximity to the gut wall. The mesenteries were unfolded on the glass slide using forceps and mounted flat with ProLong Glass Antifade Mountant (Invitrogen, Thermo Fisher Scientific, P36980) (51–53). For a complete list of antibodies, see Supplemental Table 2.

### Whole-mount immunostaining of skin.

Immunostaining of whole-mount dermal skin from E18.5 embryos was performed using the iDISCO protocol as previously described (54, 55). In short, embryos were fixed in 1% PFA in a 6-well plate overnight at 4°C. After washing with ice-cold PBS, embryonic dermal skin was removed from the ears to just under the forelimbs and was pinned on the sylgard plate. Samples were washed twice in PBST (1× PBS with 0.2% Triton X-100) for 30 minutes each time and were incubated in PBST with 20% DMSO for 4 hours. Samples were then incubated in PTTDND (1× PBS with 0.1% Tween 20, 0.1% Triton X-100, 0.1% deoxycholate, 0.1% NP40, and 20% DMSO) overnight at 4°C. After washing the samples twice with PBST for 30 minutes each time, samples were incubated in PTDG (1× PBS with 0.2% Triton X-100, 20% DMSO, and 0.3 M glycine) for 4 hours and were then blocked in blocking buffer (1× PBS with 0.2% Triton X-100, 10% DMSO, and 6% donkey serum) overnight at 4°C. After washing twice with PTWH (1× PBS with 0.2% Tween 20 and 10 μg/mL heparin) for 30 minutes each time, samples were incubated with primary antibodies in PTWH with 5% DMSO and 3% donkey serum overnight at 4°C. Samples were then washed 4 times in PTWH for 30 minutes each time. The samples were then incubated with secondary antibodies in PTWH with 3% donkey serum overnight and then subjected to series of washing in PTWH and PBS and were finally mounted in ProlongGold (Invitrogen, Thermo Fisher Scientific). Skin was placed in adequate mounting media with the inner (dermal) side facing upward and the ears of the dorsal skin aligned toward to the anterior end of the slide. Skin samples were mounted with glass coverslips and sealed with nail varnish for visualization. For complete list of antibodies see Supplemental Table 2.

### Co-immunoprecipitation and Western blotting.

For co-immunoprecipitation studies, cells were lysed as previously described (56) and supplemented with protease (Roche; 11836170001) and phosphatase inhibitor cocktail tablets (PhosSTOP; 04906837001). Lysates were precleared by incubation with 10 μL protein A/G plus sepharose beads (Santa Cruz Biotechnology, sc-2003) for 1 hour at 4°C, before incubation with 20 μL protein A/G plus sepharose beads that had been precoupled with primary antibody for 2 hours at 4°C. After washing the beads 3 times, immunoprecipitation complexes were eluted by boiling in 2× SDS buffer for 5 minutes. Lysates were resolved on 4%–12% gradient gels and transferred onto nitrocellulose membranes, followed by incubation with Alexa Fluor 790– or Alexa Fluor 680–conjugated secondary antibodies. When phosphorylated and total forms of a protein were detected using antibodies raised in the same species, 2 identical gels were run and transferred simultaneously under the same conditions in the same tank. One membrane was probed with the phosphorylation-specific antibody and the other with the total protein antibody. Each nitrocellulose membrane was also probed with an anti-GAPDH antibody as a loading control. Images were acquired on a LI-COR Odyssey (LI-COR Biosciences) infrared scanner. For co-immunoprecipitation experiments, given the limited availability of lysates, some nitrocellulose membranes were sequentially probed with 2 antibodies that recognize proteins of different molecular weights. Densitometric quantification of bands was performed using the ImageStudio software (LI-COR Biosciences). For a complete list of antibodies, see Supplemental Table 3. The Quick Western Kit (926-69100, LI-COR Biosciences) was used for development of all post-immunoprecipitation samples. We noted that PLXND1 appears as a doublet when the Quick Western Kit was used in combination with the custom PLXND1 antibody.

### Statistics and quantification.

Statistical analyses were performed using GraphPad Prism 10 software (GraphPad Software). All experiments were performed at least 3 times independently. Statistical significance was tested by using unpaired Student’s *t* test between 2 groups. Data were tested for normality using the Shapiro-Wilk test, and homoscedasticity was tested using the Levene test. A 1-way ANOVA or 2-way ANOVA with Kruskal-Wallis test or Tukey’s multiple-comparison post hoc test was used to compare differences between multiple groups. Differences were considered statistically significant when the *P* value was less than 0.05. Data are presented as the mean ± SEM. All image analyses were performed by operators who were blinded to the treatments administered.

### Study approval.

All mouse experiments were conducted under the approval and authorization of both the University of Oxford Local Animal ethics and Welfare committee and by the Home Office in the UK. The project licenses used in this work are as follows: P9133D191 and PP8815542.

### Data availability.

Values for all data points in graphs are reported in the Supporting Data Values file. Additional data supporting the findings of this study are available from the corresponding author upon request. For further information, please see also Supplemental Methods.

## Author contributions

KLP performed and was involved in most of the experiments, analyzed data, and prepared figures. VM performed co-immunoprecipitation experiments, magnetic force application, and biochemical assays of the semaphorin challenge experiments, analyzed data, and prepared figures. VM supervised and VM and CA performed cloning of the NRP1 WT and mutant constructs and PLXND1 WT and mutant constructs and prepared adenoviruses. ANK performed semaphoring-induced collapse experiments. JY prepared and purified SEMA3E protein. SED, GB, MB, and PO performed genome sequencing and data analysis and genetic diagnosis, analyzed data, and drafted text on genetics. SM performed patient imaging and clinical phenotyping and drafted text on clinical descriptions. AS and TVP provided advice on setting up in vitro and in vivo experiments. PRR provided expertise and advice on lymphatics. JSR examined location of variants/mutations and devised hypotheses regarding their possible function. FZ critically reviewed the manuscript and prepared schematic diagrams. EYJ provided scientific input and critically reviewed the manuscript. ET initiated the project, generated research funds and ideas, and directed the project. ET, JSR, and KLP wrote the manuscript. All authors edited and approved the manuscript.

## Conflict of interest

The authors have declared that no conflict of interest exists.

## Funding support

- The National Genomic Research Library is funded by the National Institute for Health Research and NHS England.
- The Wellcome Trust, Cancer Research UK, and the Medical Research Council have also funded research infrastructure.
- This work was supported in part by grants from the following:
- British Heart Foundation (RG/F/20/110025).
- Medical Research Council (MR/W028379/1 and UKRI523).
- British Heart Foundation Centre of Excellence, Oxford (RE/13/1/30181 and RE/18/3/34214).
- Wellcome Trust grant (203141/Z/16/Z) supporting the Wellcome Centre for Human Genetics, a joint MRC/BHF programme grant (MR/P011543/1 and RG/17/7/33217).
- Fonds National Suisse de la Recherche Scientifique (FNS) (CRSII5_177191/1).

## Supplementary Material

Supplemental data

Unedited blot and gel images

Supporting data values

## Version 1 07/01/2026

Electronic publication
