# Supplementary material for: Mechanoreceptor plexin D1 regulates lymphatic valve morphogenesis and lymphedema pathogenesis
Source: J Clin Invest. 2026 Jul 1;136(13):e193385. doi: 10.1172/JCI193385 (PMC13318119; doi:10.1172/JCI193385)
Supplement: Supplemental data [file jci-136-193385-s020.pdf]

Genotyping

For embryo identification, genotyping was conducted via PCR analysis of DNA extracted from tail clippings using the Phire Tissue Kit (Thermo Scientific F140-WH,). For the *Plxnd1$^{fl/fl}$* reaction, thermo cycling condition is 94°C for 1 min, followed by 35 cycles of 94°C for 1 min, 56°C for 1 min, 72°C for 1 min and final step at 72°C for 5 min. For the *Cre* reaction, thermo cycling condition is 94°C for 3 min, followed by 35 cycles of 94°C for 30 secs, 51.7°C for 1 min and 72°C for 1 min and final step at 72°C for 2 min. Primers used for *Plxnd1$^{fl/fl}$* and *Cre* reaction are listed in Table S1.

Cell culture, transfection and shear stress

Human primary dermal lymphatic endothelial cells (LECs, Promocell, C12216) were cultured in Endothelial Cell Growth Medium MV2 medium (Promocell, C-22022) with 1% penicillin and streptomycin (Gibco, 15140122). Human aortic endothelial cells (HAECs, Promocell C-12271) were cultured in Lonza EGM2 medium with 1% penicillin and streptomycin. All *in vitro* experiments were conducted using LECs at passage 6-8. African green monkey kidney fibroblast-like cell line (COS-7) cells and human embryonic kidney 293A cells (HEK293A) were maintained in Dulbecco's Modified Eagle Medium (DMEM) (Corning, 10-013-CV) with 10% FBS and 1% penicillin and streptomycin. All cell types were cultured in a humidified incubator at 37°C with 5% CO_2. siRNA reverse transfections for Scr (siGENOME, D-001810-10; Dharmacon), *PLXND1* (SMARTpool; E-014121-00; Dharmacon), *CDK5* (SMARTpool; E003239-00; Dharmacon) were performed using the Lipofectamine RNAiMAX Reagent (Invitrogen, 13778150).

Confluent LECs grown on μ-Slide $I^{0.6}$ Luer fibronectin-coated slides ibidi; IB-80186) were subjected to lymphatic valve shear stress (+/-4 dyn/cm^2, ¼ Hz) (1, 2) using the Ibidi Pump System for the specified period. For the co-immunoprecipitation studies involving adenoviruses expressing wildtype or mutant PLXND1 or NRP1, LECs were exposed to shear stress for 10 min (3); this protocol was chosen to dynamically mechanically stimulate cells and therefore minimize confounding effects due to possible secretion or upregulation/downregulation of semaphorins. For the static condition, confluent cells were maintained for the same duration in either μ-Slide $I^{0.6}$ Luer or 6 well plates or parallel plate slides in the absence of shear stress.

RNA extraction and qPCR

Total RNA extraction was carried out from cells using the RNeasy Plus Mini kit (Qiagen, 74136), with an additional genomic RNA removal step using RNase-Free DNase set (Qiagen, 79254) according to the manufacturer's instructions. Reverse transcription was carried out using the Superscript III cDNA synthesis kit (Invitrogen, 18080051). qPCR was performed in triplicate with SYBR green (VWR International Ltd, 733-1382) using a CFX96TM real-time system. Thermocycling conditions were programmed for initial step at 95 °C for 3 min, followed by 40 cycles of 15 s of 95 °C and 45 s at 60°C. The housekeeping gene 18S rRNA was used to normalize gene expression and relative expression was calculated and plotted using the $\Delta\Delta C_t$ method. Primer sequences used for real-time PCR are listed in Table S1.

Immunofluorescence microscopy

Cells under lymphatic valve shear stress

After fixation in 4% PFA with 1mM $CaCl_2$ and 0.5mM $MgCl_2$ at RT, cells were permeabilized with 0.1% Triton X-100/PBS and blocked with 5% donkey serum/0.5%BSA/0.1% Triton X-100/PBS for 1 h at RT and incubated with primary antibodies overnight at 4°C. After washes, cells were incubated with Alexa-Fluor 405-, 488-, 568-, and 647-conjugated secondary antibodies for 1-2 h at RT and DAPI (Invitrogen) and mounted with ibidi mounting medium as previously described (4). For a complete list of antibodies see Table S2.

Surface expression of COS7 cells

350,000 COS7 cells were seeded onto glass coverslips in 6-well plates and transduced with adenoviruses expressing either wild-type PLXND1 or mutant PLXND1 Gln676Glu (Q676E), PLXND1 Ser178Asn (S178N) viruses, wild-type NRP1, mutant NRP1 Arg552Gln (R552Q) or Asp302Lys (D320K). After 48 h infection, cells were fixed in 2% formalin for 15 min at RT followed by blocking in 5% donkey serum/0.5% BSA for 1 h at RT. Immunostaining was performed by primary antibody incubation at 4°C overnight. Samples were washed in PBST and incubated with Alexa Fluor 568-, and 640 conjugated wheat germ agglutinin (Invitrogen) for 1 h at RT and DAPI (Invitrogen) and mounted with SlowFade Gold (Invitrogen, S36936). For antibodies used, refer Table S2.

Image acquisition and analysis

All immunofluorescence images were acquired using Leica SP8 confocal microscope at 20x and 63x and Zeiss LSM900 confocal microscope equipped with 405nm, 488nm, 561nm, 647nm laser lines using Plan-Apochromat 40x/1.2 objective and Airyscan 2 GaAsP-PMT detector. Identical image acquisition parameters were applied to each experimental set. All confocal images are presented as maximum intensity projections generated from Z-stacks of single tile or multiple tile scan images.

Image analysis was conducted using ImageJ FIJI. For the *in vivo* studies, quantification of number of lymphatic valves in mesenteries and dorsal embryonic skin was performed by identifying valves as PROX1 -expressing clusters of LECs within the collecting lymphatic vessels and measuring the length of vessel to obtain average of valves per vessel length or per field of view (mesentery: $n = 4$ embryos per condition; ≥ 3 vessels per mouse; 3 independent litters of mice; skin: $n = 6$ embryos from 3 different litters, 3-4 images (each image encompasses 16 tile scan Z-stacks images); mesentery: $n = 5$ P6 pups per condition from 3 different litters; 2-4 vascular branches analysed per pup (each image encompasses tile scan Z-stacks images). Quantification of α-smooth muscle actin was obtained from the mean fluorescence intensity within lymphatic valves identified as PROX1 -expressing clusters of LECs from Z- stack images (n = 3 per genotype). Different developmental stages of lymphatic valves were characterized as previously described (5). For the *in vitro* studies, quantification of nuclear immunostaining intensity, nuclei were outlined, based on DAPI staining, and the mean fluorescent intensity of the stain present in the nucleus of GATA2, PROX1, FOXC2 were obtained from maximum intensity projection images of Z-stacks ($n \leq 300$ cells per sample) and were measured in ImageJ using the 'analyze particles' function. (336-360 nuclei; $n = 3$ biological replicate). Quantification of CX37 staining was performed on maximum intensity projection images of Z-stacks ($n = 300$–350 cells analyzed; $n = 3$ biological replicates). All images were obtained and quantified in a blinded fashion.

Bead pulling/magnetic tweezer system

Tosyl-activated paramagnetic beads (4.5 µm) were firstly washed with PBS followed by coating with an antibody targeting the extracellular domain of PLXND1 (Santa Cruz), PECAM-1 (a gift from P.Newman) or NRP1 (AF3870; R&D). Beads were quenched in 0.2 M Tris, pH 7.4 in prior to force application to remove any residual tosyl groups. LECs or PLXND1-depleted LECs transduced with adenoviruses expressing wildtype PLXND1 or Q676E or S178N mutant PLXND1 were incubated with the beads for 30 min before force application using a magnet for 5– 30 min at 37 °C. In all experiments, the magnet generated the force of approximately 10pN. The cells were promptly washed with ice-cold PBS with Ca^{2+} and Mg^{2+}, snap-frozen on dry ice and immediately lysed.

SEMA3E protein preparation and purification

Mouse Sema3E1-3 (residues 26-680) was cloned into the pHLsec vector (6) with an in-frame C- terminal hexahistidine (His6) tag. Protein expression was achieved via transient transfection in HEK 293T cells (ATCC CRL-3216). The conditioned medium was collected five days post-transfection and underwent buffer-exchange using a QuixStand diafiltration system (GE Healthcare). Purification was carried out through immobilized metal-affinity chromatography (IMAC) on a HisTrap FF column (GE Healthcare), followed by size-exclusion chromatography (SEC) on a Superdex 200 16/60 column (GE Healthcare) in 10 mM HEPES (pH 7.5) and 150 mM NaCl.

SEMA3E challenge

LECs with endogenous knockdown of *PLXND1* via siRNA were transduced with either wild-type or mutant PLXND1-expressing adenoviruses Q676E or S178N. Cells were serum starved in 0.5%FBS/M199 and treated with recombinant SEMA3E at 400nM for 10 min or 30 min. Cells were assayed for western blot sing appropriate antibodies as previously described. For immunofluorescence,

subconfluent ECs seeded on fibronectin-coated coverslips were fixed in 2% formaldehyde, permeabilization was carried out in 0.2% Triton X-100 and blocking in 10% goat serum. To analyse the phosphorylation of cofilin(S3) and phosphorylation of FAK(Y397), cells were lysed as described above and lysates were immunoblotted with a primary antibody against p-cofilin(S3) and p- FAK(Y397).

Cloning and adenoviral generation

Wild-type and mutant form of PLXND1 Q676E or S178N, wild-type and mutant form of NRP1 R552Q and D320K were inserted into the pENTR/D-TOPO entry vector of the Gateway System (Invitrogen, K240020), employing the KOD Hot Start High Fidelity polymerase (Sigma Aldrich, 71086). Sanger sequencing was used to verify successful cloning and the constructs were further subcloned into the pAd/CMV/V5-Dest destination vector using LR Clonase II reaction. All steps were performed according

to the manufacturer's instructions. After PacI digestion to linearize the destination vector, HEK293A cells were transfected and used for adenoviral generation and subsequent amplification according to the manufacturer's instructions. Adenovirus purification was performed using AdEasy® Virus Purification Kit (Agilent Technologies, 240-243-1) as per manufacturer's instructions. Briefly, hLECs were treated with siRNA and subjected to adenoviral transduction on the following day. After transduction, cells were reseeded and subsequently used for shear stress, SEMA3E stimulation, or bead- pulling experiments. The amount of virus used for transduction was optimized by dose titration and confirmed by western blot analysis. No additional selection of transduced cells was performed. Experiments were conducted 72 hours after siRNA transfection and 48 hours after adenoviral transduction, corresponding to the time points of maximal knockdown and transduction efficiency.

Human exome and genome sequencing analysis

Italian cohort

Recruitment: A total of 343 Caucasian individuals diagnosed with primary lymphedema were enrolled in the study. Recruited patients underwent comprehensive clinical evaluations to confirm the diagnosis of primary lymphedema, while excluding secondary causes of the pathology, as previously described (7, 8). All patients underwent pre-test genetic counseling and provided written informed consent in accordance with the Declaration of Helsinki. Genomic DNA was extracted from approximately 5 mL of peripheral blood or saliva (9) using a commercial kit (SaMag Blood DNA Extraction Kit (Sacace Biotechnologies, Como, Italy)) according to the manufacturer's instructions. Construction of the family tree was performed using HaploPainter.

Diagnostic NGS sequencing: Samples were collected and sequenced in MAGI laboratories using a comprehensive custom panel [approximately 2.4 Mb cumulative target length (GRCh38/hg38)], that included several genes associated with primary lymphedema]. The Twist Custom Panel Design Technology (Twist Bioscience, South San Francisco, CA, USA https://www.twistbioscience.com/products/ngs accessed on 20 September 2023) was used to capture the coding exons and flanking regions of each gene of the panels, which were chosen based on literature reviews or databases [Human Gene Mutation Database (HGMD Professional), Online Mendelian Inheritance in Man (OMIM), Orphanet, NCBI GeneReviews, NCBI PubMed and specific database]. The Twist Library Preparation EF Kit and Twist Universal Adapter (UDI) System with Standard Hybridization Target Enrichment (Twist Bioscience) were used for preparation of libraries from genomic DNA samples. In short, 50 ng of genomic DNA was enzymatically fragmented into 450–550 bp fragments, end repaired and dA-Tailed. The Twist universal adaptor was ligated on the fragments, which were subsequently SPRI purified and enriched by 7 PCR cycles with Twist Unique Dual Index Primers. Hybridization to the Twist oligo probe capture library was carried out for 16 hr in a twelve- plex reaction. Following hybridization, washing, and elution, the eluted fraction underwent 9 cycles of PCR-amplification and purification. Sequencing was performed on MiSeq personal sequencer (Illumina, San Diego, CA, USA) following the manufacturer's instructions. A total of 24 pool library samples were loaded on Miseq using MiSeq V3 kit. The list of the anaylzed gene is available in reference (10). All patients tested negative for the genetic variants associated with primary lymphedema.

Targeted PLXND1 exome sequencing: Library preparation, sequences capture, sequencing and data analysis were performed by IntegraGen SA (Evry, France). Sequence capture, enrichment and elution were performed according to manufacturer's protocols (Twist Bioscience). The NEBNext® Ultra II kit (New England Biolabs®) was used to prepare libraries, according to (11). Briefly, 150 ng of genomic DNA was sonicated and purified to yield fragments of 150-200 bp before ligation of paired-end adaptor oligonucleotides, purification and enrichment by 7 PCR cycles. 500ng of purified libraries were processed for hybridization to the Twist oligo probe capture library for 16 hr in a singleplex reaction. Following washing and elution, fractions were amplified, purified and quantified by qPCR. Each eluted- enriched DNA sample was then sequenced on an Illumina NovaSeq as Paired End 100 reads. Illumina Real Time Analysis (3.4.4) was used for image analysis and base calling.

Bioinformatics and genetic variants classification: The pathogenicity of the identified genetic variants was evaluated according to the American College of Medical Genetics and Genomics guidelines (ACMG) (11), using an in-house bioinformatics pipeline (12) and online databases such as ClinVar (13), dbSNP (14), VarSome (15) and gnomAD(16) databases. Fastq (forward-reverse) files were generated after sequencing. Bioinformatic analysis of genomic data was conducted as previously reported (17, 18). In summary, the sequencing reads were aligned to the reference genome (hg38/GRCh38) using Burrow-Wheeler Aligner (version 0.7.17-r1188) software. Duplicates were removed using SAMBAMBA (version 0.6.7) and MarkDuplicates GATK (version 4.0.0.0). The BAM alignment files were refined through local realignment

and base quality score recalibration using the RealignerTargetCreator and IndelRealigner tools from GATK. Minor allele frequencies (MAF) were obtained from the gnomAD database(16).

UK cohort

Recruitment: 295 proband with lymphatic related disorders, who had been exome sequenced, were available for analysis. They were recruited through genetic and lymphovascular clinics in the UK, as well as through international collaboration. Ethical approval for this study was obtained from the local Research Ethics Committee (REC Ref: 05/Q0803/257 and 12/LO/0498). Written, informed consent was obtained for all subjects. All affected individuals and family members underwent a detailed physical examination. The subjects had been tested clinically on a targeted panel for primary lymphoedema and due to a negative result genomic DNA was passed on to our research study. An additional 164 individuals were identified through analysis of data from the Genomics England 100,000 Genomes Project (19), selecting probands recruited under the "normalised specific disease" category: primary lymphoedema, Meige, or Milroy and checking for continued consent (main-programme v19). Samples were excluded with a GMC exit questionnaire "case solved family" status "Yes".

Sequencing and Processing: Exome sequencing was performed by a variety of vendors and processed according to the Genome Analysis Toolkit (GATK, gatk-4.0.4.0/) best practices**.** Briefly, Fastq files were obtained and aligned to the reference genome (hg38/GRCh38) using Burrow-Wheeler Aligner software (v0.7.17-r1194). Duplicates were removed using Picard Tools followed by (sample-paired) local realignment around indels and germline variant calling with HaplotypeCaller. Joint genotyping across all samples was undertaken using GenotypeGVCFs to produce a cohort-wide VCF file. Variant annotation was performed using the Variant Effect Predictor (VEP v112) to determine impact on canonical transcripts and population level frequencies (gnomad v3.1.2). Whole genome sequencing was analysed through the Genomics England aggV2 dataset (VEPv99, gnomAD r2.1) to identify rare (gnomADg AF < 0.0001) variants in *PLXND1,* impacting on the canonical transcript (ENST00000324093), with a VEP defined impact of "high" or, for "moderate" impact variants, requiring Phred scaled CADD score >15. Following quality control and exclusion of samples with likely pathogenic variants in established genes, 272 and 163 probands were available for analysis, for the UK exomes and Genomics England dataset respectively.

Human *PLXND1* variant confirmation by Sanger sequencing

Genetic variants identified in *PLXND1* were confirmed by Sanger sequencing on a CEQ8800 Sequence (or by SourceBioScience using the PeakTrace™ DNA sequencing basecaller software on the Applied BioSystems 3730 DNA analyzer. No DNA was available to confirm the Val612Met variant. Primers sequences and sequencing conditions are available on request.

**Gene name**	**Application**	**Sequences**
CX37	qRT-PCR	5’-GAGCCAACTTACCCCAACCT-3’<br>5’-CCCCTTGACAGGGCCAAATC-3’,
GATA2	qRT-PCR	5’-CAAGCCCAAGCGAAGACTGT-3’<br>5’-CTTCATGGTCAGTGGCCTGTT-3’
FOXC2	qRT-PCR	5’- TGTTCGAGAACGGCAGCTT-3’<br>5’- CGCTCTTGATCACCACCTTCTT-3’
PROX1	qRT-PCR	5’-AGGACTCTGTCGGAAGGTCA-3’<br>5’-CTTGCGACATGGCAGTGTTC-3’
PLEXIND1	qRT-PCR	5’-GGCCGAGTGAAAGACTTGGA-3’<br>5’-GGGGCTTGTCTTCACGGATA-3’
18S	qRT-PCR	5′-AGGAATTGACGGAAGGGCACCA-3,<br>5′-GTGCAGCCCCGGACATCTAAG-3′.
Plexind1$^{fl/fl}$	Genotyping	5’- ACAGGTGTGTGCTCAAGGCCACCTC-3’<br>5’-CAGCCCTATAGTTCTCCACCAAAGA-3'
Cre (Transgene)	Genotyping	5’-GCG GTC TGG CAG TAA AAA CTA TC-3’<br>5’-GTG AAA CAG CAT TGC TGT CAC TT-3’
Cre (Internal positive control)	Genotyping	5’-CTA GGC CAC AGA ATT GAA AGA TCT-3’<br>5’-GTA GGT GGA AAT TCT AGC ATC ATC C-3’

Table S1: Primers sequences used for genotyping and qRT-PCR

Target	Cat. Number	Vendor	Dilution
PROX1	AF2727	R&D	1: 200
PROX1	11-002	AngioBio	1: 200
VE-cadherin	555289	BD Pharmingen	1: 200
NRP1	AF3870	R&D	1: 200
NRP1	AF566	R&D	1: 200
NRP2	AF567	R&D	1: 200
VEGFR2	AF644	R&D	1: 200
VEGFR3	AF743	R&D	1: 200
FOXC2	AF6989	R&D	1: 200
Integrin alpha 9	AF3827	R&D	1: 200
CX37	40-4300	Invitrogen	1: 100
GATA2	AF2046	R&D	1: 200
Smooth muscle actin	A5228	Sigma	1:300
PLXND1	PA5-47012	Thermo Fisher	1:150
PLXND1	CSB-PA659725LA01HU	CUSABIO	1:100
t-vinculin (V9131; Sigma-Aldrich)	V9131	Sigma-Aldrich	1:100
Alexa Fluor 488-conjugated phalloidin	A12379	Life Technologies Ltd	1:200
Alexa Fluor 640-conjugated wheat germ agglutinin CF® 640R conjugate	29026-1	BIOTIUM	1:200
Alexa Fluor 488 Donkey Anti-Goat	A11055	Invitrogen	1:300
Alexa Fluor 488 Donkey Anti-Sheep	A11015	Invitrogen	1:300
Alexa Fluor 568 Donkey Anti-Rabbit	A10042	Invitrogen	1:300
Alexa Fluor 568 Donkey Anti-Goat	A11057	Invitrogen	1:300
Alexa Fluor Plus 405 Donkey Anti-Rat	A48268	Invitrogen	1:300
Alexa Fluor 647 Donkey Anti-Goat	A21447	Invitrogen	1:300

Table S2: Antibodies used for immunofluorescence.

Target	Cat. Number	Vendor	Dilution
phosphorylated (p)-ERK1/2$^{(T202/Y204)}$	9106	Cell Signaling Technology (CST)	1:500
Total (t)-ERK1/2	9102	Cell Signaling Technology (CST)	1:1000
FOXC2	AF5044	R&D	1:1000
Phosphoserine	AB1603	Millipore UK Ltd	1:500
Phospho-CDK5 (Tyr15)	94254	Cell Signaling Technology (CST)	1:500
CDK5	AHZ0492	Life Technologies Ltd	1:1000
CDK5	MA547707	Thermo Fisher Scientific	1:1000
CDK5	14145S	Cell Signaling Technology	1:1000
PLXND1	Custom		1:500
PLXND1	PA5-47012	Thermo Fisher	1:500
NRP1	AF3870	R&D	1:1000
NRP2	AF2215	R&D	1:1000
VEGFR2	AF644	R&D	1:1000
VEGFR3	AF349	R&D	1:1000
Integrin alpha 9	MAB4574	R&D	1:1000
Phospho-cofilin^{S3}	3311S	Cell Signaling Technology (CST)	1:500
Cofilin	5175S	Cell Signaling Technology (CST)	1:1000
Phospho-FAK^{Y397}	611806	BD	1:500
FAK	3285S	Cell Signaling Technology (CST)	1:1000
Goat anti-Rabbit IgG (H+L) Cross-Adsorbed Secondary Antibody, Alexa Fluor™ 790	A11369	Invitrogen	1:10000
Goat anti-Mouse IgG (H+L) Cross-Adsorbed Secondary Antibody, Alexa Fluor™ 680	A21057	Invitrogen	1:10000

Table S3: Antibodies used for western blotting and co-immunoprecipitation. For immunoprecipitation, 1µg of the antibody was used per reaction.

figure S1

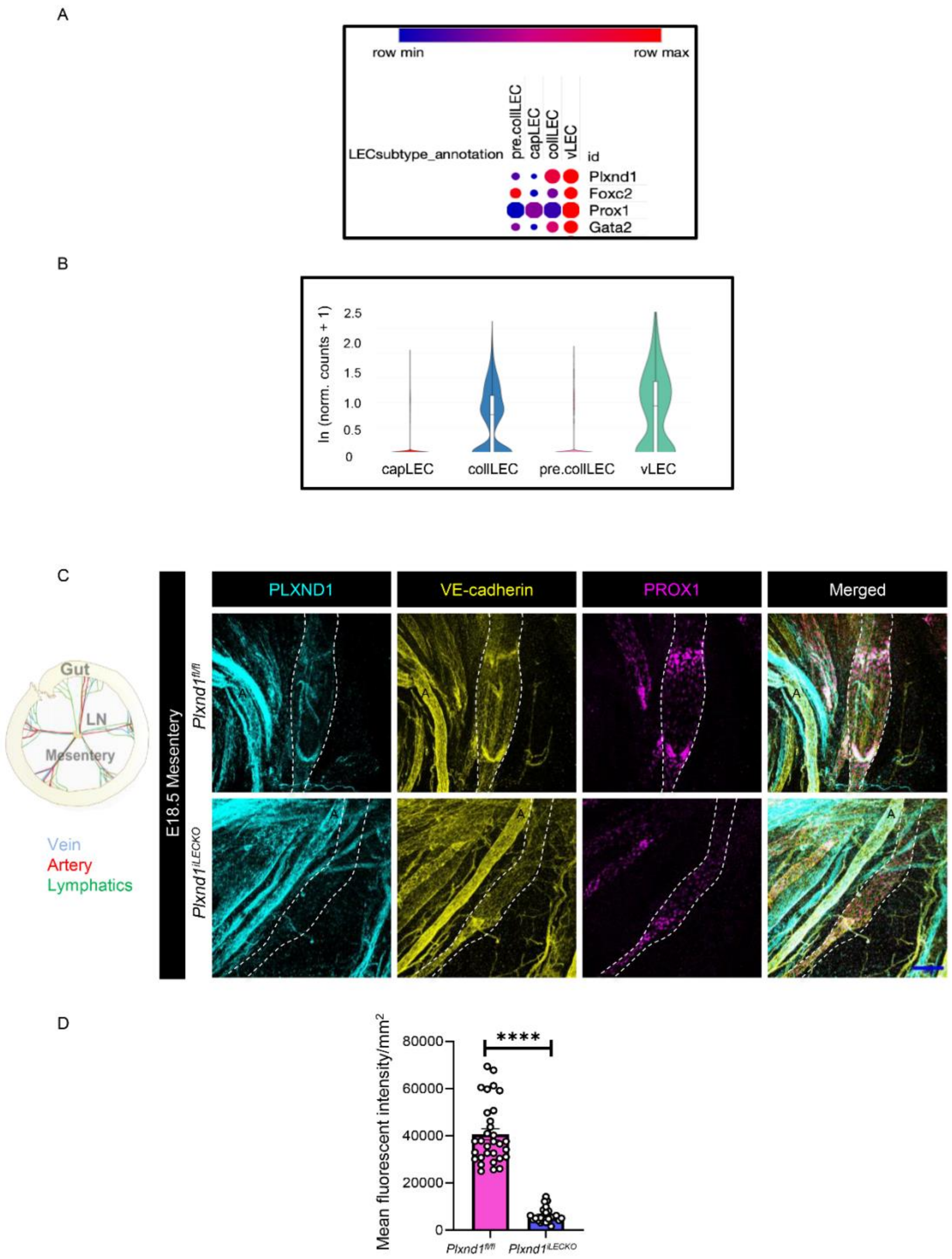

Fig. S1

***Plxnd1* is highly expressed in valve LECs. (A)** Dot plot of known lymphatic markers (*Foxc2, Prox1, Gata2*) and *Plxnd1* for LEC clusters: capillary (capLEC), collecting (collLEC), precollecting and collecting (pre/collLEC), and valve (vLEC). The color code indicates scaled average expression level in each cluster, and the dot size indicates the percentage of cells in each cluster expressing the given gene. **(B)** Violin plots showing *Plxnd1* expression in different LEC populations. **(C)** PLXND1 deletion in mouse embryos. Whole mount staining of E18.5 mesentery from $Plxnd1^{fl/fl}$ and $Plxnd1^{iLECKO}$ embryos for PLXND1 (cyan), VE-cadherin (yellow) and PROX1 (magenta). Scale bar: 100µm. **(D)** Quantification of PLXND1 staining intensity from embryos in C. (n = 8- 10 lymphatic valves analysed per embryo; n = 3 per genotype, data are mean S.E.M; Mann-Whitney test, ****$p < 0.0001$)

figure S2

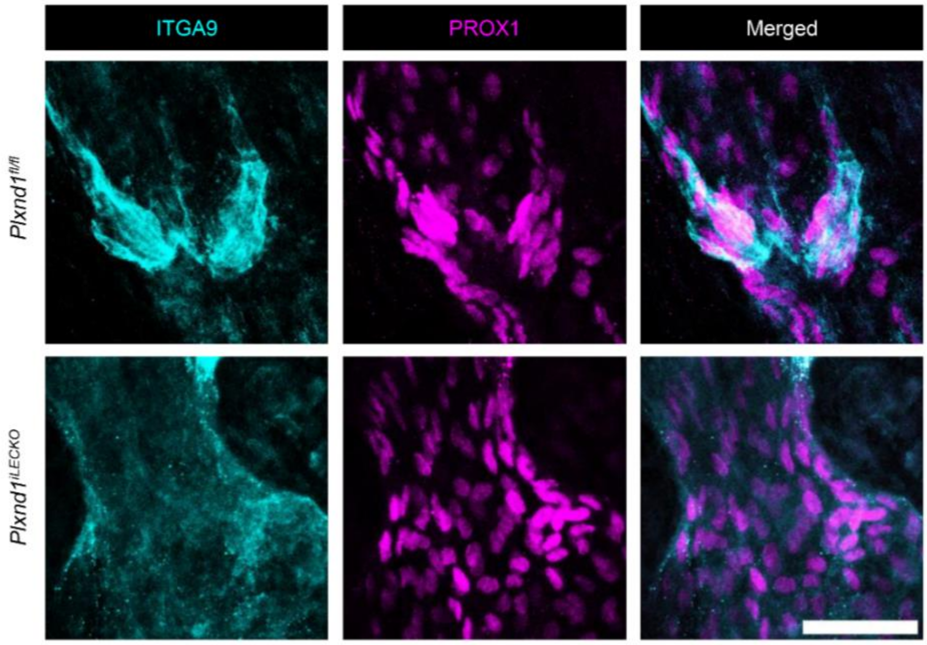

Fig. S2
ITGA9 staining in *Plxnd1$^{fl/fl}$* and *Plxnd1^{iLECKO}* lymphatic valves. Whole mount immunostaining of E18.5 mesentery from *Plxnd1$^{fl/fl}$* and *Plxnd1^{iLECKO}* embryos stained for ITGA9 (cyan) and PROX1 (magenta). *n*=3 embryos/genotype. Scale bar: 50µm.

figure S3

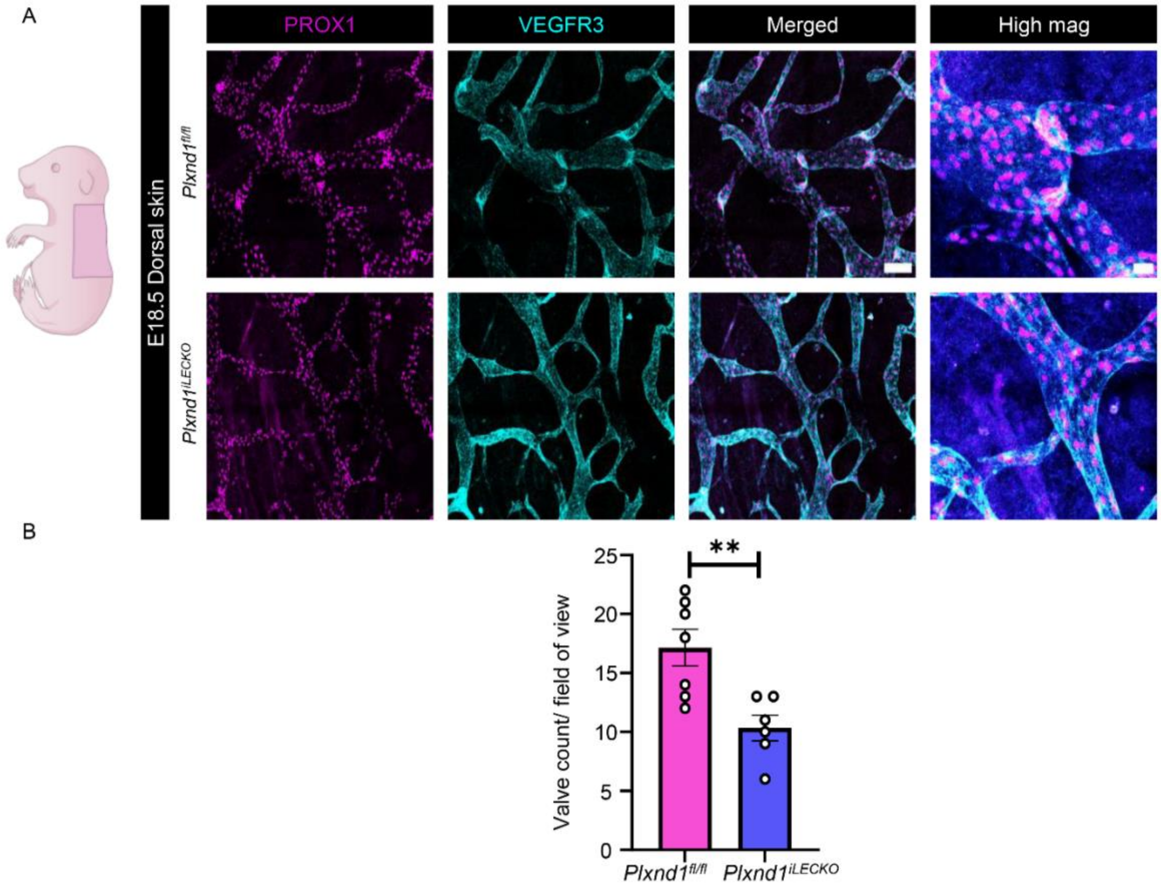

Fig. S3

Lymphatic vessel organization in embryonic dorsal skin. (**A**) Whole mounts of E18.5 dorsal skin from *Plxnd1*$^{fl/fl}$ and *Plxnd1*^{iLECKO} embryos stained for PROX1 (magenta) and VEGFR3 (cyan). Scale bar: 100µm or 40µm in magnified image**.** (**B**) Quantification of lymphatic valves per field of view in *Plxnd1*$^{fl/fl}$ and *Plxnd1*^{iLECKO} E18.5 skin, (n = 7 *Plxnd1*$^{fl/fl}$ and n = 6 *Plxnd1*^{iLECKO}, data are mean ± S.E.M; two-tailed Student's t-test, $^{**}p < 0.01$).

figure S4

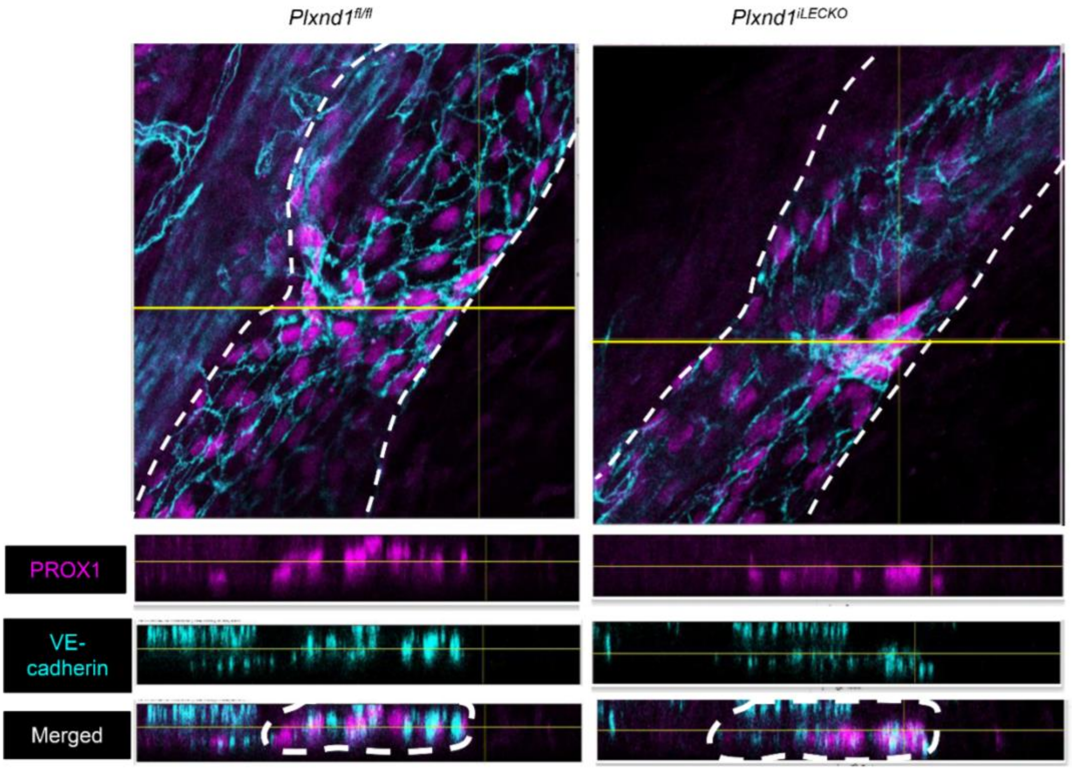

Fig. S4

Optical cross sections of valve forming regions (VFRs) in E18.5 mesentery in *Plxnd1$^{fl/fl}$* and *Plxnd1^{iLECKO}* embryos. Representative images of VFRs in E18.5 mesentery in *Plxnd1$^{fl/fl}$* and *Plxnd1^{iLECKO}* embryos. Yellow lines indicate optical cross sections of PROX1 (magenta) and VE-cadherin (cyan). *n*=3/genotype.

figure S5

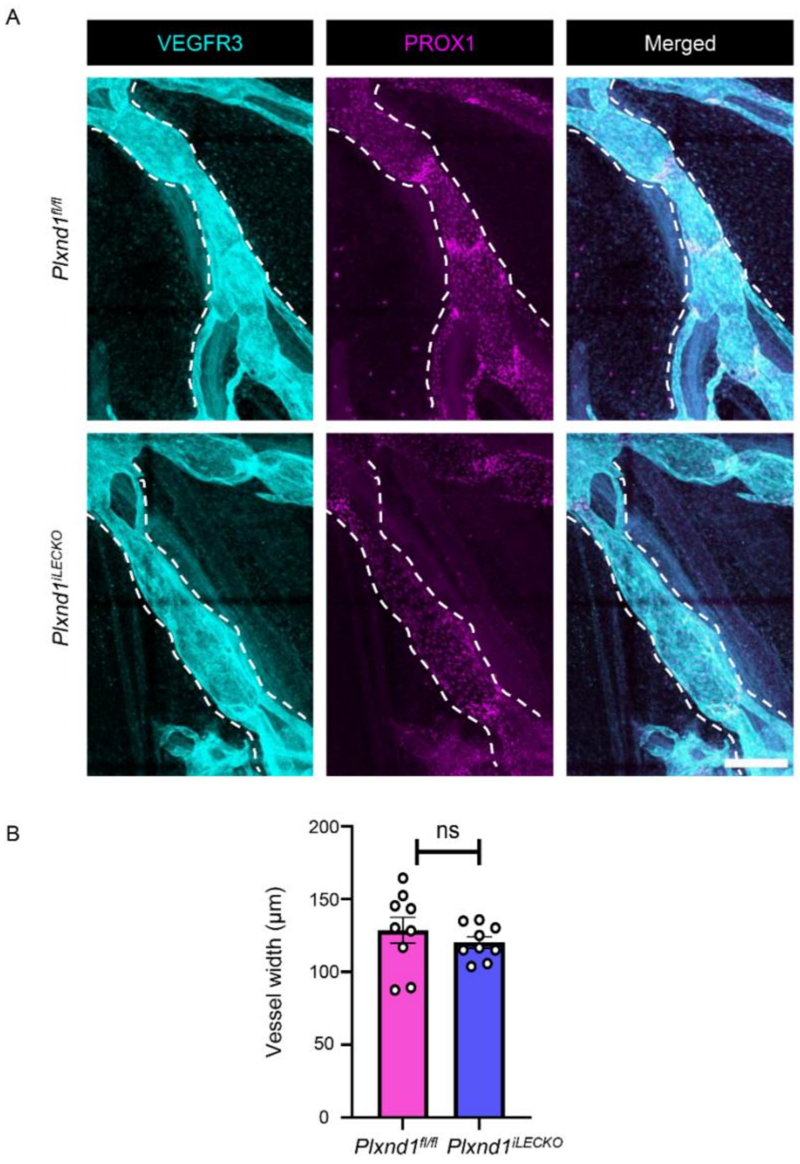

Fig. S5

Mesenteric lymphatic vessel organization at E18.5. (A) Whole mount staining of E18.5 mesentery from *Plxnd1*$^{fl/fl}$ and *Plxnd1*^{iLECKO} embryos for VEGFR3 (cyan) and PROX1 (magenta). **(B)** Quantification of lymphatic vessel width of *Plxnd1*$^{fl/fl}$ and *Plxnd1*^{iLECKO} embryos. (n = 9 vessels from 3 *Plxnd1*$^{fl/fl}$ and n = 9 vessels from 3 *Plxnd1*^{iLECKO} embryos; data are mean ± S.E.M; two-tailed Student's t-test). Scale bar: 200µm.

figure S6

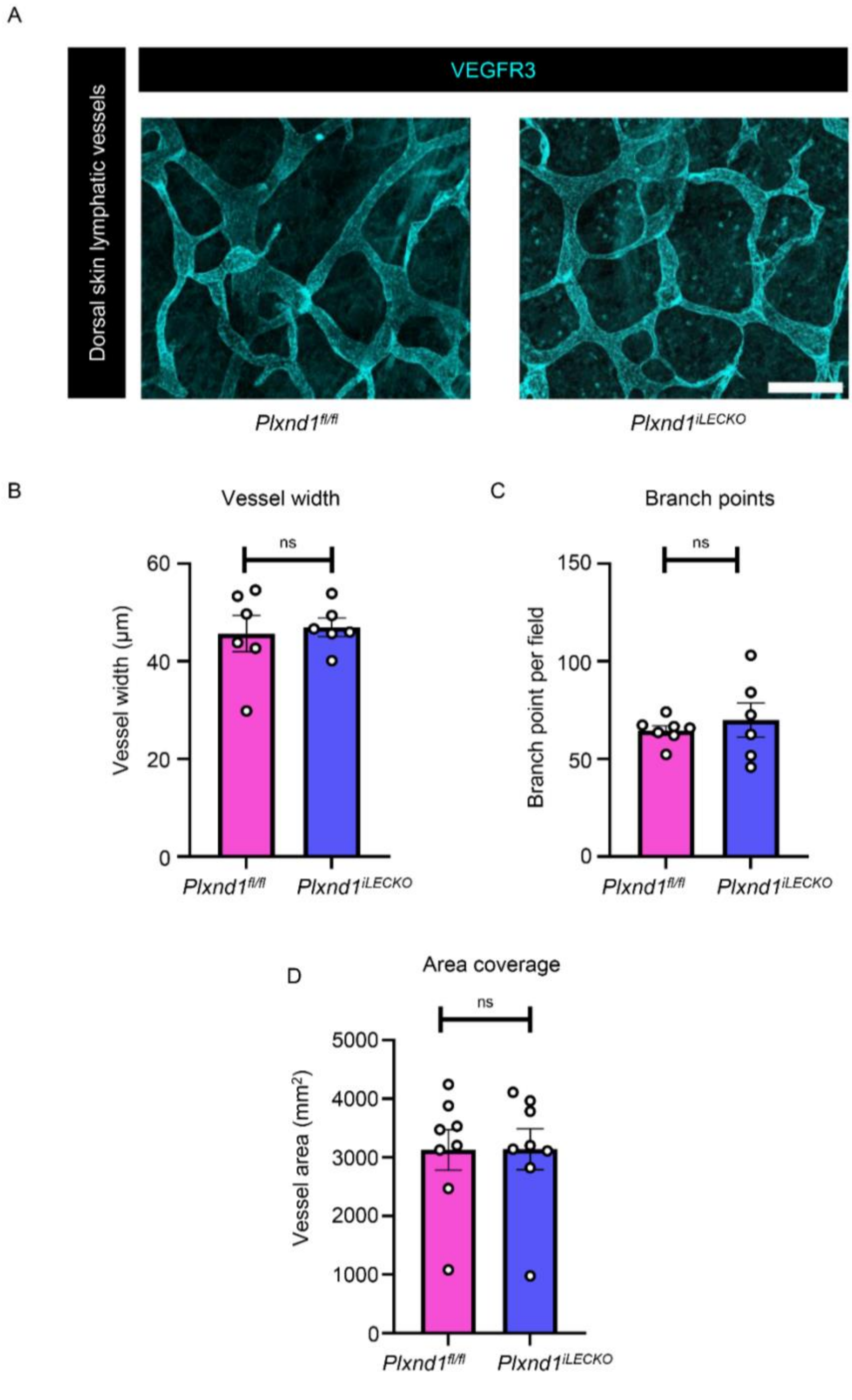

Fig. S6

Lymphatic network organization in *Plxnd1*^{iLECKO} embryos. (A) Whole mount staining of E18.5 dorsal skin from *Plxnd1*$^{fl/fl}$ and *Plxnd1*^{iLECKO} embryos for VEGFR3 . Quantification of vessel width **(B)**, branch points **(C)** area coverage **(D)** of lymphatic vessels in embryonic dorsal skin of *Plxnd1*$^{fl/fl}$ and *Plxnd1*^{iLECKO} embryos. Scale bar: 200µm, (*n* = 6-8, data are mean ± S.E.M; two-tailed Student's t-test**).**

figure S7

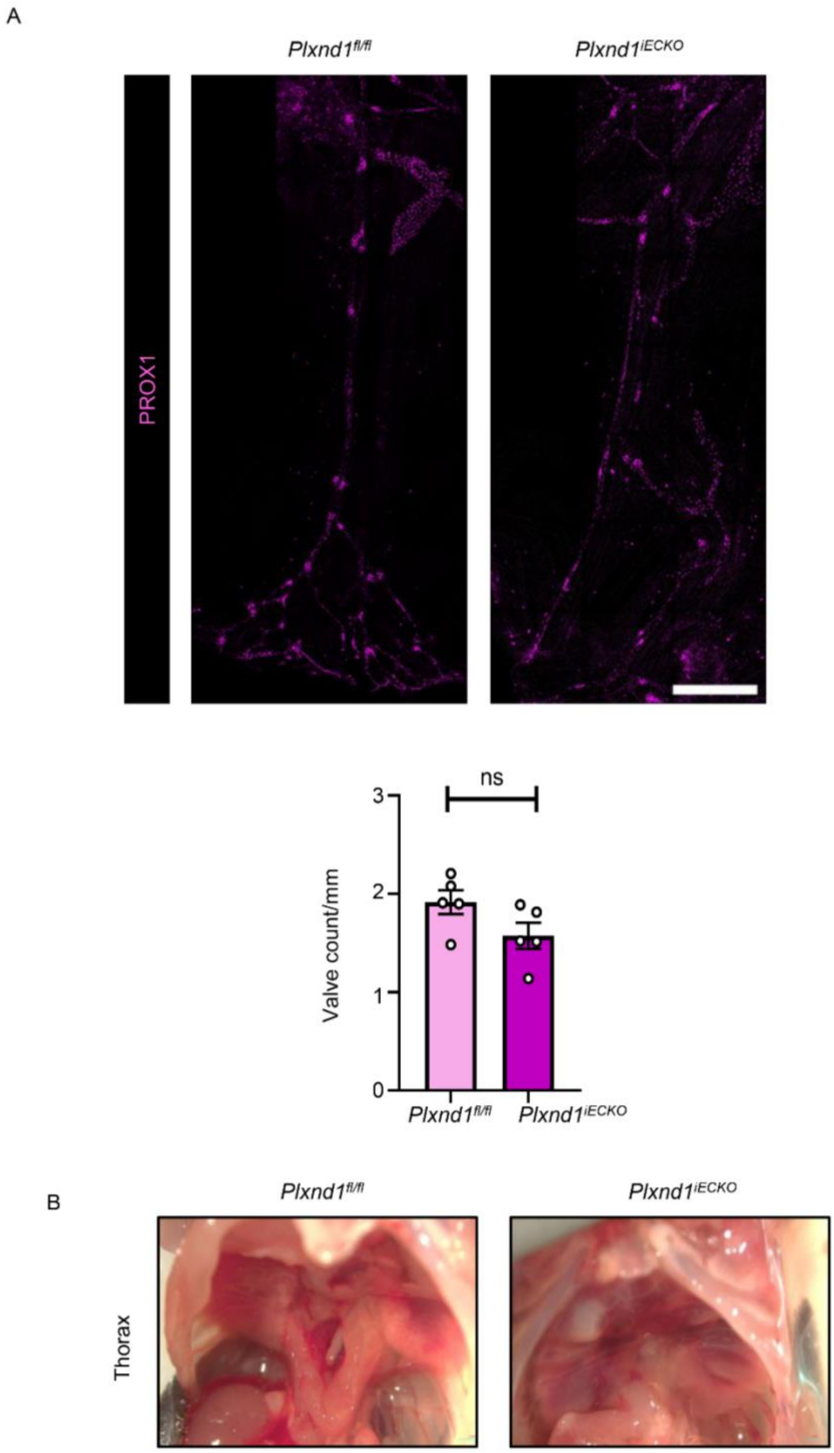

Fig. S7

Postnatal PLXND1 deletion in mesenteric lymphatics. (A) Whole mount staining of P6 mesentery from *Plxnd1*$^{fl/fl}$ and *Plxnd1*^{iECKO} embryos for PROX1 (magenta). Scale bar: 500µm. Quantification of number of lymphatic valves per millimetre of lymphatic collecting vessel in *Plxnd1*$^{fl/fl}$ and *Plxnd1*^{iECKO} P6 mesenteries, (n = 5, data are mean ± S.E.M; two-tailed Student's t-test). **(B)** Gross morphology of thorax region in *Plxnd1*$^{fl/fl}$ and *Plxnd1*^{iECKO} P6 pups (n= 6/genotype).

figure S8

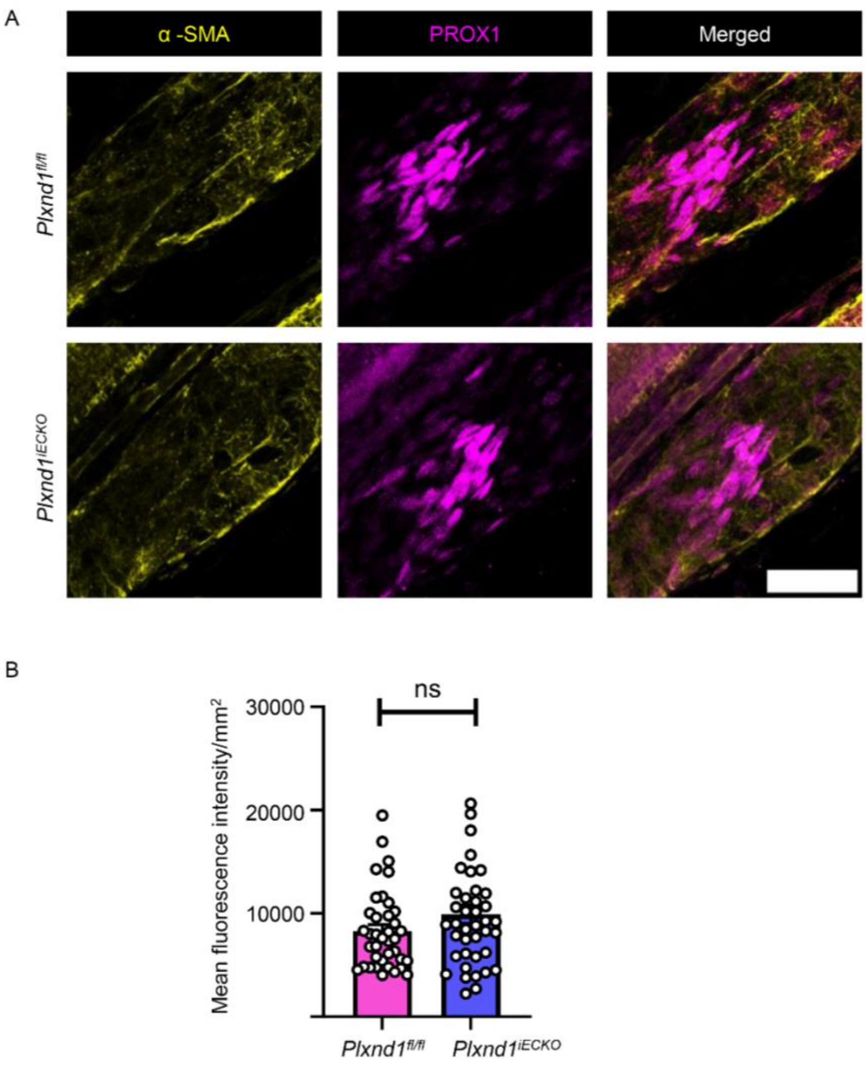

Fig. S8

Smooth muscle coverage in *Plxnd1$^{fl/fl}$* and *Plxnd1^{iECKO}* pups. (A) Representative images of lymphatic valves stained with alpha-smooth muscle actin (yellow) and PROX1 (magenta). Scale bar: 50µm. **(B)** Quantification of alpha-smooth muscle actin coverage in *Plxnd1$^{fl/fl}$* and *Plxnd1^{iECKO}* pups (*n* = 10-15 lymphatic valves per pups analysed; *n* = 3 pups per genotype, data are mean ± S.E.M; two-tailed Student's t-test).

figure S9

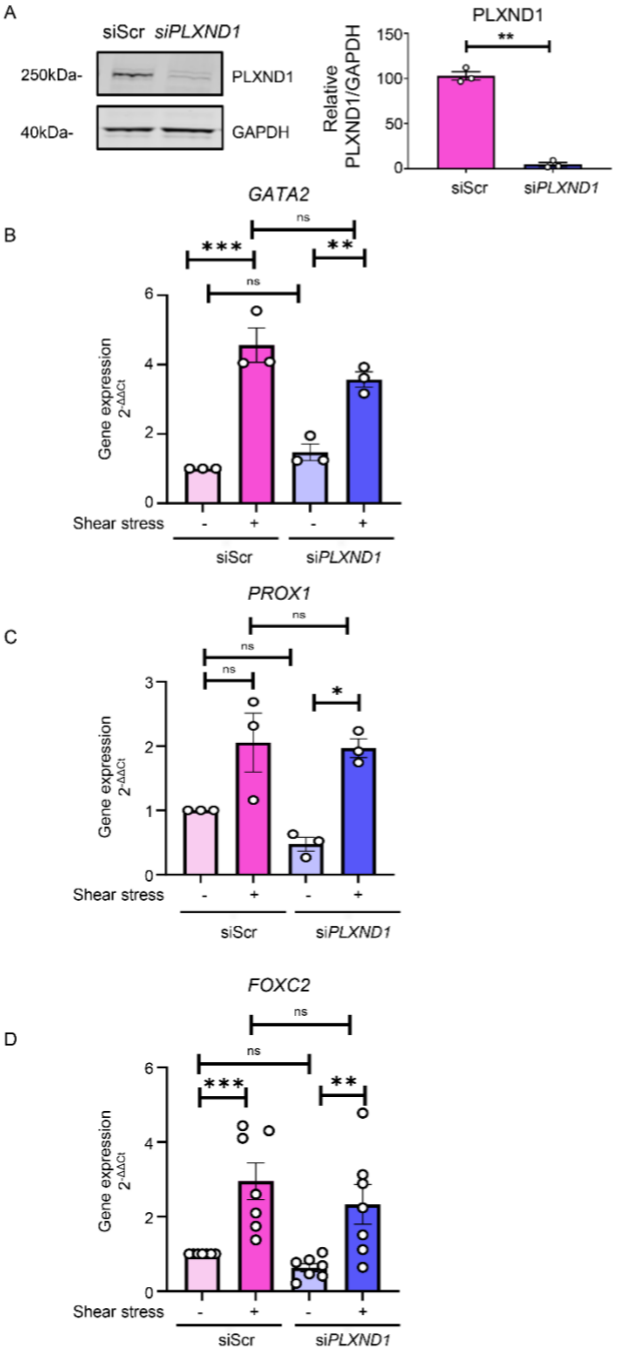

Fig. S9

Effect of *PLXND1* silencing on key lymphatic genes. (A) HLECs were transfected with scramble or *PLXND1* siRNAs before western blotting for PLXND1 and GAPDH. Quantification of *PLXND1* knockdown normalized to GAPDH is shown ($n = 3$, data are mean ± S.E.M; two-tailed Student's t-test; $**p < 0.01$). **(B-D)** HLECs transfected with either scramble or *PLXND1* siRNAs were left static or exposed to shear stress for 48h and qPCR was performed to quantify expression of *GATA2, PROX1* and *FOXC2* relative to each static condition, ($n = 3$-7, data are mean ± S.E.M; two-way ANOVA; Tukey's multiple comparisons test, $*p < 0.05$, $**p < 0.01$, $***p < 0.001$).

figure S10

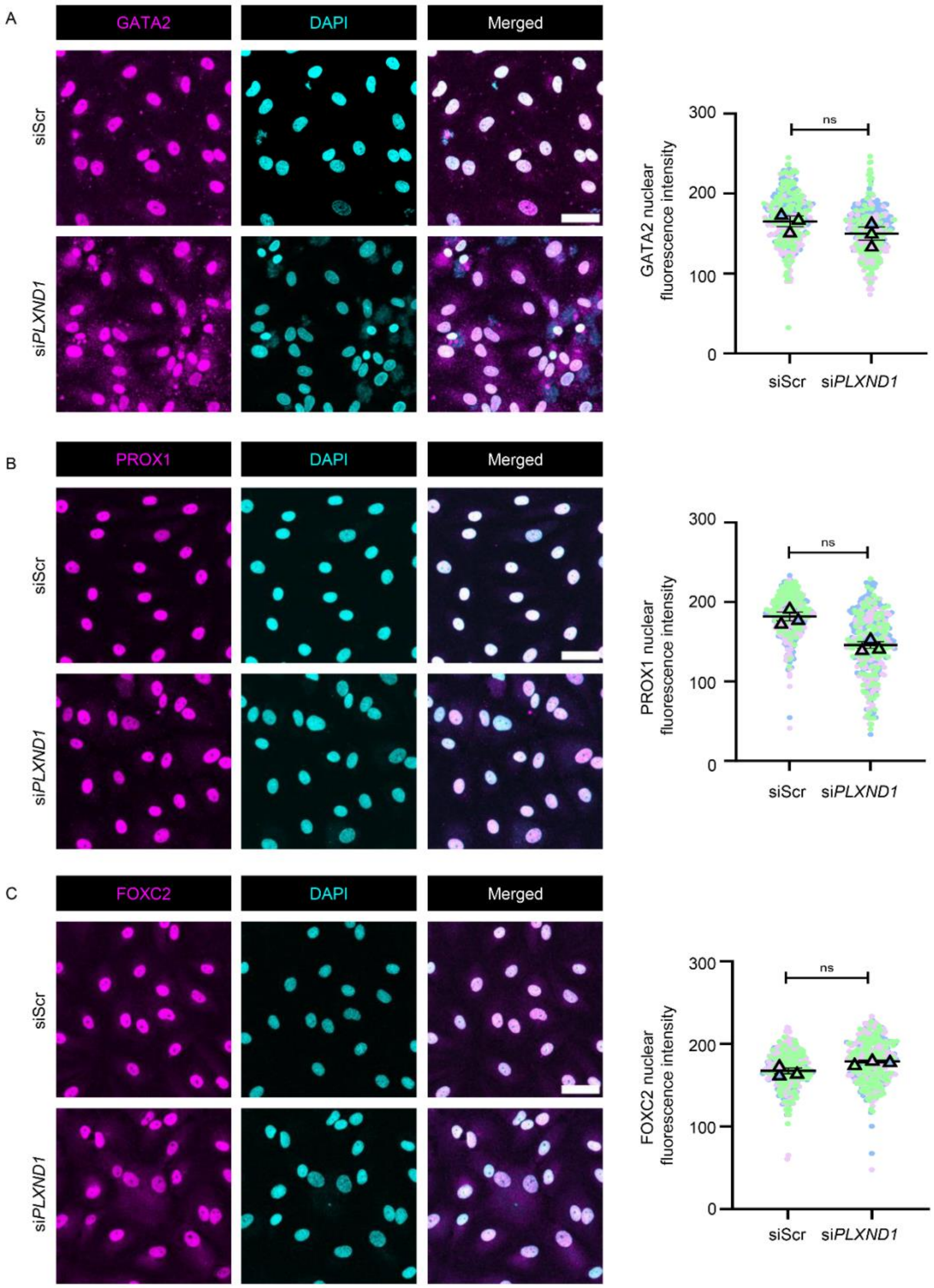

Fig. S10

Loss of PLXND1 does not alter the nuclear accumulation of key lymphatic proteins. HLECs transfected with either scramble or *PLXND1* siRNAs were exposed to shear stress for 48h and immunostaining was performed for GATA2 **(A)** PROX1 **(B)** and FOXC2 **(C)** and DAPI. Scale bar: 20μm. Quantification of nuclear intensity; n ≥ 100 cells were analysed for each *n*. (*n* = 3 biological replicates, >100 cells/biological replicate, data are mean ± S.E.M. two-tailed Student's t-test).

figure S11

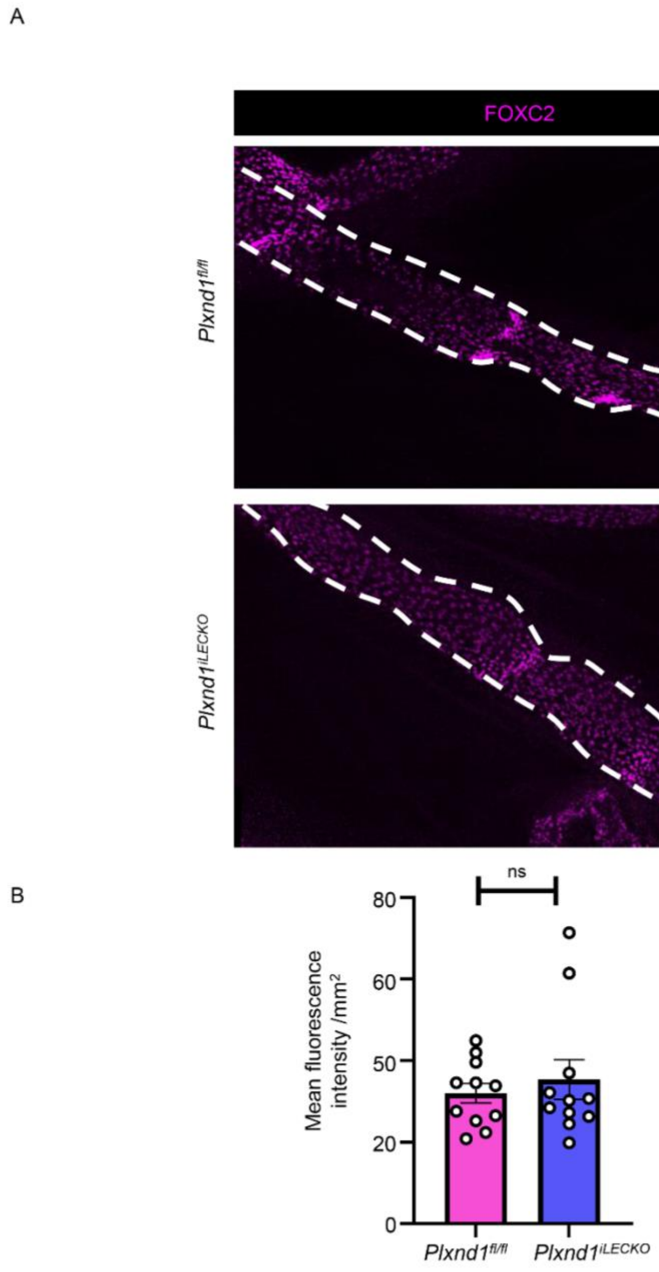

Fig. S11

FOXC2 staining in *Plxnd1$^{fl/fl}$* and *Plxnd1^{iLECKO}* embryos. (A) Whole mount staining of FOXC2 in mesentery of *Plxnd1$^{fl/fl}$* and *Plxnd1^{iLECKO}* E18.5 embryos. **(B)** Quantification of FOXC2 staining in the lymphatic vessel. Scale bar: 200µm. (*n* = 3-4 vascular branches analysed from each embryo, *n* = 3 *Plxnd1$^{fl/fl}$* and *n* = 3 *Plxnd1^{iLECKO,}* data are mean ± S.E.M. two-tailed Student's t-test*)*

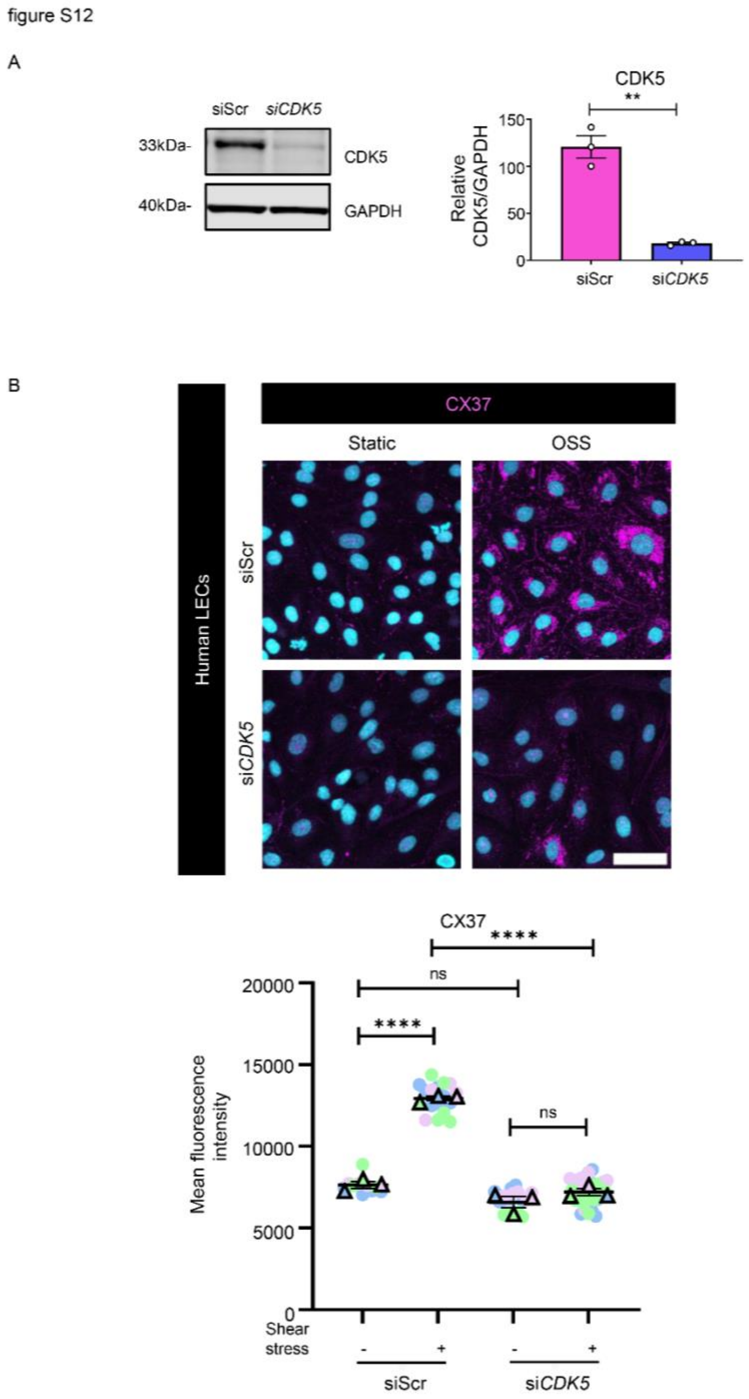

Fig. S12.

Expression of CX37 under shear stress upon *CDK5* knockdown. (A) Knockdown of CDK5. HLECs were transfected with scramble or *CDK5* siRNAs before western blotting for CDK5 and GAPDH. Quantification of *CDK5* knockdown normalized to GAPDH is shown ($n = 3$, data are mean ± S.E.M; two-tailed Student's t-test, $^{**}p < 0.01$). **(B)** HLECs were transfected with scramble or *CDK5* siRNAs were left static or exposed to shear stress for 48h. Cells were fixed and immunostained to quantify expression of CX37 at protein level (300-350 cells analysed per condition; $n = 3$ biological replicates; two-way ANOVA; Tukey's multiple comparisons test, $^{*}p < 0.05$, $^{****}p < 0.0001$) Scale bar: 50µm.

figure S13

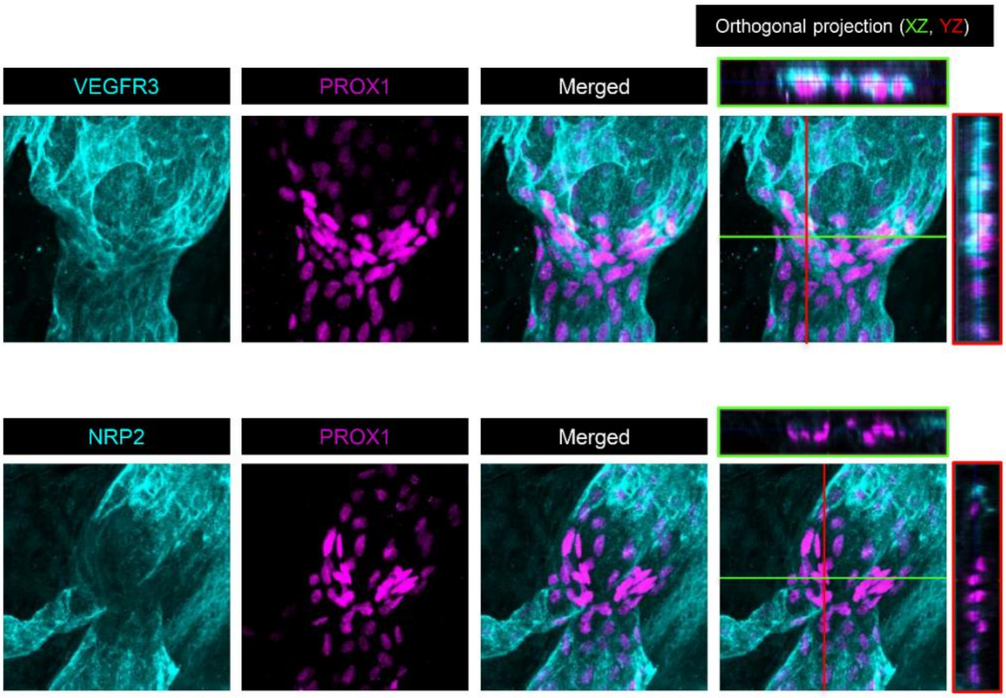

Fig. S13

Immunostaining for VEGFR3 and NRP2 in lymphatic valves. Whole mount immunostaining of E18.5 mesentery lymphatic valves for VEGFR3 and NRP2 and co-staining for the lymphatic valve marker PROX1 (*n* = 3. Scale bar: 40µm). Orthogonal views in two planes are also shown.

figure S14

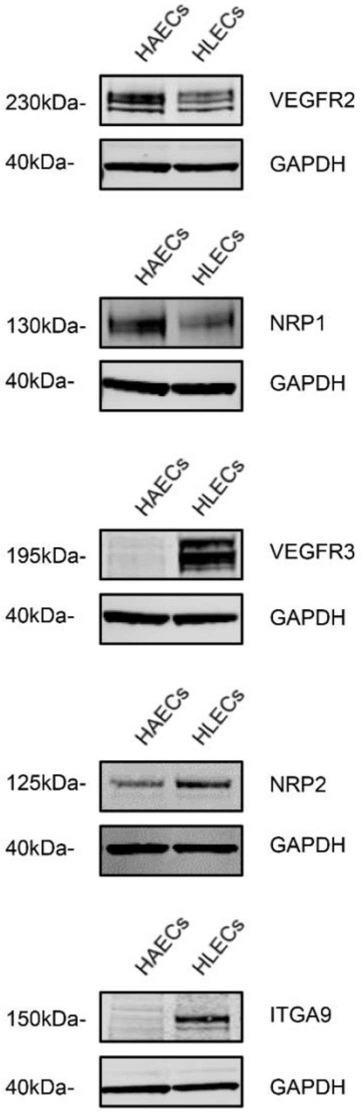

Fig. S14
Expression of co-receptors in HLECs vs HAECs. HLECs and human aortic ECs (HAECs) were lysed and processed for western blotting to assay expression levels of NRP1, NRP2, VEGFR2, VEGFR3, and ITGA9 relative to loading control GAPDH (n = 3).

figure S15

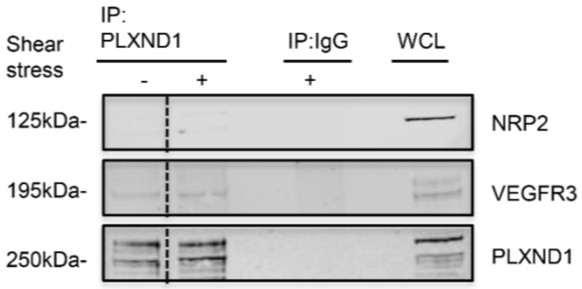

Fig. S15
PLXND1 does not associate with NRP2 or VEGFR3 in response to shear stress. HLECs were left as static or exposed to shear stress for 24h before immunoprecipitating PLXND1 and analysing for the presence of VEGFR3 and NRP2. Immunoprecipitation with non-specific IgG was used as a control (n = 2). Dashed line indicates spliced image.

figure S16

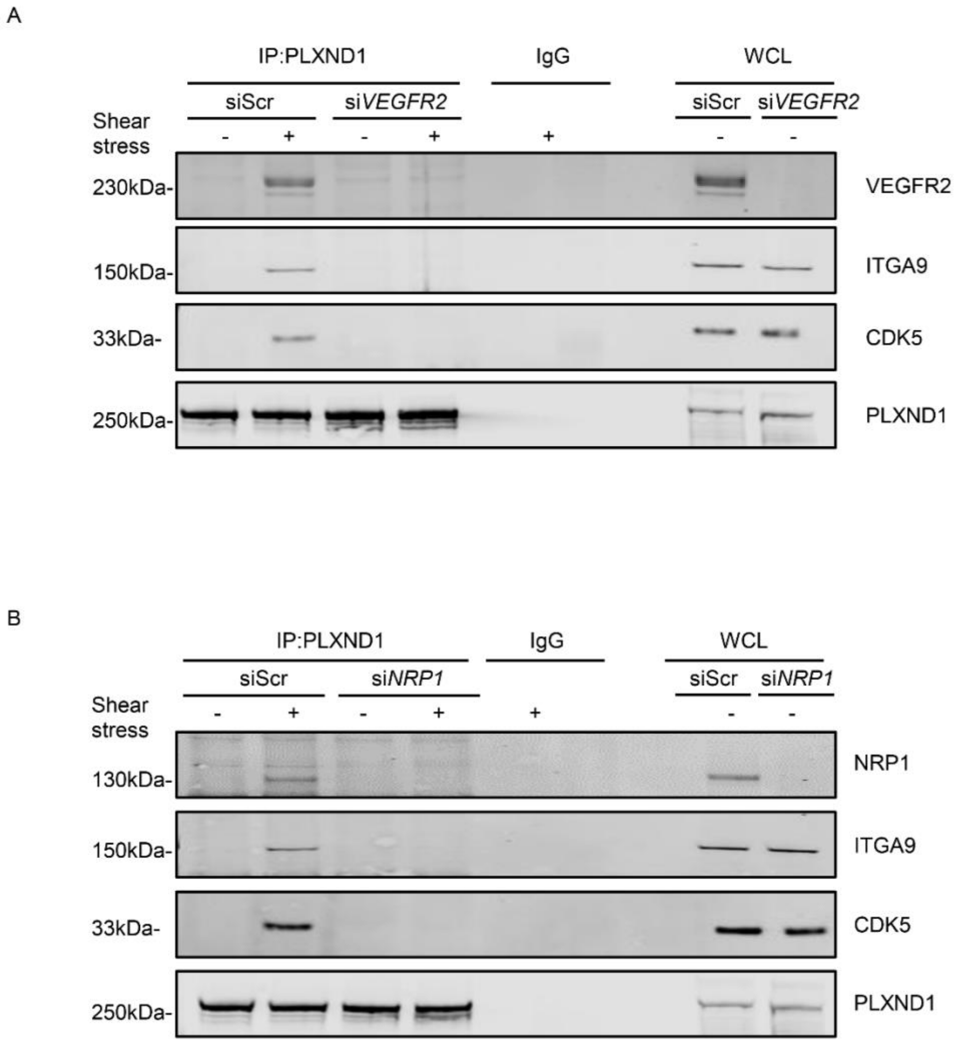

Fig. S16

Loss of lymphatic mechanosensory complex assembly under shear stress after *VEGFR2* or *NRP1* knockdown. (A) HLECs transfected with si*Scr* and si*VEGFR2* were left as static or exposed to shear stress for 24h before immunoprecipitating PLXND1 and analyzing for the presence of VEGFR2, ITGA9, CDK5 and PLXND1 (*n* = 2). Immunoprecipitation with non-specific IgG was used as a control. WCL: whole cell lysate. **(B)** HLECs transfected with si*Scr* and si*NRP1* and were left as static or exposed to shear stress for 24h before immunoprecipitating PLXND1 and analyzing for its association with NRP1, ITGA9, CDK5 or PLXND1 respectively. Immunoprecipitation with non-specific IgG was used as a control. WCL: whole cell lysate.

figure S17

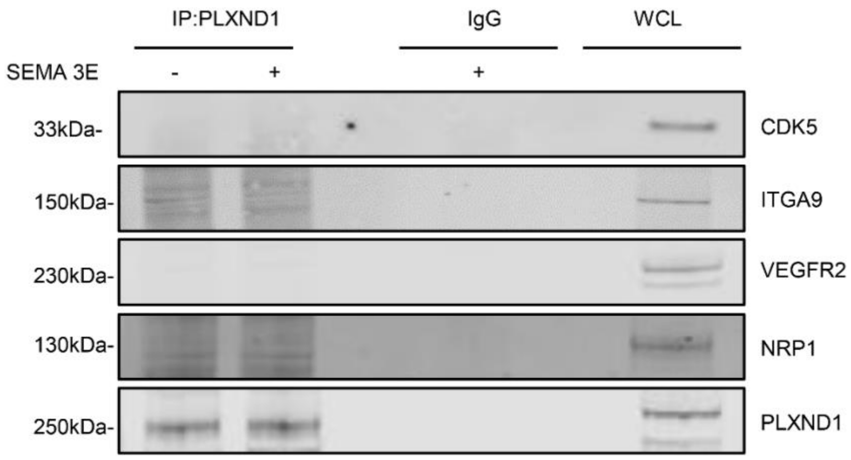

Fig. S17
SEMA3E does not induce formation of the lymphatic mechanocomplex. HLECs were treated with SEMA3E at 400nM for 10 min before immunoprecipitating PLXND1 and analyzing for the presence of VEGFR2, ITGA9, NRP1, CDK5 and PLXND1. Immunoprecipitation with non-specific IgG was used as a control. WCL: whole cell lysate.

figure S18

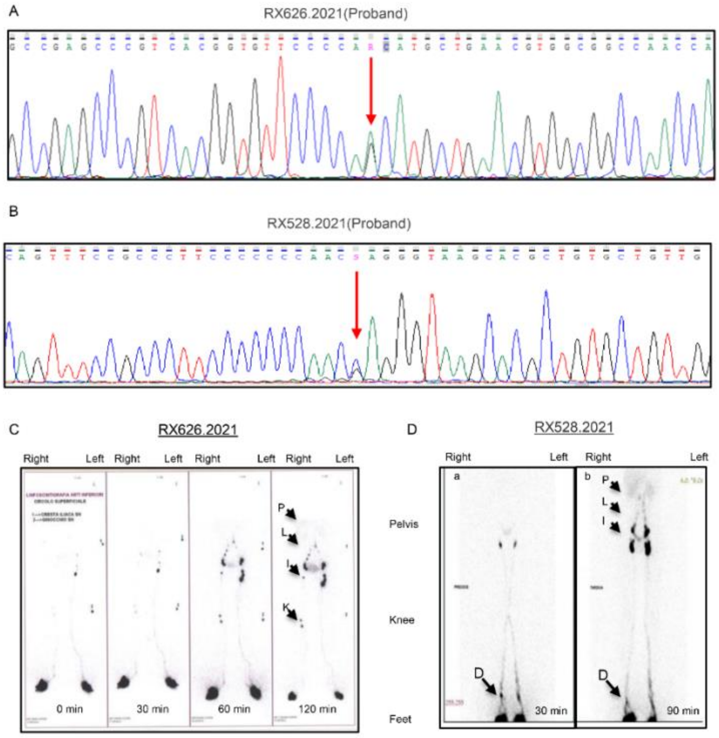

E

Clinical presentation of patients carrying PLXND1 variants						
Patient ID	Gender	Age at the visit	Age of onset	Location of lymphedema	Stage of lymphedema	Comorbidities
RX626.2021	F	65	59	Lower limbs	II	Neuroma of Morton's; Allergy
RX528.2021	M	10	0	Lower limbs	I	Mild motor dyspraxia with mild dysgraphia; Dust mite allergy; Foramen ovale pervio until first year of life

Fig. S18

Clinical details of primary lymphedema patients. Sanger sequencing of RX626.2021 (**A**) and RX528.2021 **(B)** probands. **(C)** Lymphoscintigraphy (anterior view) of the lower extremities of RX626.2021. Images were taken at 0, 30, 60 and 120 minutes after injection. Lymphoscintigraphy (tracer: 99m-Tc-nanocolloid) was performed by intradermal and subcutaneous infiltration of radionuclides into the interdigital spaces of both feet (tracer depot is visible at the site of the injection). Pelvis, knee and feet are indicated as anatomical reference. Late imaging at 120 minutes shows normal visualization of inguinal and pelvic lymph node stations on the left. On the right, inguinal lymph nodes are less represented (arrow with I). Popliteal lymph nodes are detected (arrow with K), likely due to rerouting of the tracer via the deep lymphatic system. Lateroiliac stations are more pronounced on the right than on the left (arrow with L). Lymphoscintigraphy evidences marked reduction of lymphatic circulation on the right with mild lateroiliac recovery, and mild impairment of lateroiliac transit on the left. **(D)** Lymphoscintigraphy (anterior view) of the lower extremities of RX528.2021. Images were taken at 30- and 90-min post-injection. Lymphoscintigraphy (tracer: 99m-Tc-nanocolloid) was performed by intradermal and subcutaneous infiltration of radionuclides into the interdigital spaces of both feet (tracer depot is visible at the site of the injection). Pelvis, knee and feet are indicated as anatomical reference. Although quantification of the tracer was not available from the hospital, late phase imaging at 90 minutes reveals potential mild reduction in bilateral inguinal uptake on the right (arrow with I), consistent with the patient's clinical presentation of bilateral lower limb lymphedema with R>L. Please note that lymphoscintigraphy was conducted in two different Centers in Italy by different clinicians, thus the differences in imaging times. **(E)** Clinical data of the RX626.2021 and RX528.2021 probands.

figure S19

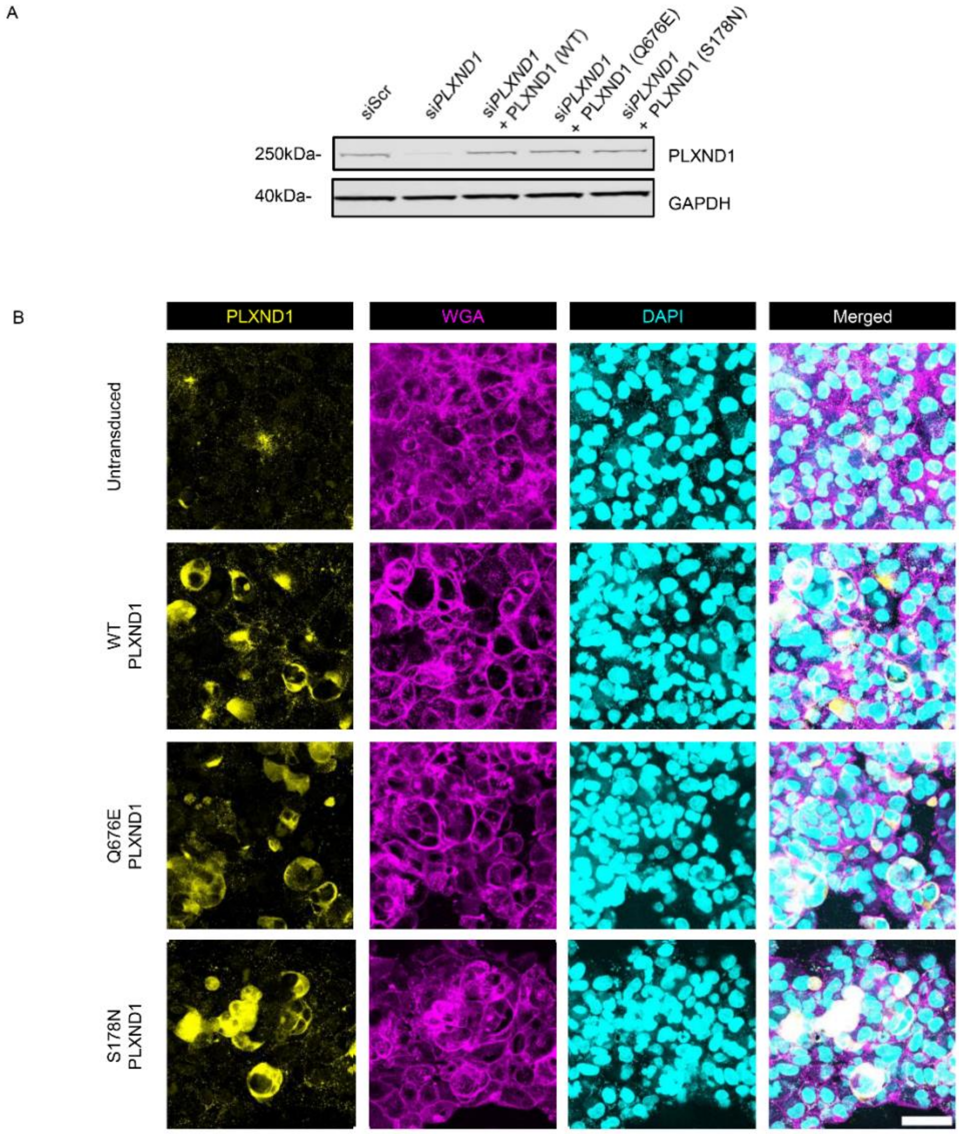

Fig. S19

Expression and subcellular localization of PLXND1 mutants. (A) HLECs transfected with either scramble or *PLXND1* siRNAs were transduced with adenoviruses expressing WT or mutant Q676E PLXND1. Western blots show PLXND1 expression and GAPDH as loading control. *n*=3 per condition. **(B)** Cos7 cells transduced with adenoviruses expressing WT or mutant Q676E or S178N PLXND1 (or untransduced controls) were fixed (without permeabilization) and immunostained for PLXND1 (yellow), wheat germ agglutinin (WGA) (magenta), and DAPI (cyan). *n*=3 per condition; *n*>30 cells analyzed. Scale bar: 50µm.

figure S20

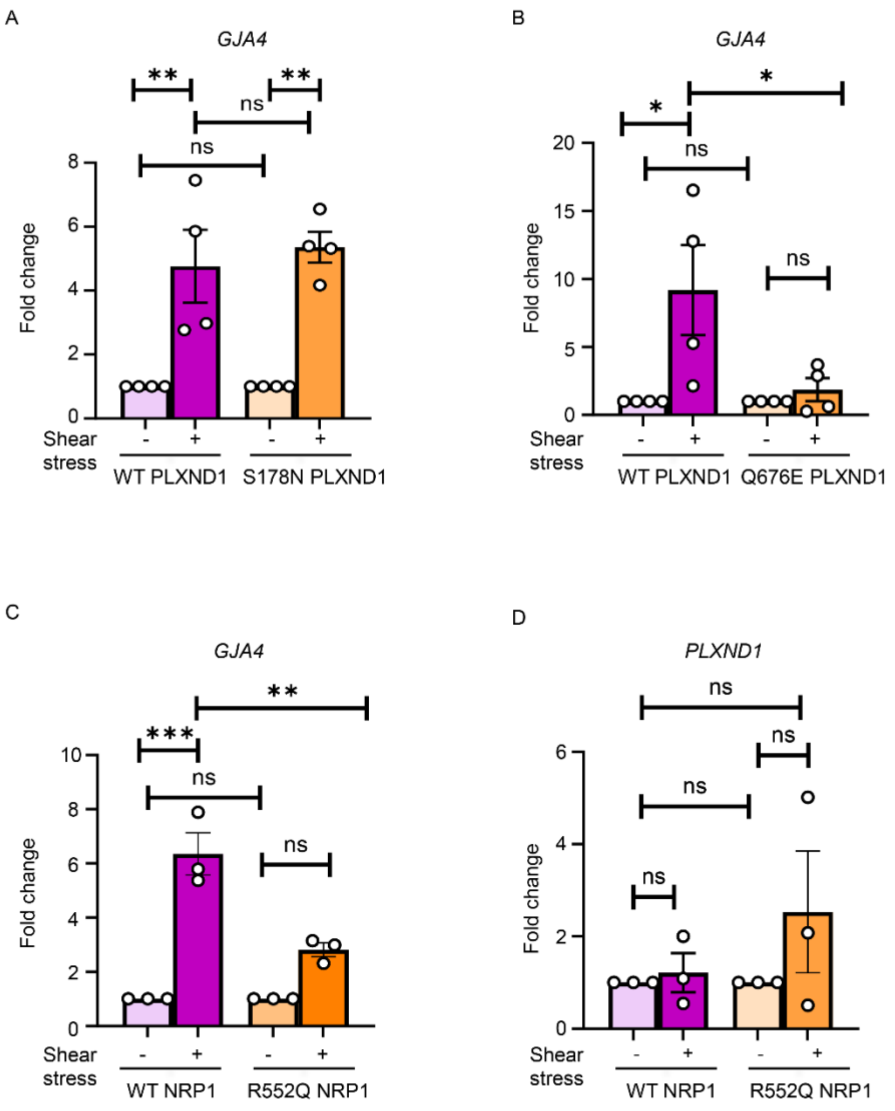

Fig. S20

Effect of PLXND1 and NRP1 mutations on *GJA4* expression under shear stress. (A) Following siRNA-mediated depletion of endogenous *PLXND1*, HLECs were transduced with adenoviruses expressing either WT or mutant S178N *PLXND1* and exposed to shear stress for 24 h. *GJA4* mRNA levels were assessed by qPCR. $n = 4$ biological replicates; two-way ANOVA; Tukey's multiple comparisons test, $**p < 0.01$) **(B)** Following siRNA-mediated depletion of endogenous *PLXND1*, HLECs were transduced with adenoviruses expressing either WT or mutant Q676E *PLXND1* and exposed to shear stress for 24 h. *GJA4* mRNA levels were assessed by qPCR. $n = 4$ biological replicates; two-way ANOVA; Tukey's multiple comparisons test, $*p < 0.05$. **(C)** Following siRNA-mediated depletion of endogenous *NRP1*, HLECs were transduced with adenoviruses expressing either WT or mutant R552Q *NRP1* and exposed to shear stress for 24 h. *GJA4* mRNA levels were assessed by qPCR. ($n = 3$ biological replicates; two-way ANOVA; Tukey's multiple comparisons test, $**p < 0.01$, $***p < 0.001$). **(D)** Following siRNA-mediated depletion of endogenous *NRP1*, HLECs were transduced with adenoviruses expressing either WT or mutant R552Q *NRP1* and exposed to shear stress for 24 h. *PLXND1* mRNA levels were assessed by qPCR. $n = 3$ biological replicates; two-way ANOVA; Tukey's multiple comparisons test.

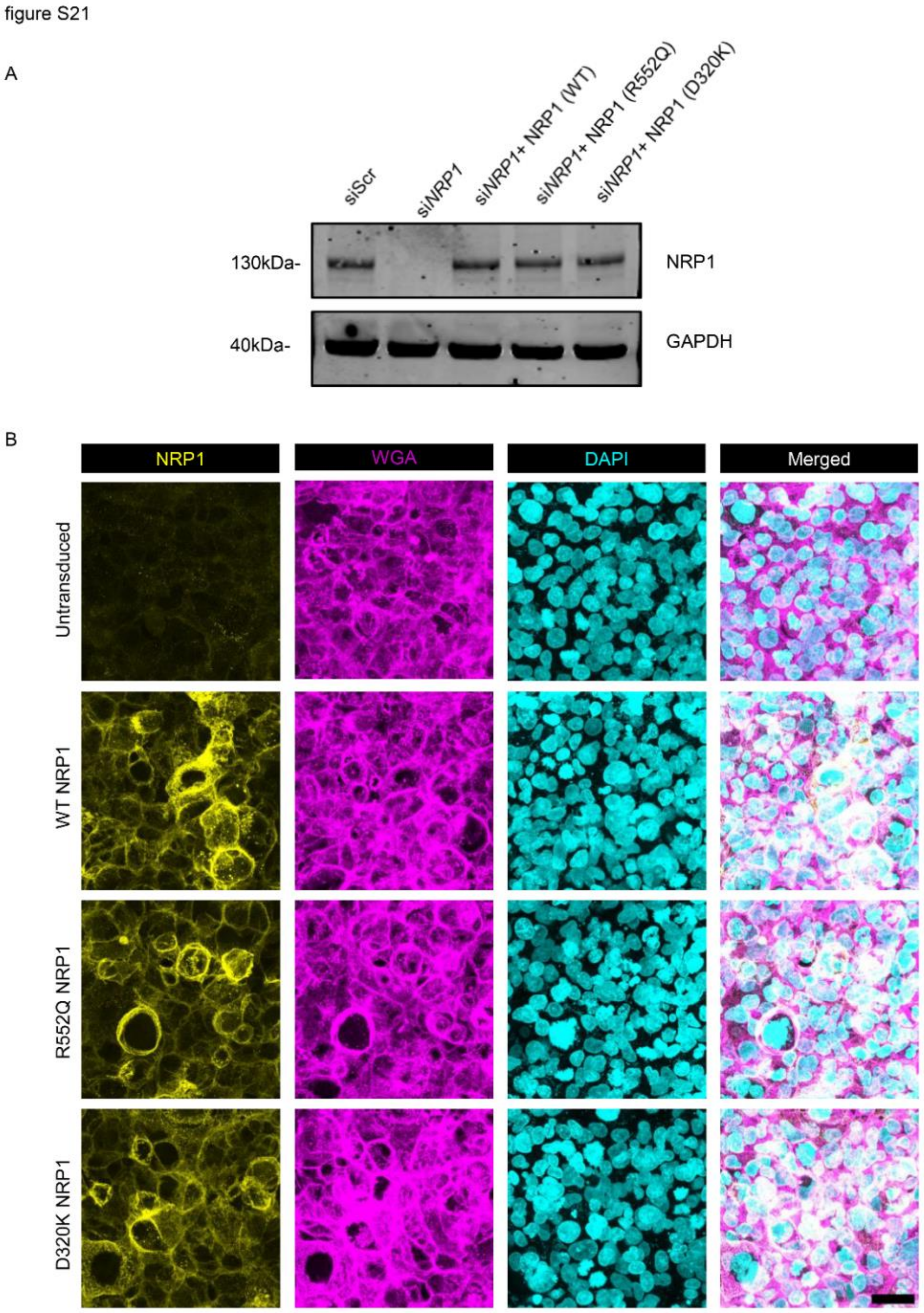

Fig. S21

Expression and subcellular localization of NRP1 mutants (A) HLECs transfected with either scramble or *NRP1* siRNAs were transduced with adenoviruses expressing WT, R552Q or D320K NRP1. Western blots show NRP1 expression and GAPDH as loading control. *n* = 3 biological replicates/per condition **(B)** Cos7 cells transduced with adenoviruses expressing WT, R552Q or D320K NRP1 (or untransduced controls) were fixed (without permeabilization) and immunostained for NRP1 (yellow), wheat germ agglutinin (WGA) (magenta), and DAPI (cyan). *n* = 3 per condition; n>30 cells analyzed. Scale bar: 50µm.

figure S22

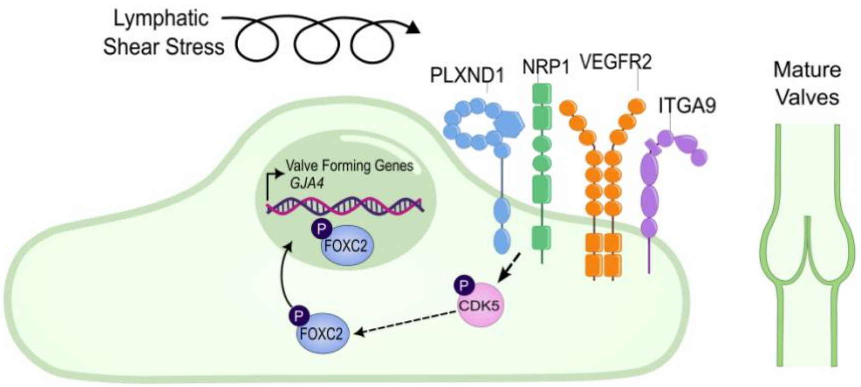

Fig. S22
Schematic of proposed model. In response to shear stress, a PLXND1-based lymphatic mechano-complex forms (comprised of PLXND1, NRP1, VEGFR2, and ITGA9) which regulates activation of CDK5, phosphorylation of FOXC2, and downstream *GJA4* upregulation. This pathway is essential for lymphatic valve formation.

A

Variant	Variant	Coordinates (GRCh38)	Allele	Reference Allele	Exon	cDNA_ position	CDS_ position	Protein_ position	Amino_ acids	Codons	Existing_ variation
1	ENSP00000317128.4: p.Ser178Asn	3:129606107	T	G	1/36	570	533	178	S/N	aGc/aAc	-
2	ENSP00000317128.4: p.Ser356Ile	3:129605573	A	G	1/36	1104	1067	356	S/I	aGc/aTc	rs753099478
3	ENSP00000317128.4: p.Ala564Thr	3:129586203	T	G	4/36	1727	1690	564	A/T	Gcc/Acc	rs373023998,COSV60713636
4	ENSP00000317128.4: p.Val612Met	3:129585969	T	G	5/36	1871	1834	612	V/M	Gtg/Atg	rs766394256
5	ENSP00000317128.4: p.Gln676Glu	3:129584388	C	C	6/36	2063	2026	676	Q/E	Cag/Gag	rs1248086143
6	ENSP00000317128.4: p.Gly931Val	3:129573639	A	G	13/36	2829	2792	931	G/V	gGt/gTt	rs780775472
7	ENSP00000317128.4: p.Leu1041Val	3:129571801	C	C	16/36	3158	3121	1041	L/V	Ctg/Gtg	rs150425885
8	ENSP00000317128.4: p.Arg1633Leu	3:129561831	A	G	28/36	4935	4898	1633	R/L	cGc/cTc	rs769768899,COSV60711933
9	ENSP00000317128.4: p.Asp1757Asn	3:129559648	T	G	32/36	5306	5269	1757	D/N	Gac/Aac	rs781629375,COSV60712847

B

Variant	Variant	SIFT	PolyPhen	gnomADe_ AF	gnomADg_ AF	REVEL	CADD_ PHRED	am_class	am_ pathogenicity
1	ENSP00000317128.4: p.Ser178Asn	deleterious_low_ confidence(0)	probably_ Damaging (0.996)	-	-	0.183	28.2	likely_ pathogenic	0.9637
2	ENSP00000317128.4: p.Ser356Ile	deleterious_low_ confidence(0)	benign(0.37)	6.51E-06	-	0.197	25.1	ambiguous	0.4737
3	ENSP00000317128.4: p.Ala564Thr	deleterious_low_ confidence(0.02)	probably_ Damaging (0.987)	4.13E-06	6.57E-06	0.255	25	ambiguous	0.3513
4	ENSP00000317128.4: p.Val612Met	deleterious_low_ confidence(0.05)	benign(0.104)	3.42E-06	-	0.022	19.13	likely_benign	0.1012
5	ENSP00000317128.4: p.Gln676Glu	deleterious_low_ confidence(0.04)	benign(0.092)	1.44E-05	6.57E-06	0.09	21.2	likely_benign	0.08
6	ENSP00000317128.4: p.Gly931Val	tolerated_low_ confidence(0.12)	benign(0.152)	0.0001163	2.63E-05	0.303	12.18	likely_benign	0.1738
7	ENSP00000317128.4: p.Leu1041Val	tolerated_low_ confidence(0.19)	possibly_ Damaging (0.566)	0.0007756	0.0003877	0.104	15.11	likely_benign	0.0733
8	ENSP00000317128.4: p.Arg1633Leu	deleterious_low_ confidence(0)	probably_ Damaging (0.969)	8.90E-06	6.57E-06	0.141	27.2	ambiguous	0.5024
9	ENSP00000317128.4: p.Asp1757Asn	deleterious_low_ confidence(0)	probably_ Damaging (0.999)	3.09E-05	1.97E-05	0.352	31	likely_benign	0.31

Table S4

***PLXND1* variants in primary lymphedema patients identified by exome and genome sequencing.** Tables showing the nine *PLXND1* variants in primary lymphedema patients identified by exome and genome sequencing. All nine variants are missense, and the impacts reported are for the canonical transcript (ENST00000324093.9). **(A)** Details on genomic location and predicted amino acid change. **(B)** *In-silico* predictions for variants in *PLXND1*, including SIFT, PolyPhen, REVEL, CADD and AlphaMissense (am). GnomAD exome (gnomADe_AF) and genome (gnomADg_AF) global allele frequencies are reported. Annotations produced by VEP, Ensembl release 113.

References

1. Sabine A, Agalarov Y, Maby-El Hajjami H, Jaquet M, Hagerling R, Pollmann C, et al. Mechanotransduction, PROX1, and FOXC2 cooperate to control connexin37 and calcineurin during lymphatic-valve formation. *Dev Cell.* 2012;22(2):430-45.
2. Zawieja DC. Contractile physiology of lymphatics. *Lymphat Res Biol.* 2009;7(2):87-96.
3. Mehta V, Pang KL, Rozbesky D, Nather K, Keen A, Lachowski D, et al. The guidance receptor plexin D1 is a mechanosensor in endothelial cells. *Nature.* 2020;578(7794):290-5.
4. Saygili Demir C, Sabine A, Gong M, Dormond O, and Petrova TV. Mechanosensitive mTORC1 signaling maintains lymphatic valves. *J Cell Biol.* 2023;222(6).
5. Nonomura K, Lukacs V, Sweet DT, Goddard LM, Kanie A, Whitwam T, et al. Mechanically activated ion channel PIEZO1 is required for lymphatic valve formation. *Proc Natl Acad Sci U S A.* 2018;115(50):12817-22.
6. Aricescu AR, Lu W, and Jones EY. A time- and cost-efficient system for high-level protein production in mammalian cells. *Acta Crystallogr D Biol Crystallogr.* 2006;62(Pt 10):1243-50.
7. Michelini S, Degiorgio D, Cestari M, Corda D, Ricci M, Cardone M, et al. Clinical and genetic study of 46 Italian patients with primary lymphedema. *Lymphology.* 2012;45(1):3-12.
8. Michelini S, Vettori A, Maltese PE, Cardone M, Bruson A, Fiorentino A, et al. Genetic Screening in a Large Cohort of Italian Patients Affected by Primary Lymphedema Using a Next Generation Sequencing (NGS) Approach. *Lymphology.* 2016;49(2):57-72.
9. Meghnani V, Mohammed N, Giauque C, Nahire R, and David T. Performance Characterization and Validation of Saliva as an Alternative Specimen Source for Detecting Hereditary Breast Cancer Mutations by Next Generation Sequencing. *Int J Genomics.* 2016;2016.
10. Bonetti G, Paolacci S, Samaja M, Maltese PE, Michelini S, Michelini S, et al. Low Efficacy of Genetic Tests for the Diagnosis of Primary Lymphedema Prompts Novel Insights into the Underlying Molecular Pathways. *Int J Mol Sci.* 2022;23(13).
11. Gnirke A, Melnikov A, Maguire J, Rogov P, LeProust EM, Brockman W, et al. Solution hybrid selection with ultra-long oligonucleotides for massively parallel targeted sequencing. *Nat Biotechnol.* 2009;27(2):182-9.
12. Richards S, Aziz N, Bale S, Bick D, Das S, Gastier-Foster J, et al. Standards and guidelines for the interpretation of sequence variants: a joint consensus recommendation of the American College of Medical Genetics and Genomics and the Association for Molecular Pathology. *Genet Med.* 2015;17(5):405-24.
13. ClinVar. https://www.ncbi.nlm.nih.gov/clinvar/ https://www.ncbi.nlm.nih.gov/clinvar/ Updated 21/03/2024.
14. Sherry ST, Ward MH, Kholodov M, Baker J, Phan L, Smigielski EM, et al. dbSNP: the NCBI database of genetic variation. *Nucleic Acids Res.* 2001;29(1):308-11.
15. Kopanos C, Tsiolkas V, Kouris A, Chapple CE, Albarca Aguilera M, Meyer R, et al. VarSome: the human genomic variant search engine. *Bioinformatics.* 2019;35(11):1978-80.
16. . gnomAD. Genome Aggregation Database. Accessed Feb. 01, 2024.
17. Marceddu G, Dallavilla T, Guerri G, Zulian A, Marinelli C, and Bertelli M. Analysis of machine learning algorithms as integrative tools for validation of next generation sequencing data. *Eur Rev Med Pharmaco.* 2019;23(18):8139-47.
18. Cristofoli F, Daja M, Maltese PE, Guerri G, Tanzi B, Miotto R, et al. MAGI-ACMG: Algorithm for the Classification of Variants According to ACMG and ACGS Recommendations. *Genes (Basel).* 2023;14(8).
19. Caulfield M, Davies J, Dennys M, Elbahy L, Fowler T, Hill S, et al.: https://ndownloader.figshare.com/files/22714349; 2020.
