# Supplementary material for: Mechanoreceptor plexin D1 regulates lymphatic valve morphogenesis and lymphedema pathogenesis
Source: J Clin Invest. 2026 Jul 1;136(13):e193385. doi: 10.1172/JCI193385 (PMC13318119; doi:10.1172/JCI193385)

# Figure 2D

pSerine

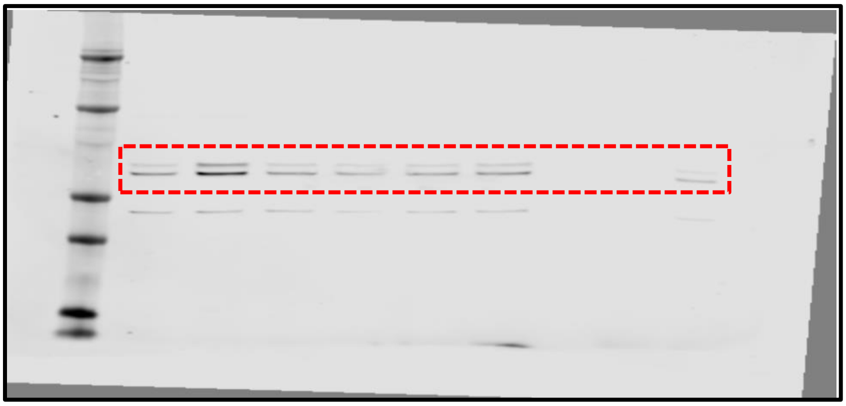

FOXC2

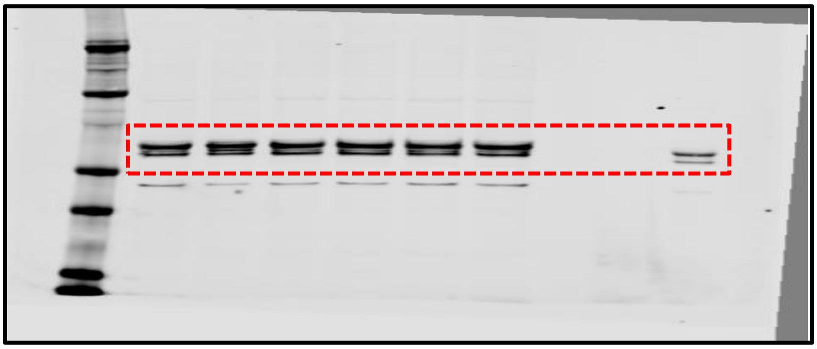

# Figure 2E

pCDK5

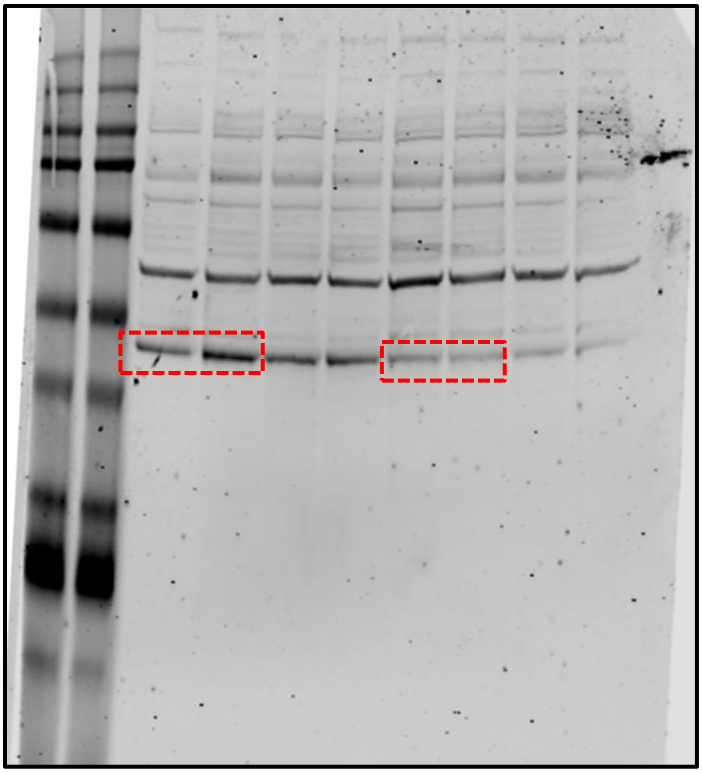

CDK5

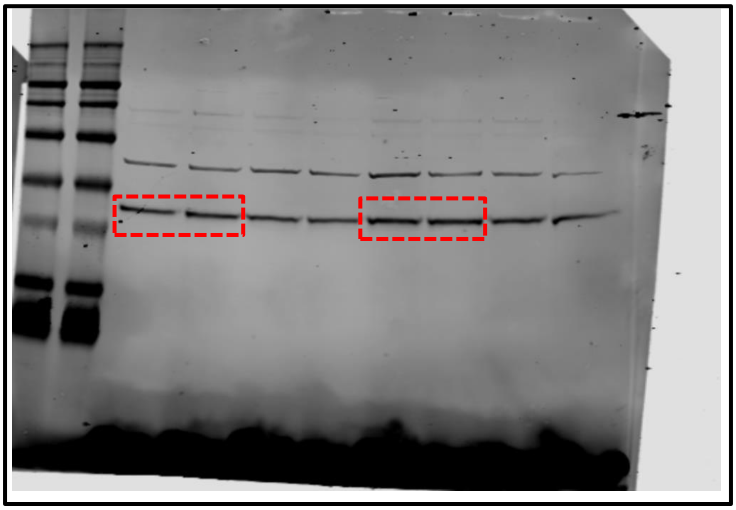

# Figure 3D

pCDK5

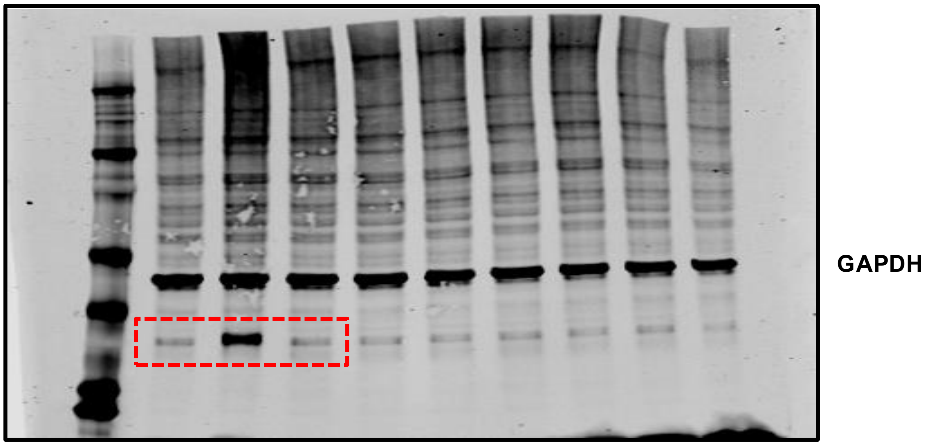


CDK5

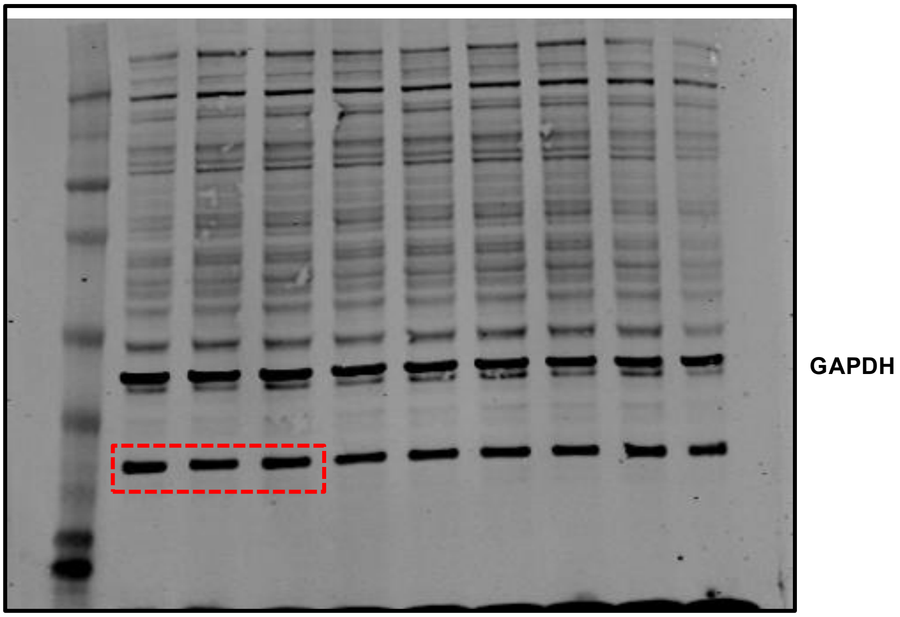


GAPDH

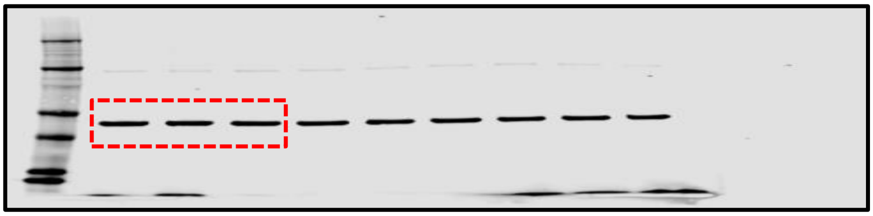

GAPDH was probed on the same membrane as phospho-CDK5 but is shown at a different exposure for clarity

## Figure 3E

pCDK5

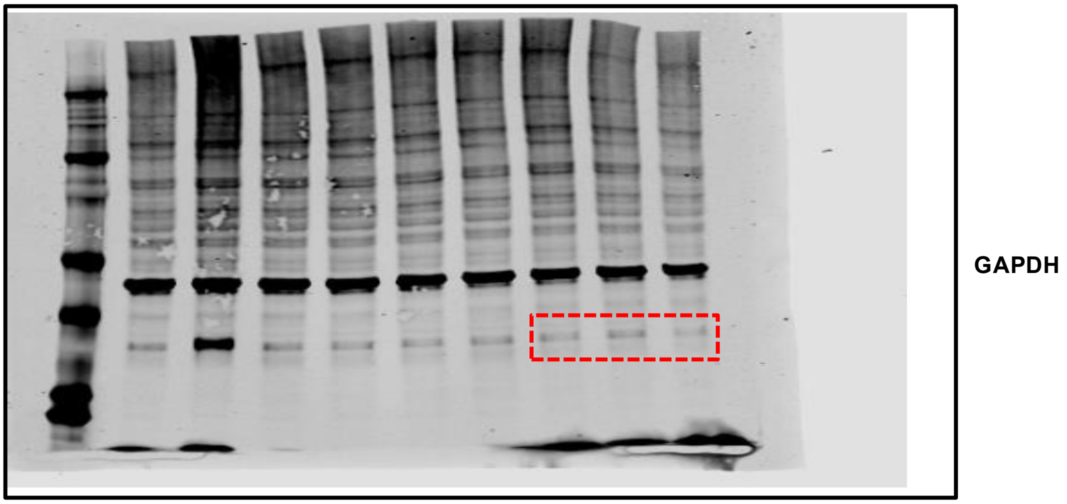


CDK5

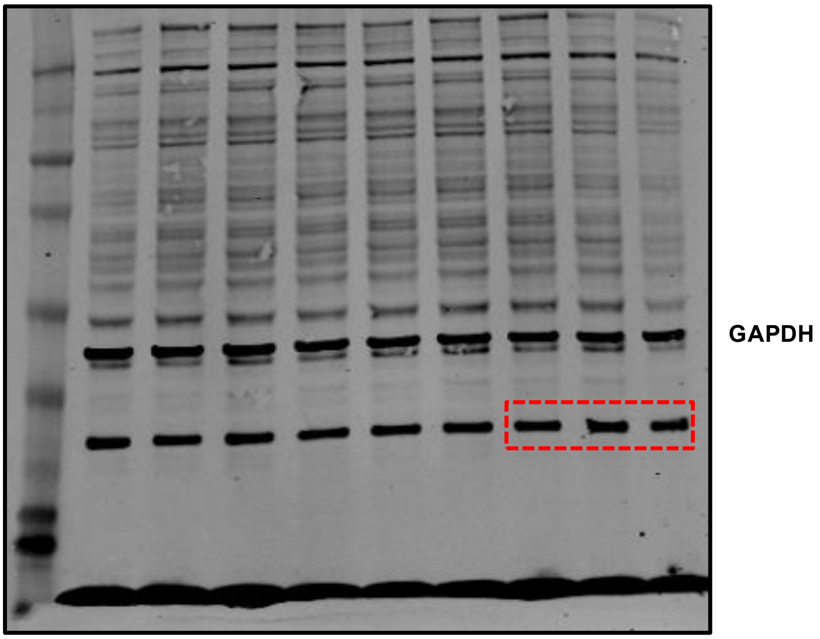


GAPDH

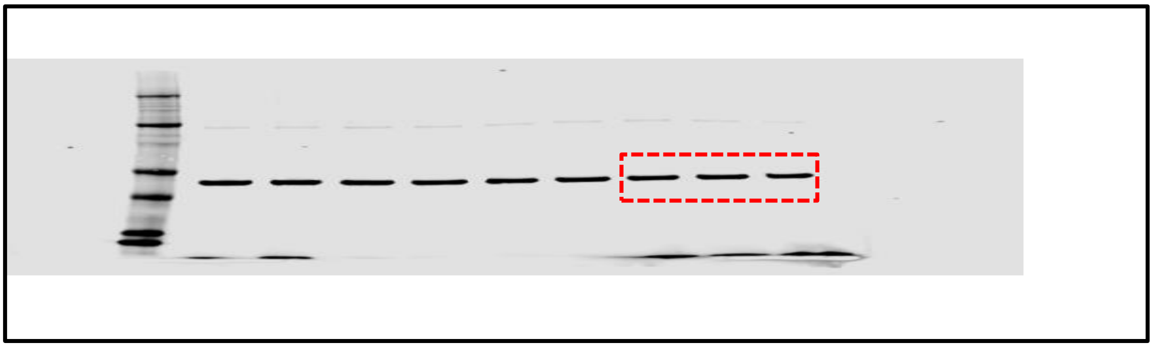

# Figure 3F

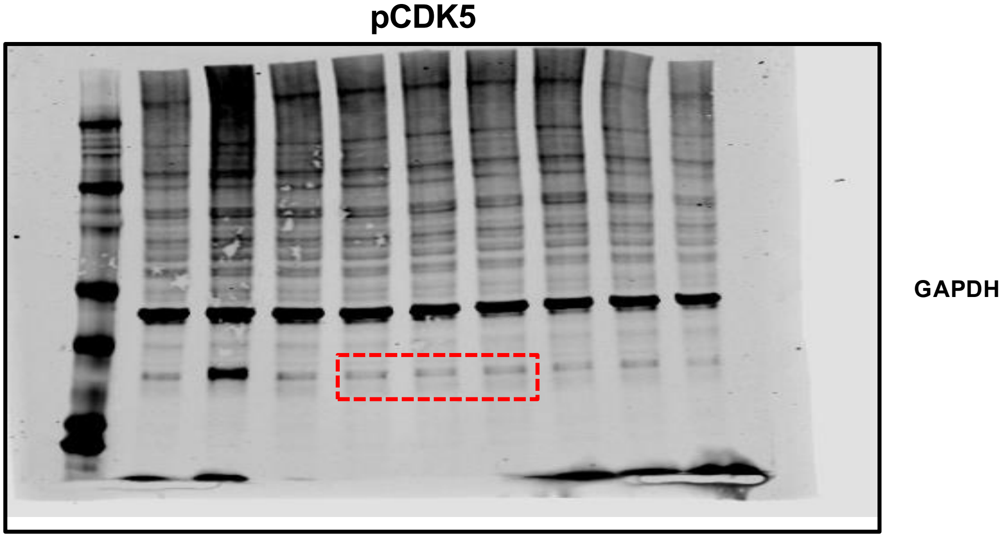


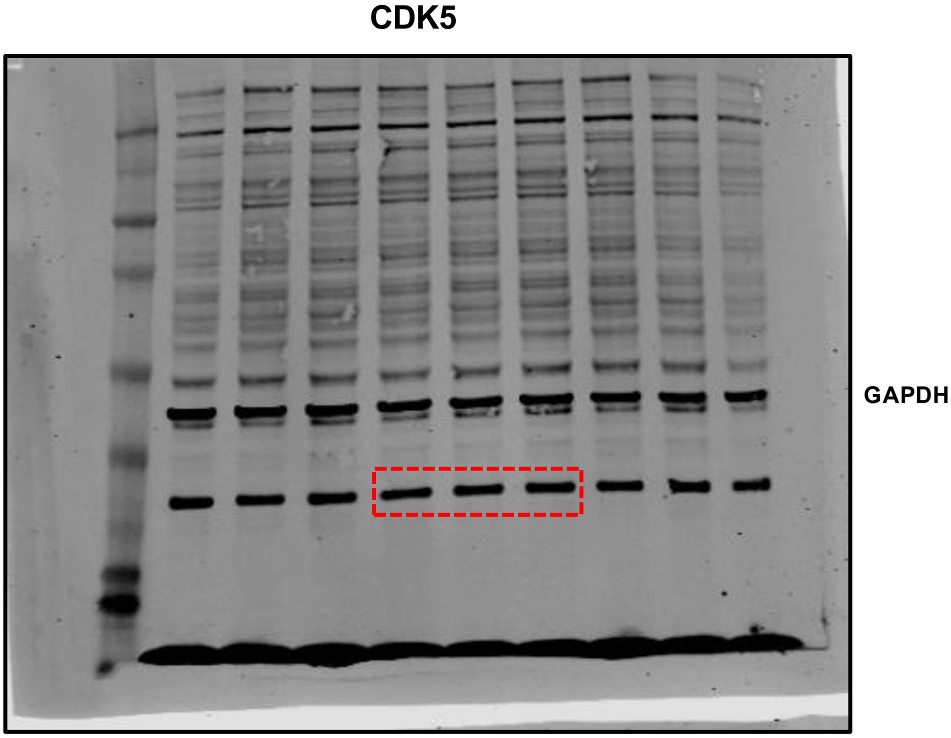


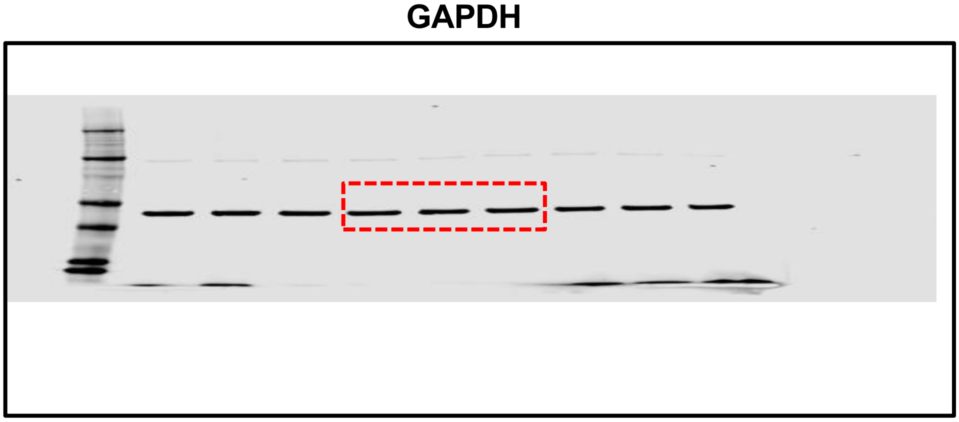

## Figure 3G

pERK1/2

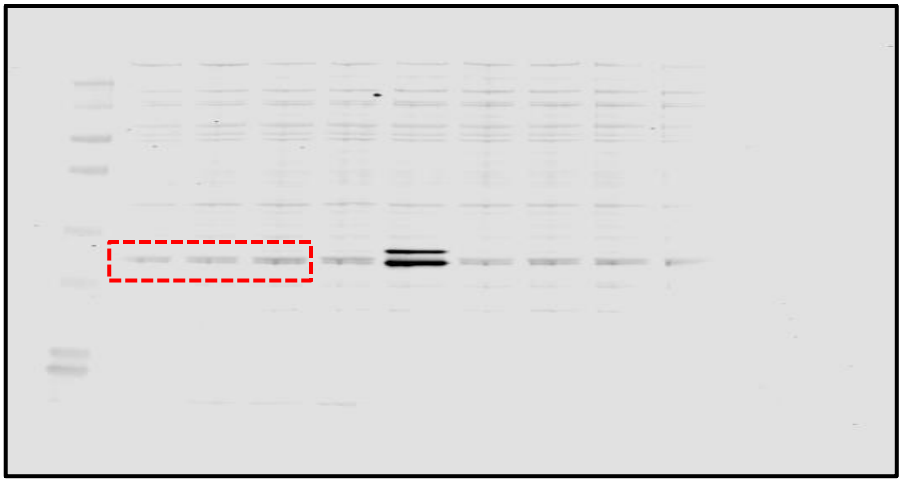

ERK1/2

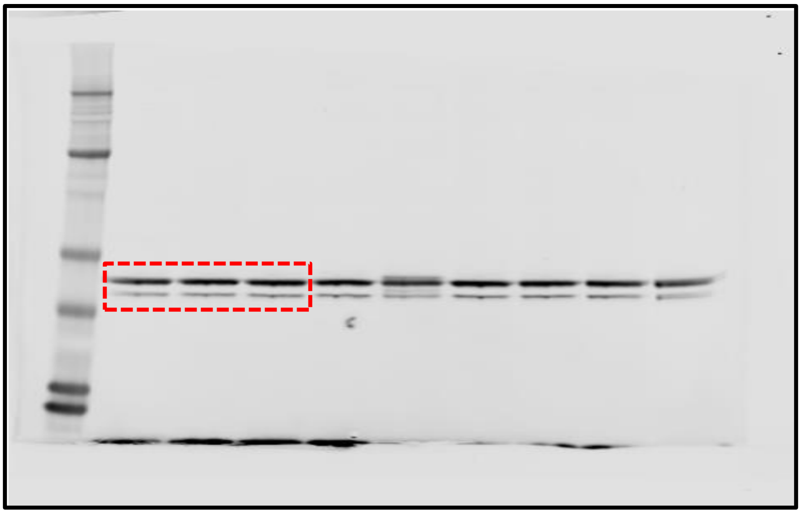

## Figure 3H

pERK1/2

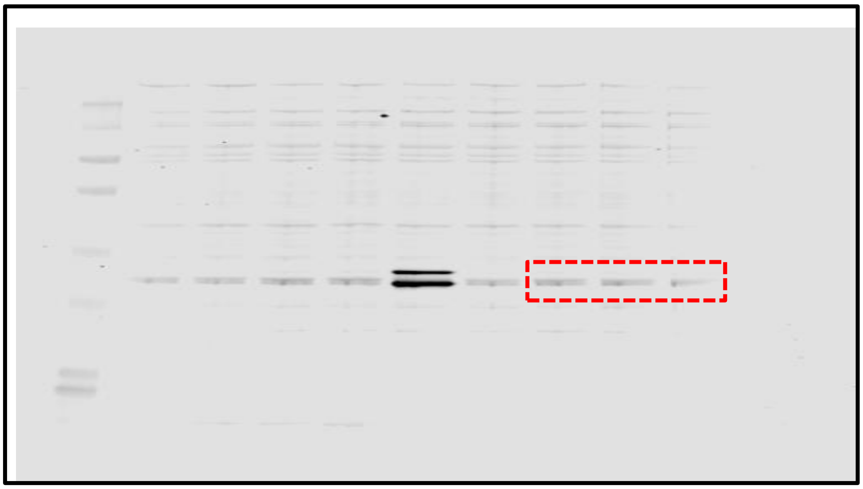

ERK1/2

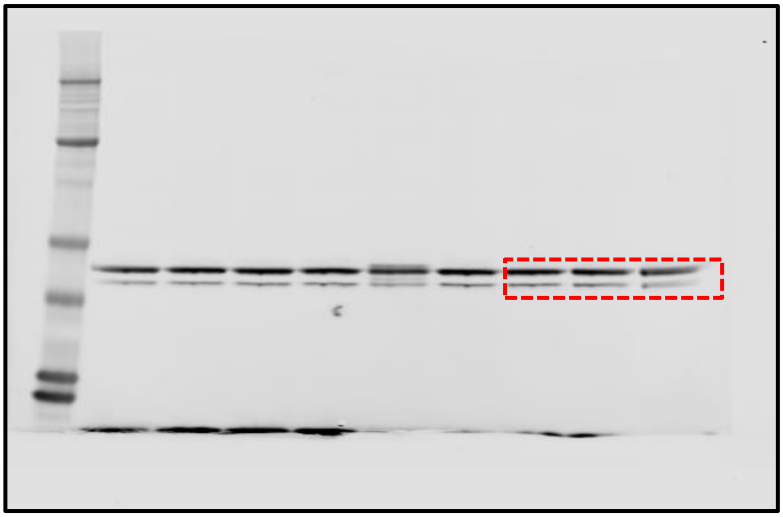

## Figure 3I

pERK1/2

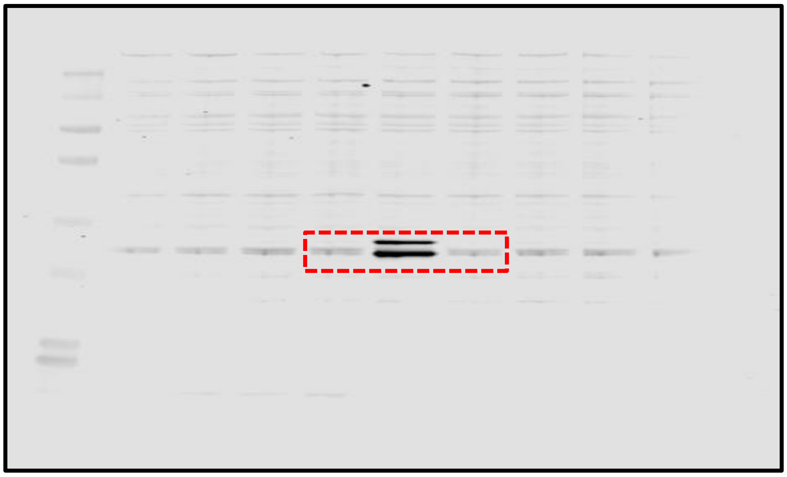

ERK1/2

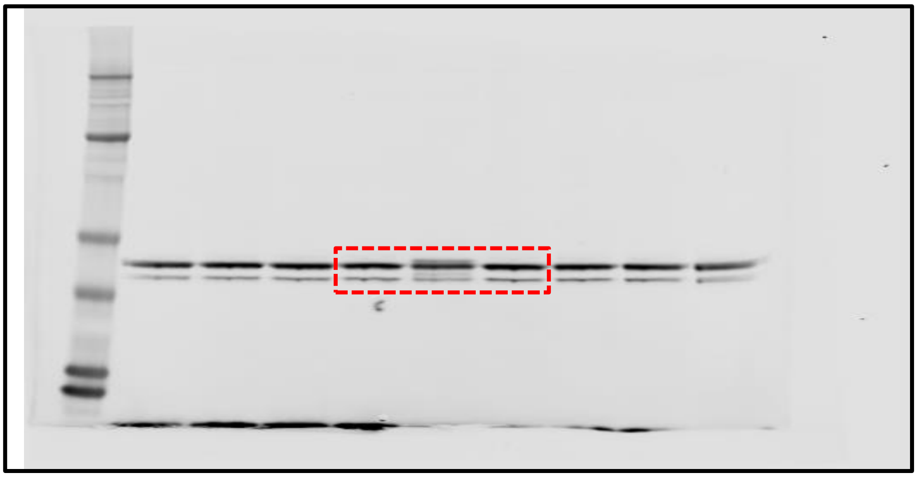

## Figure 4B

VEGFR2

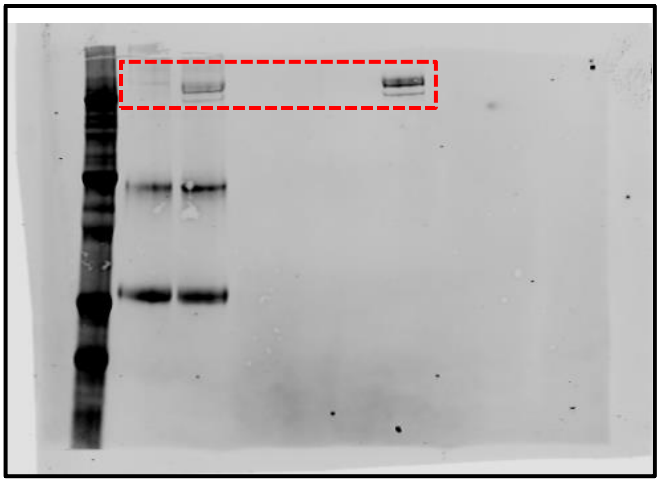

NRP1

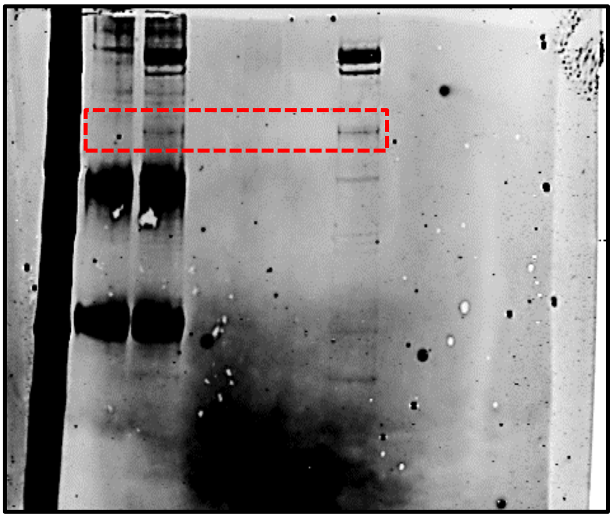

CDK5

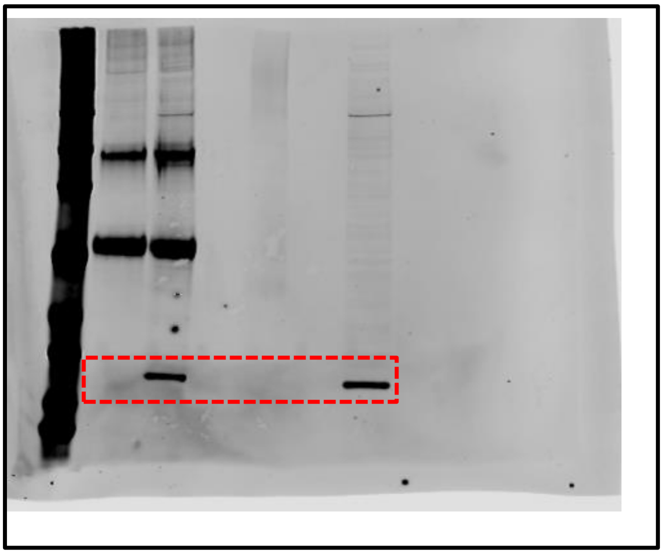

## Figure 4B

ITGA9

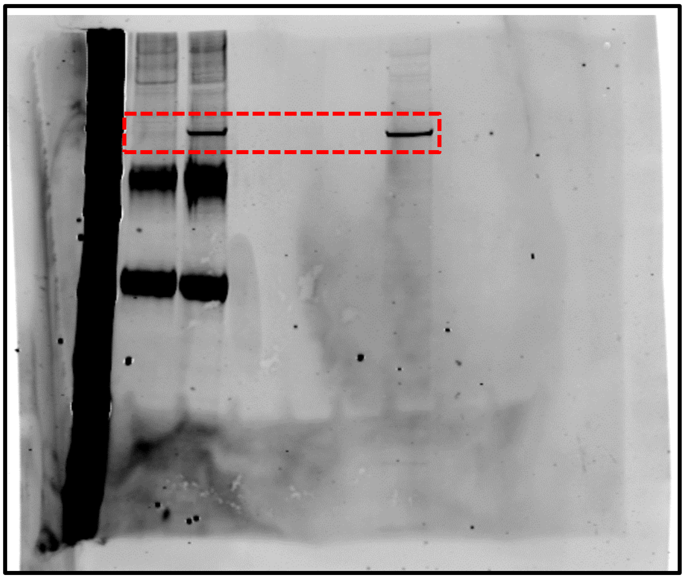

PLXND1

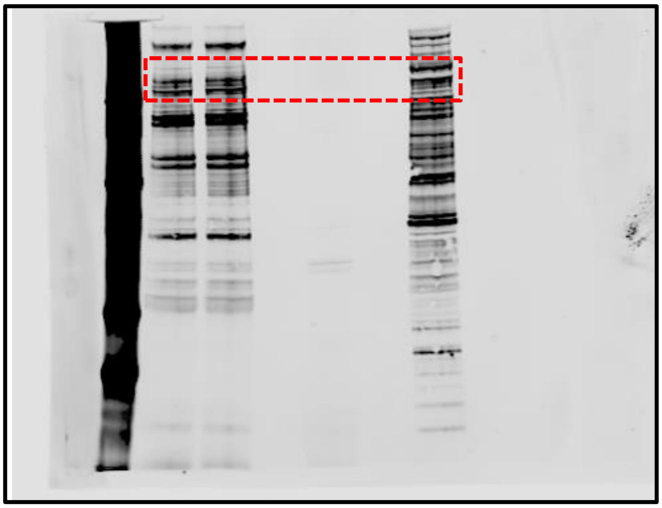

# Figure 4C

PLXND1

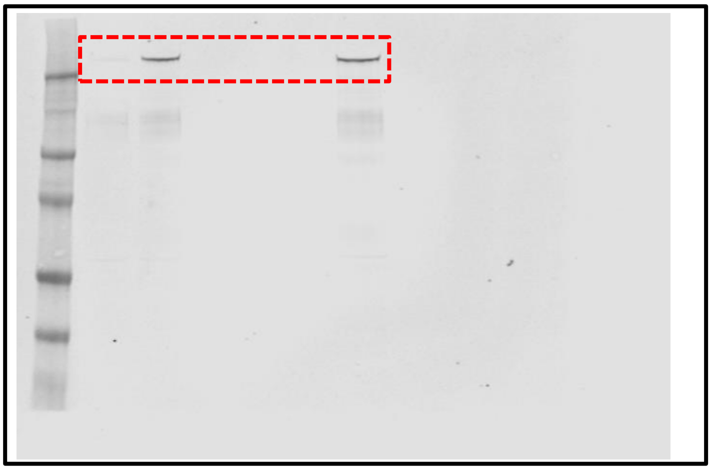

NRP1

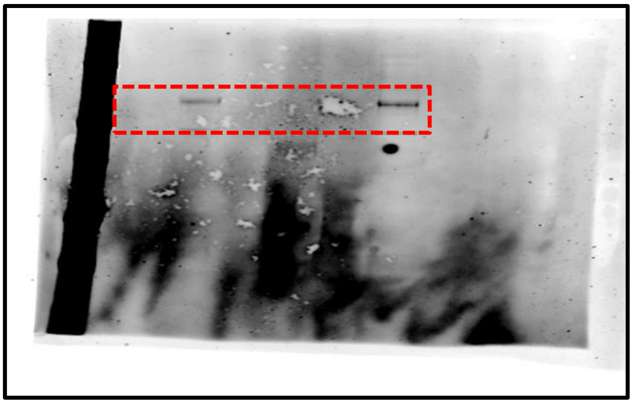

CDK5

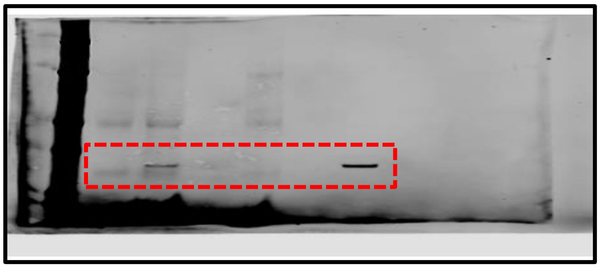

## Figure 4C

ITGA9

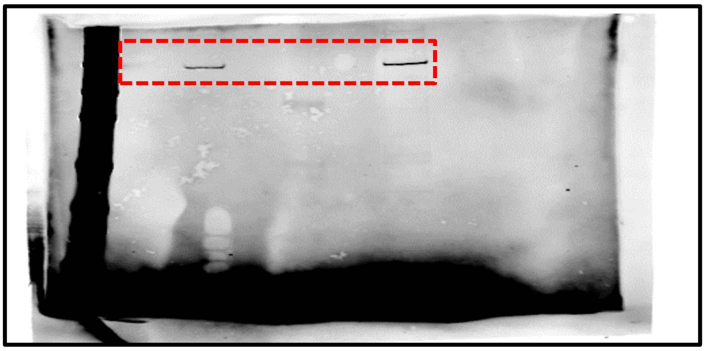

VEGFR2

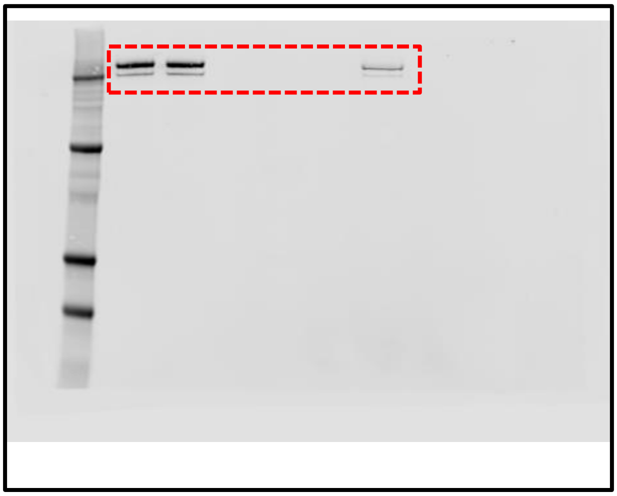

## Figure 4D

PLXND1

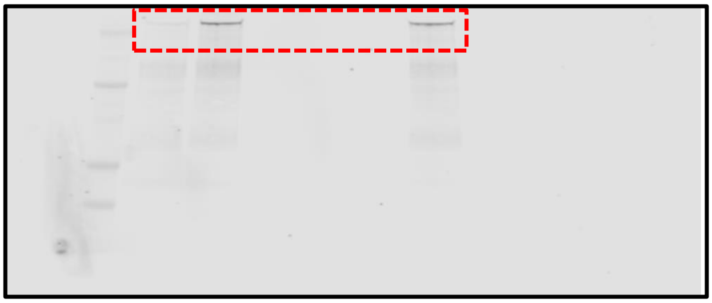

VEGFR2

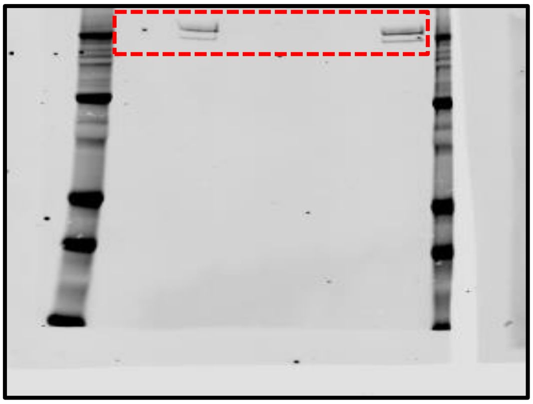

ITGA9

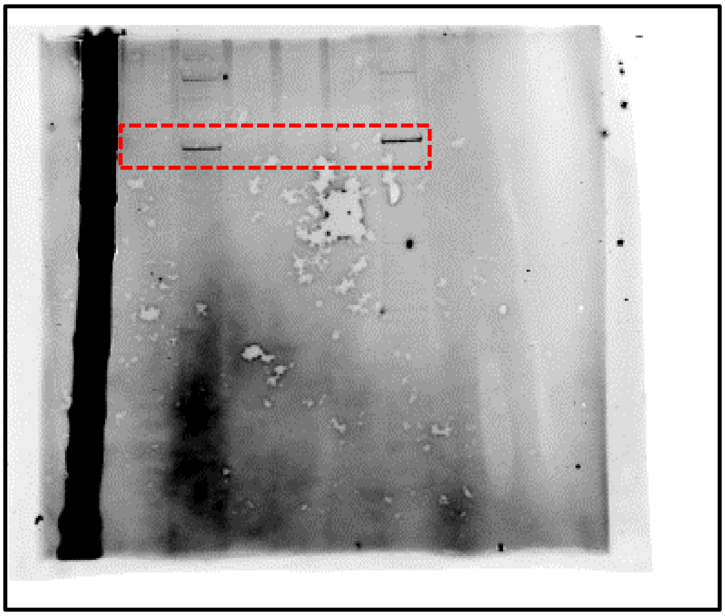

# Figure 4D

NRP1

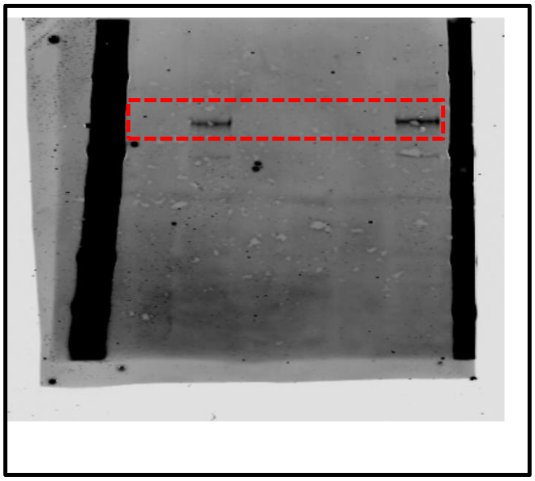

CDK5

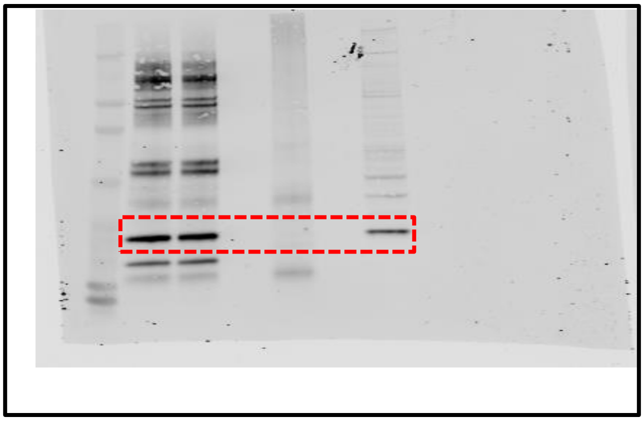

## Figure 5D

pCofilin

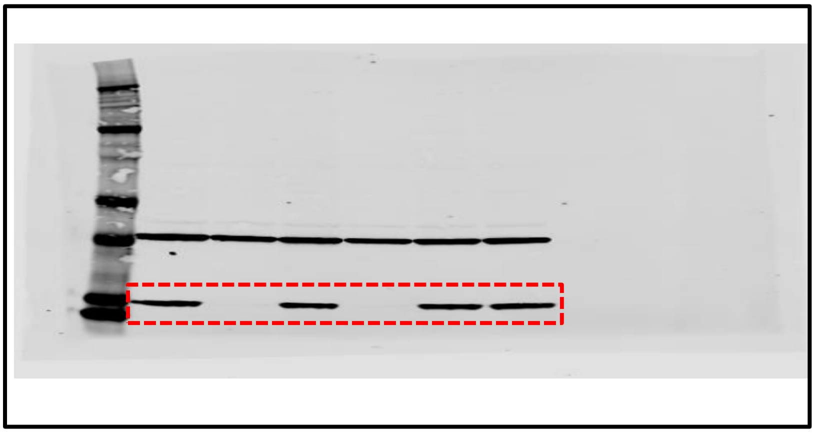


Cofilin

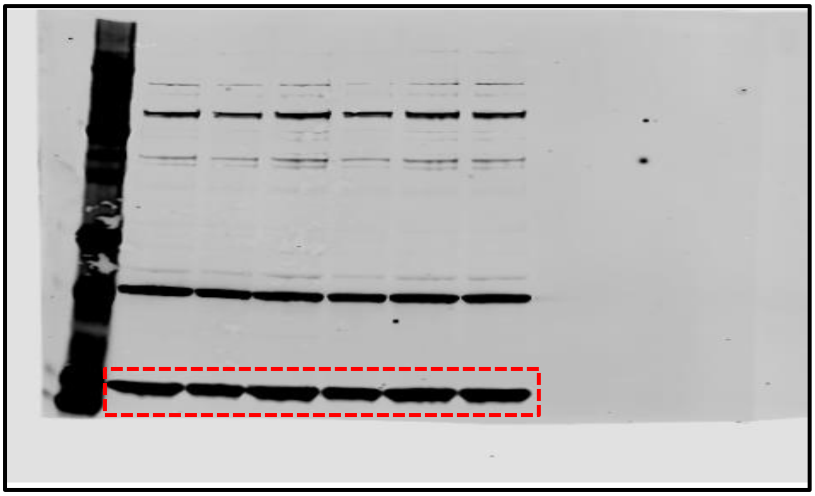


pFAK

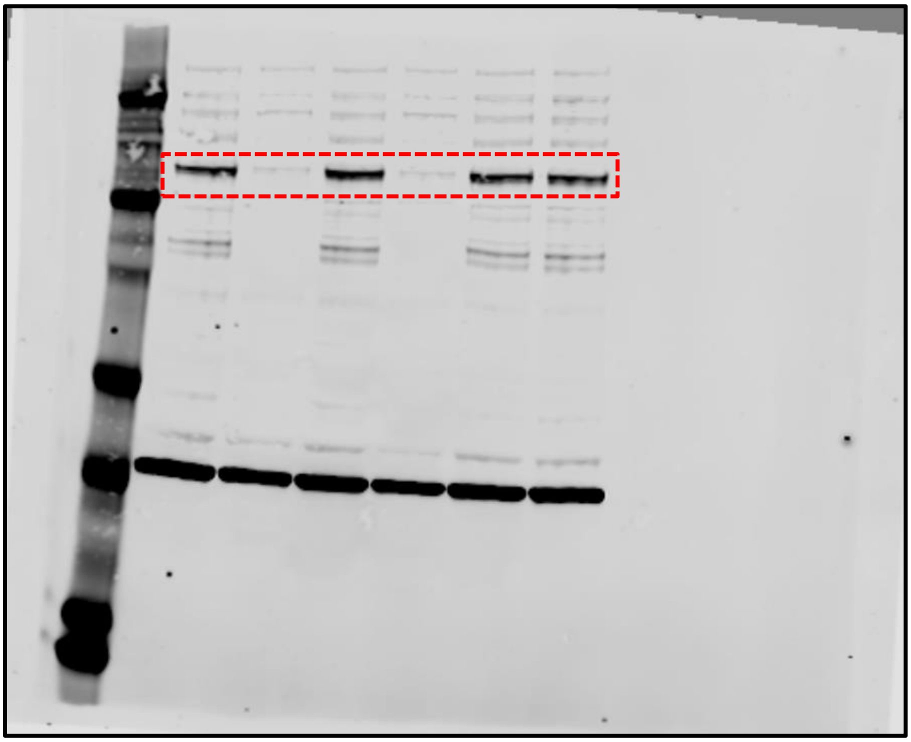

# Figure 5D

FAK

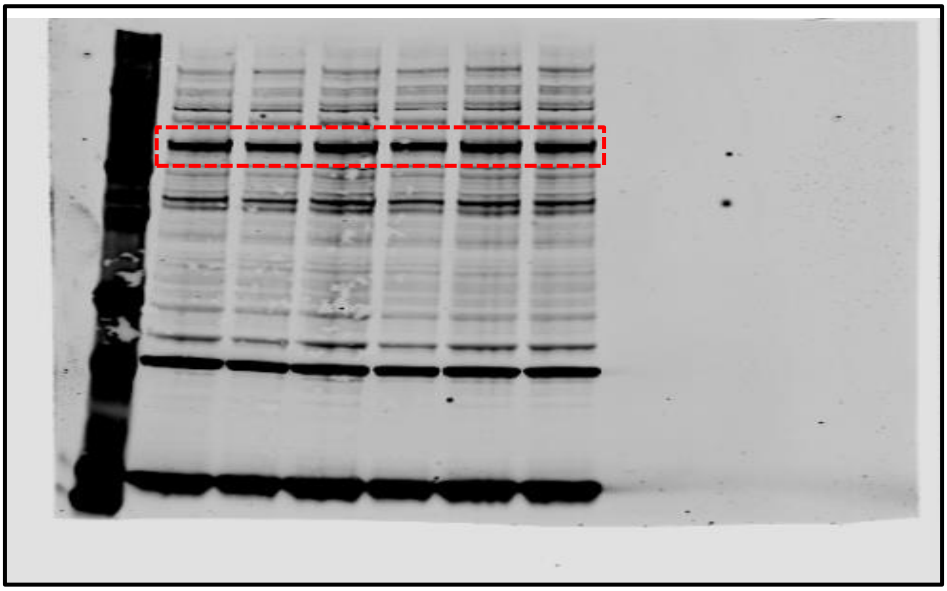

GAPDH

GAPDH

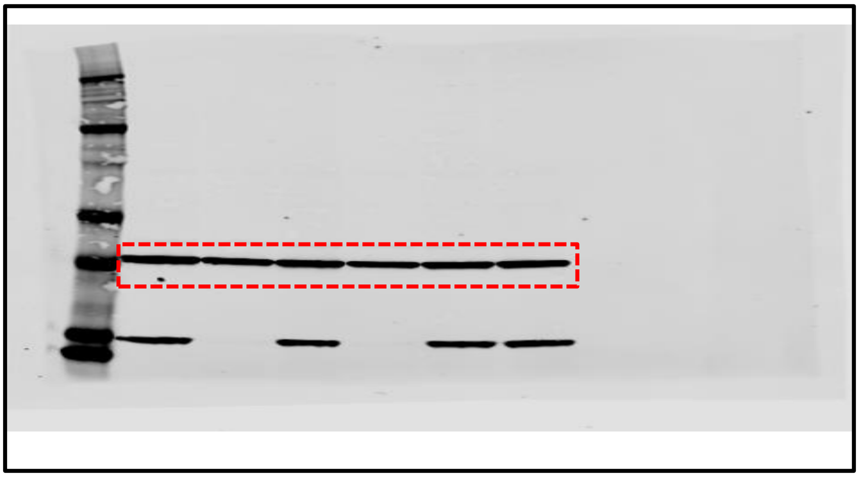

# Figure 5F

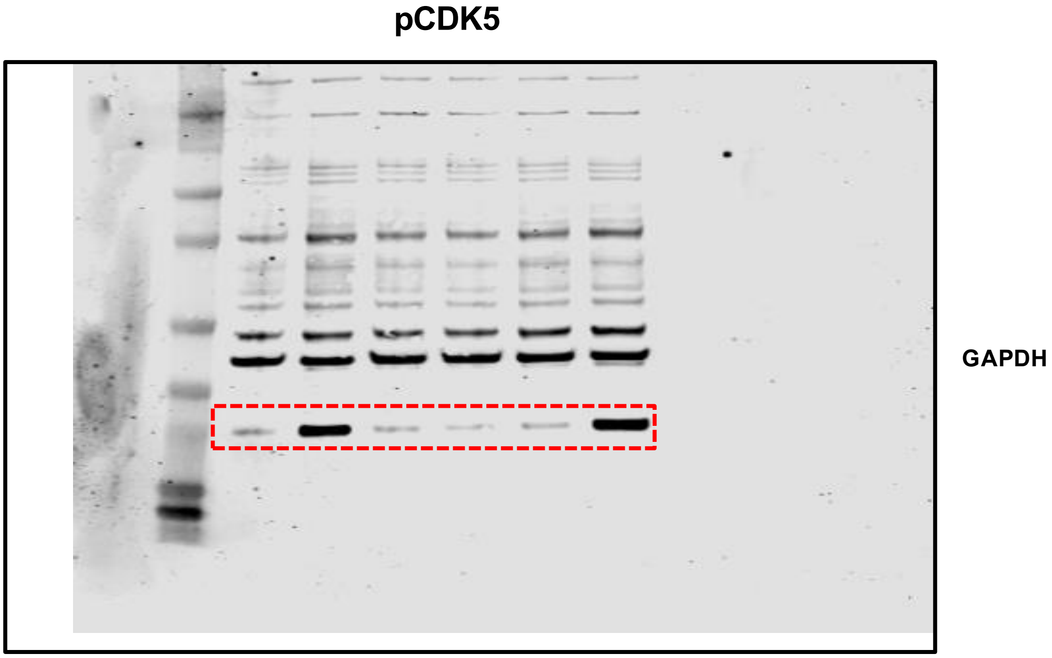


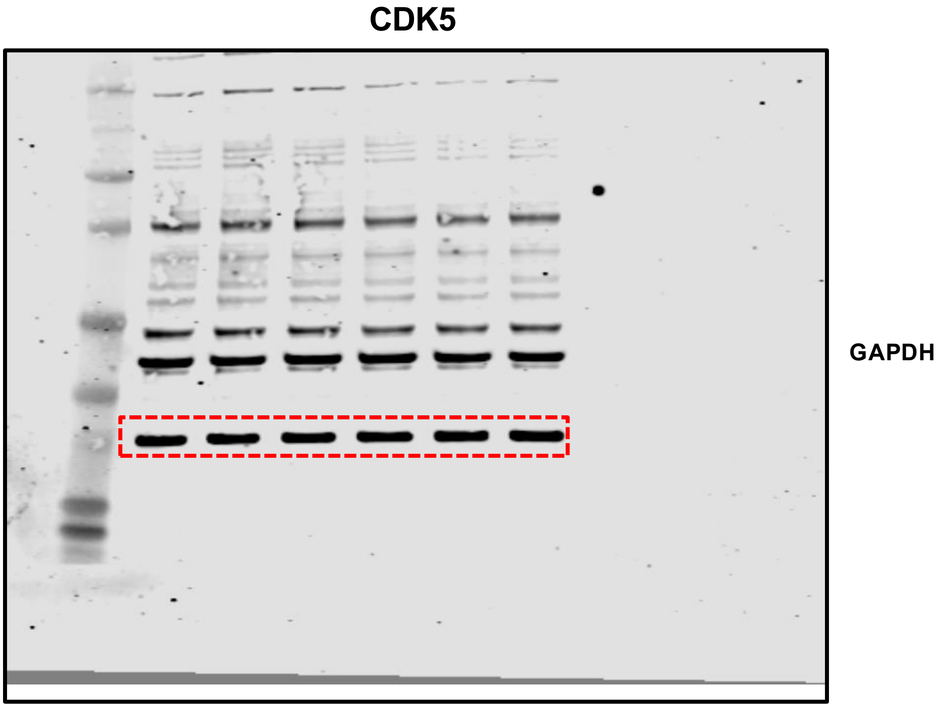

## Figure 5F

GAPDH

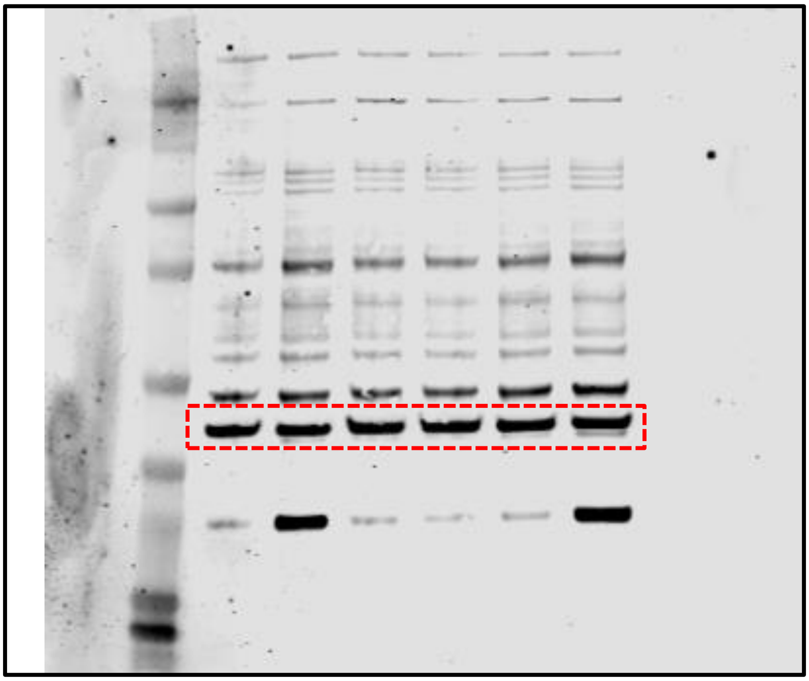

## Figure 6B

CDK5

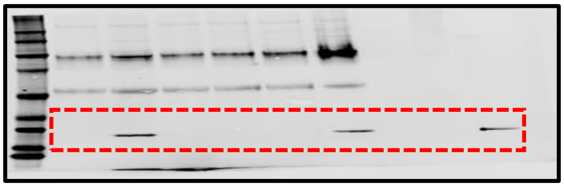

ITGA9

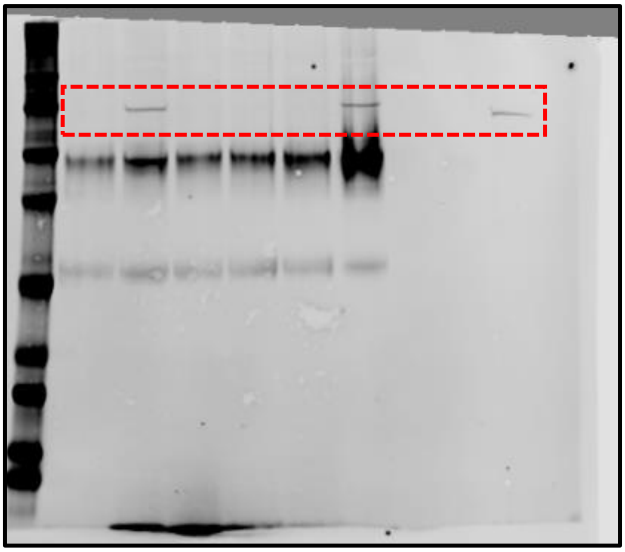

VEGFR2

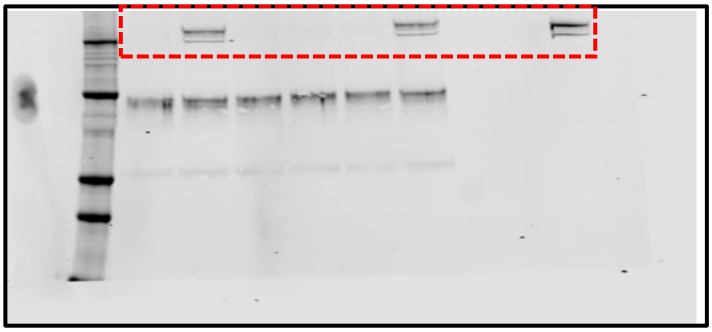

## Figure 6B

NRP1

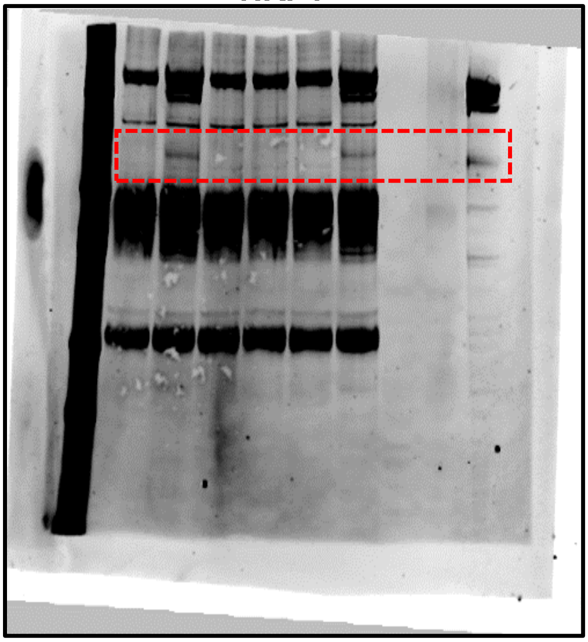

PLXND1

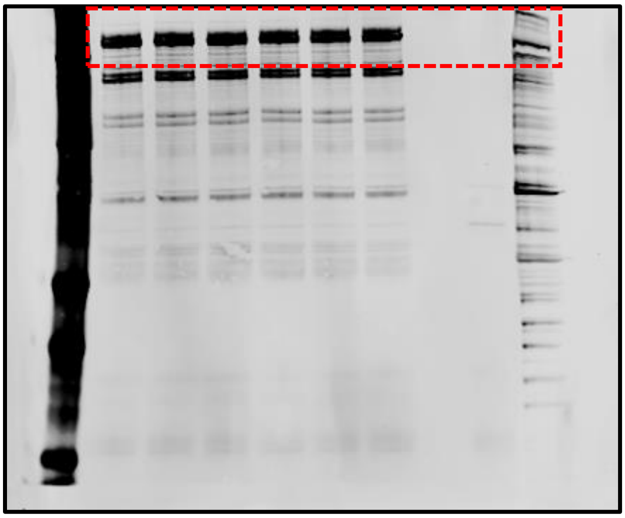

## Figure 6C

pSerine

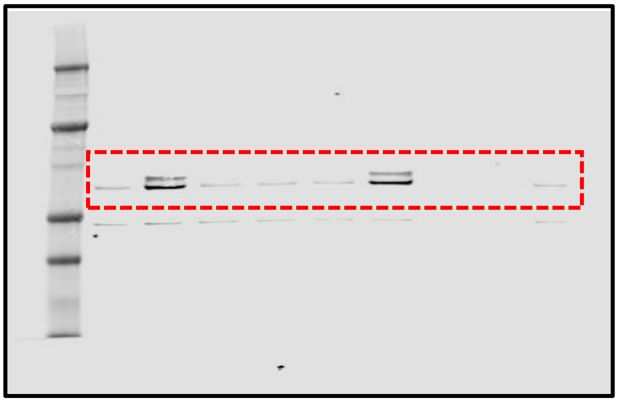

FOXC2

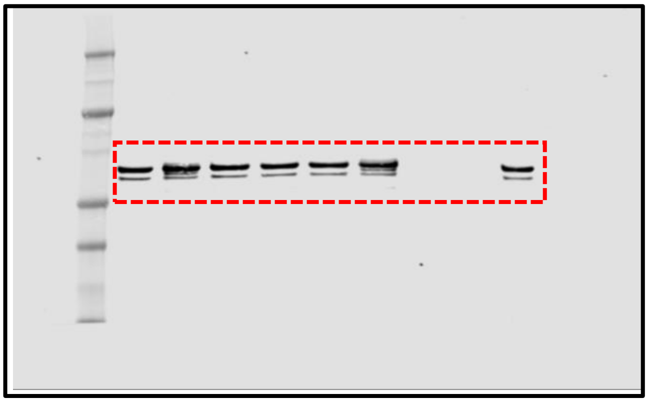

## Figure 6E

CDK5

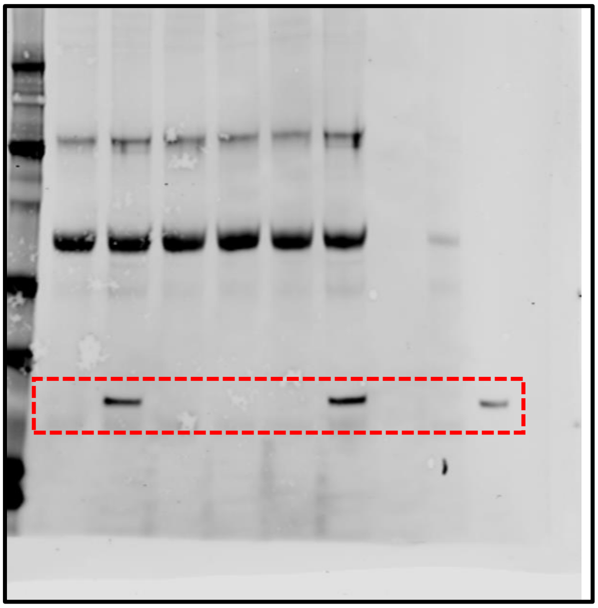

ITGA9

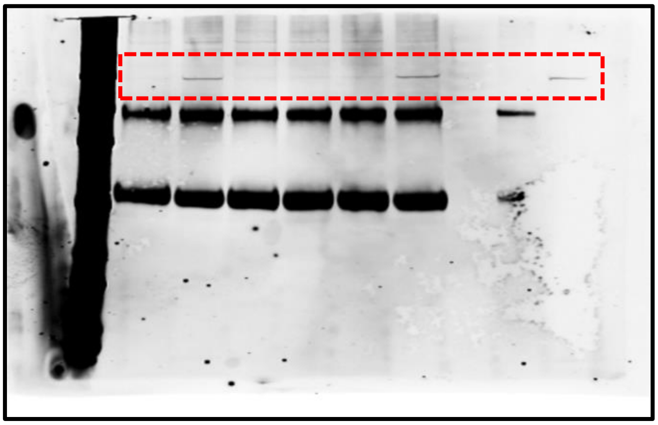

VEGFR2

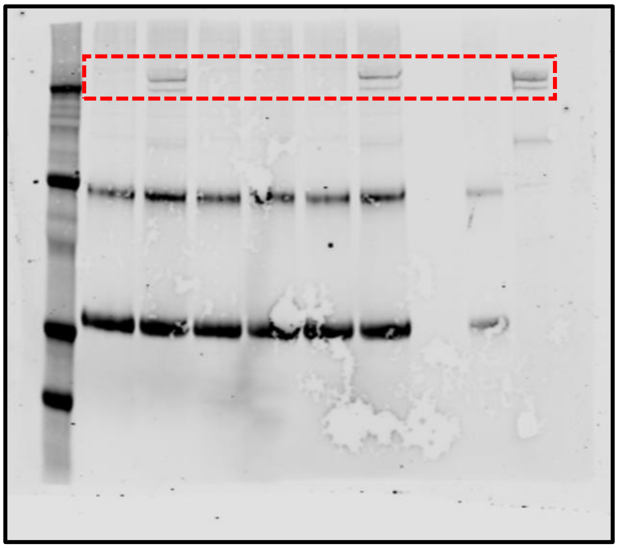

## Figure 6E

NRP1

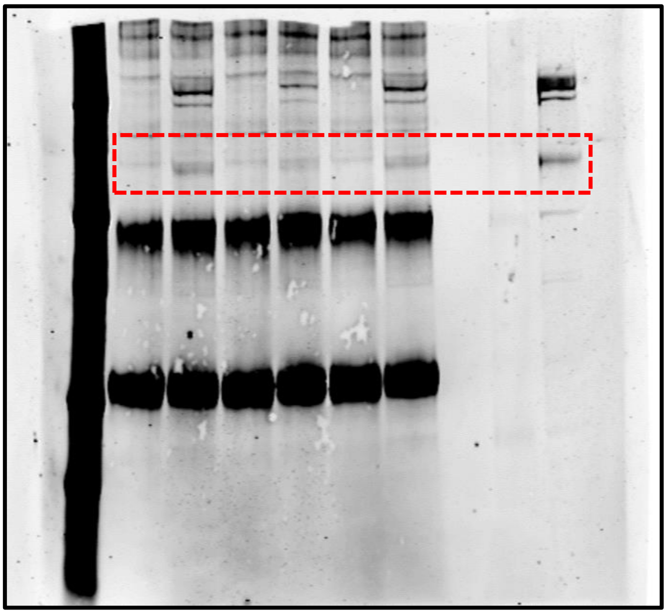

PLXND1

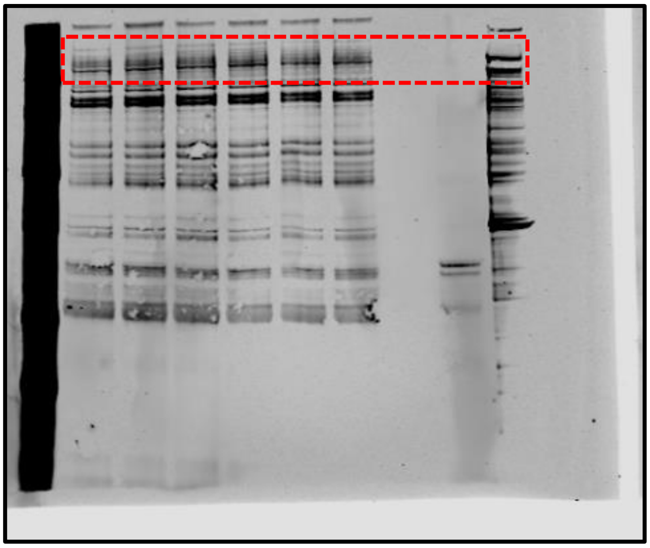

## Figure 6F

pSerine

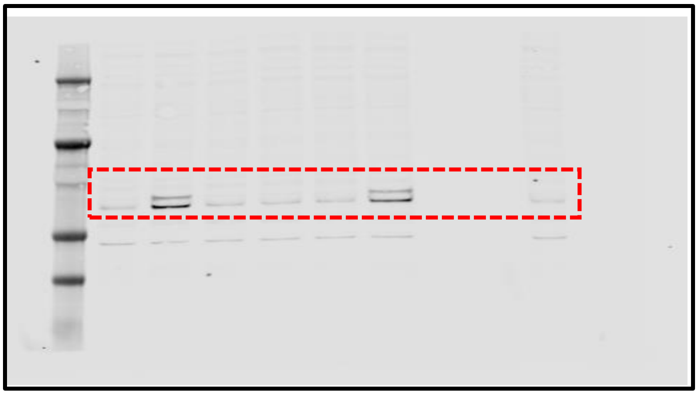

FOXC2

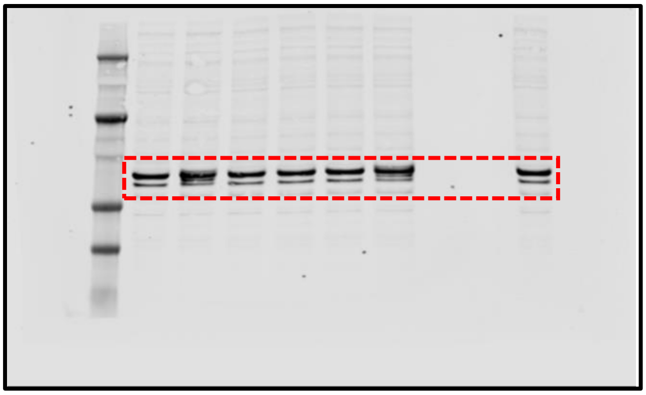

## Figure S9

PLXND1

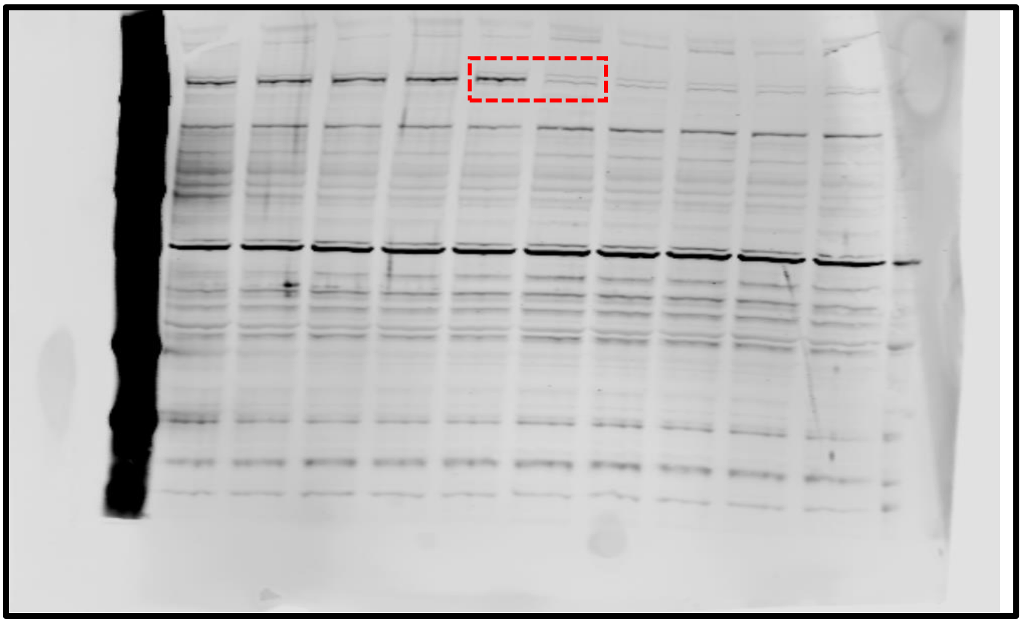

GAPDH

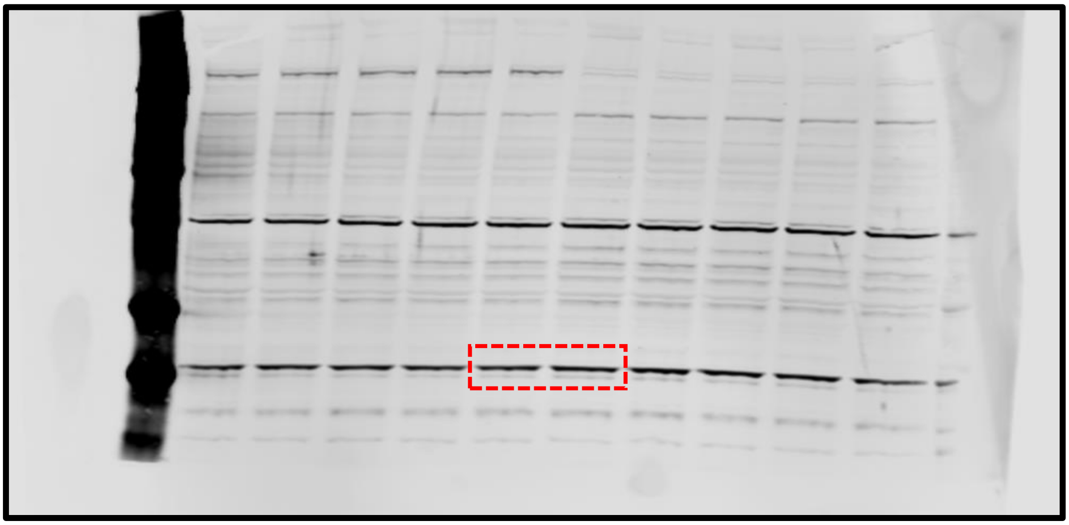

## Figure S12

CDK5

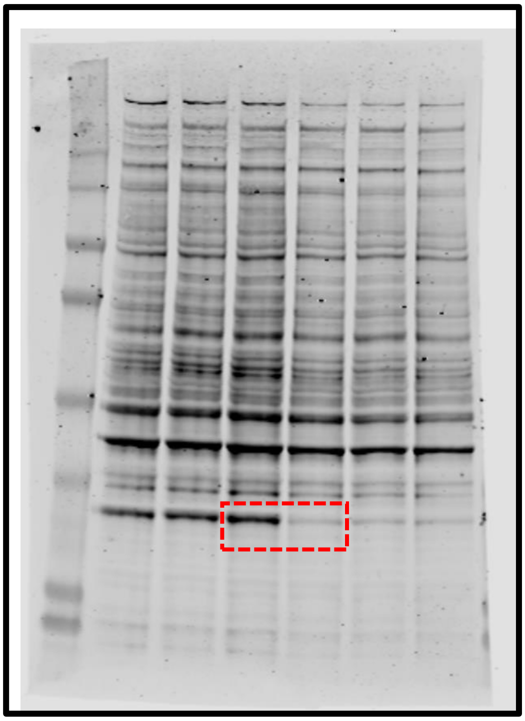

GAPDH

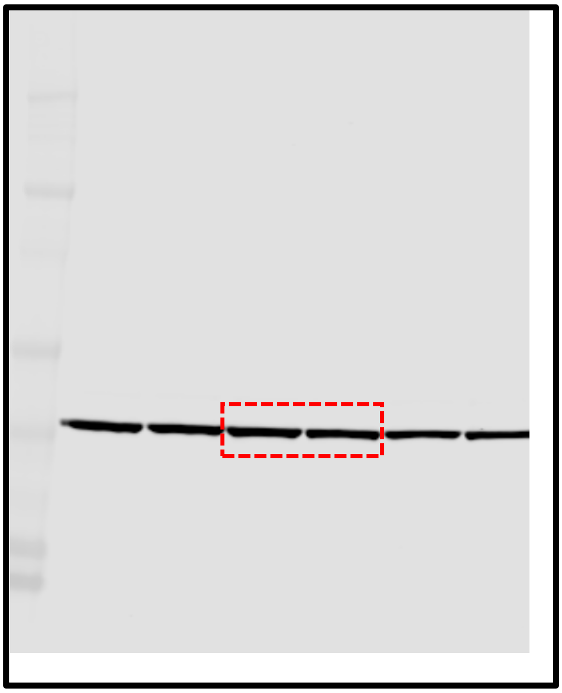

# Figure S14

VEGFR2

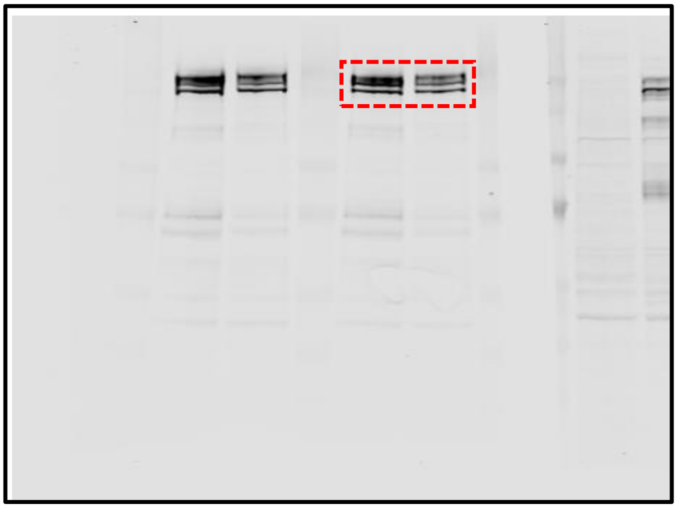

GAPDH

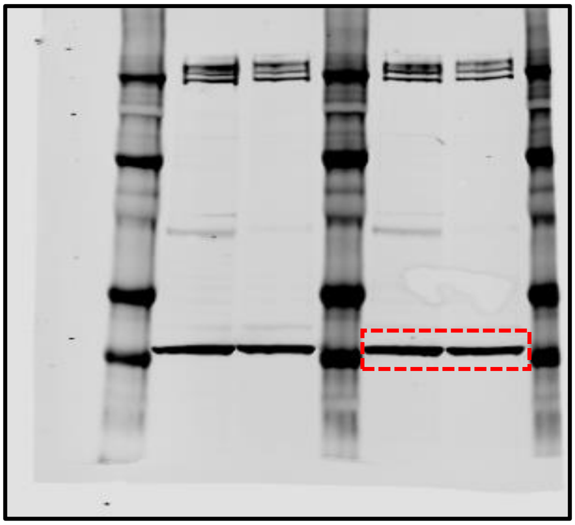

NRP1

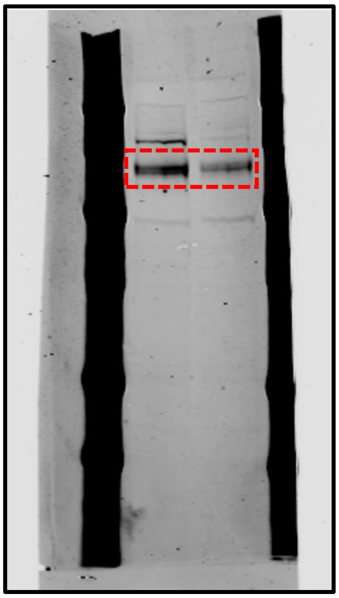

GAPDH

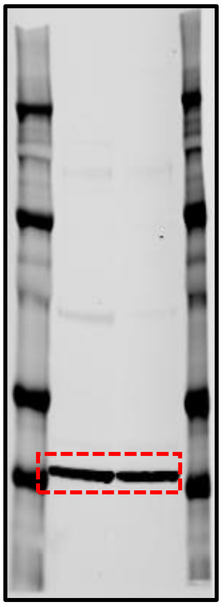

VEGFR3

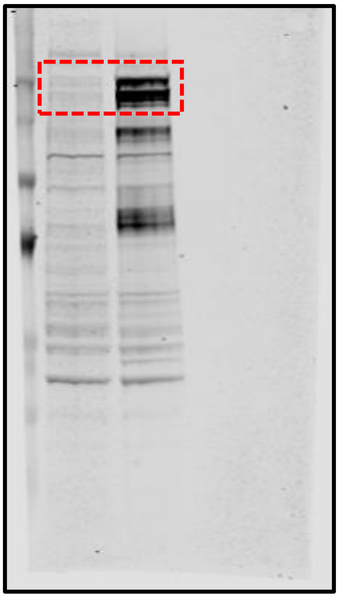

GAPDH

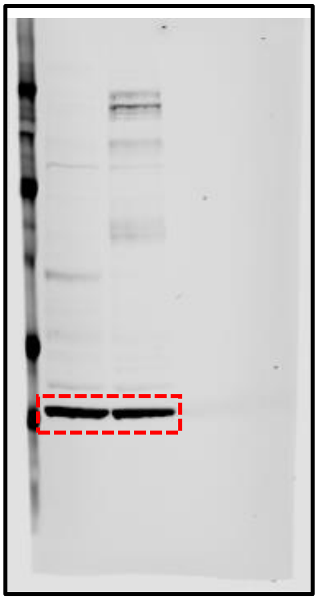

# Figure S14

NRP2

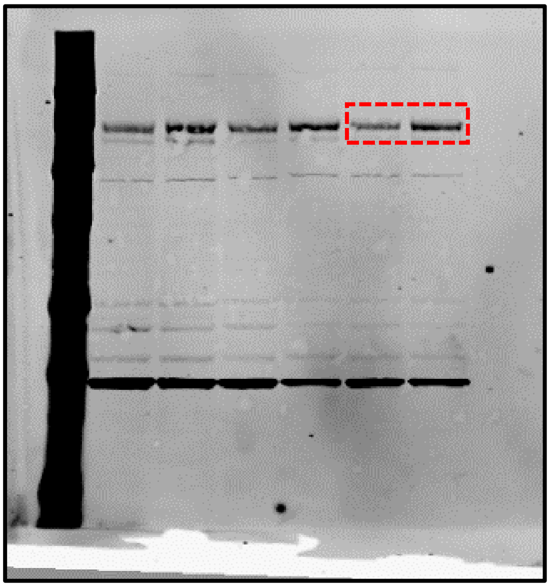

GAPDH

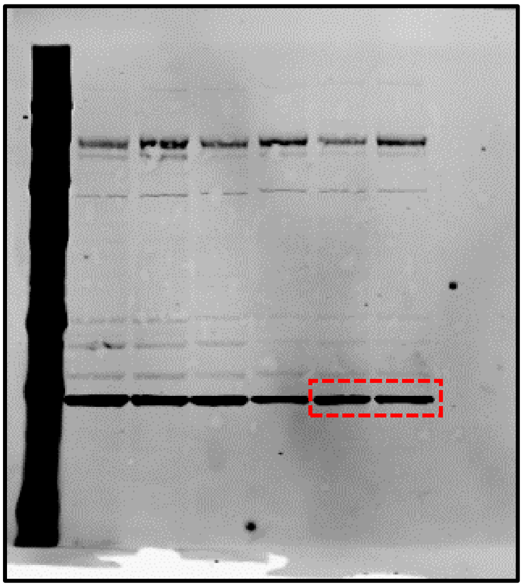

ITGA9

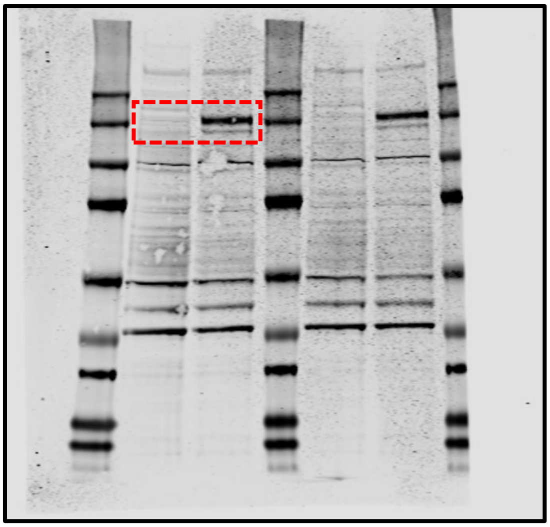

GAPDH

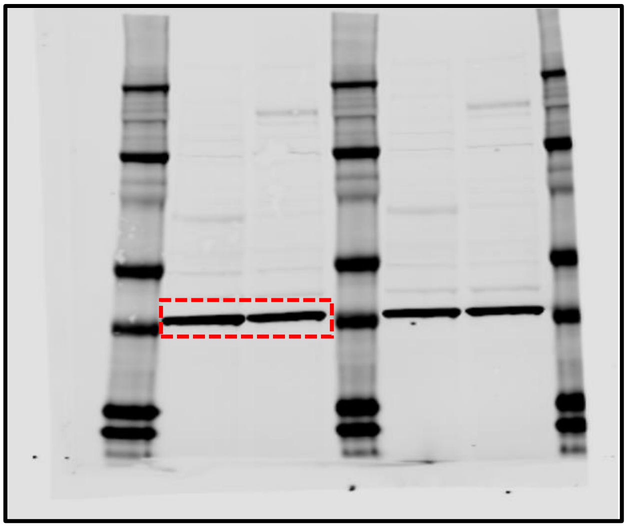

## Figure S15

NRP2

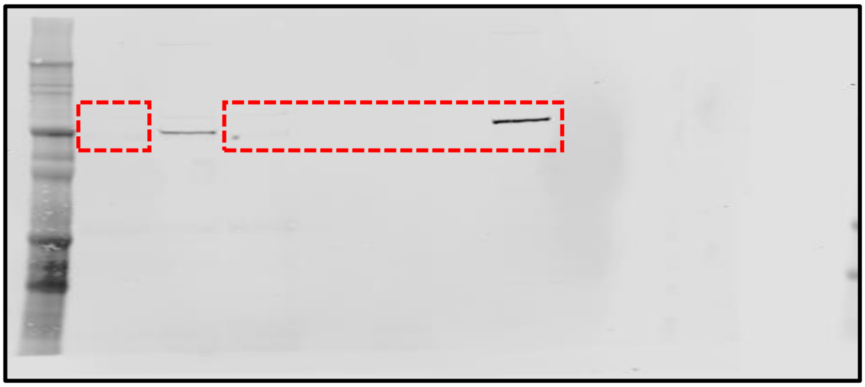

VEGFR3

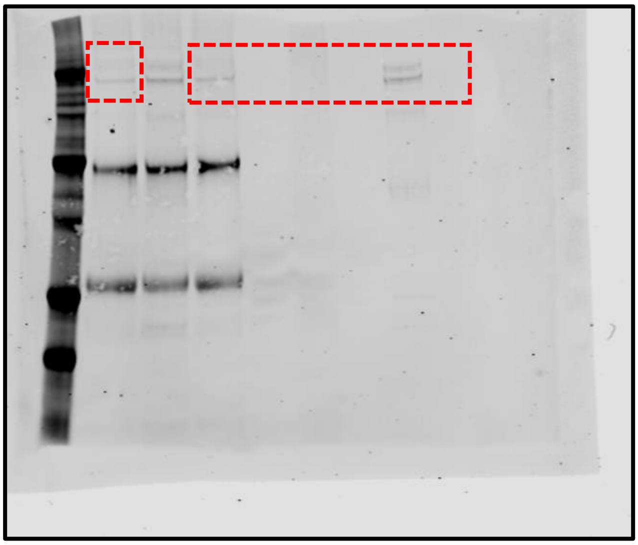

PLXND1

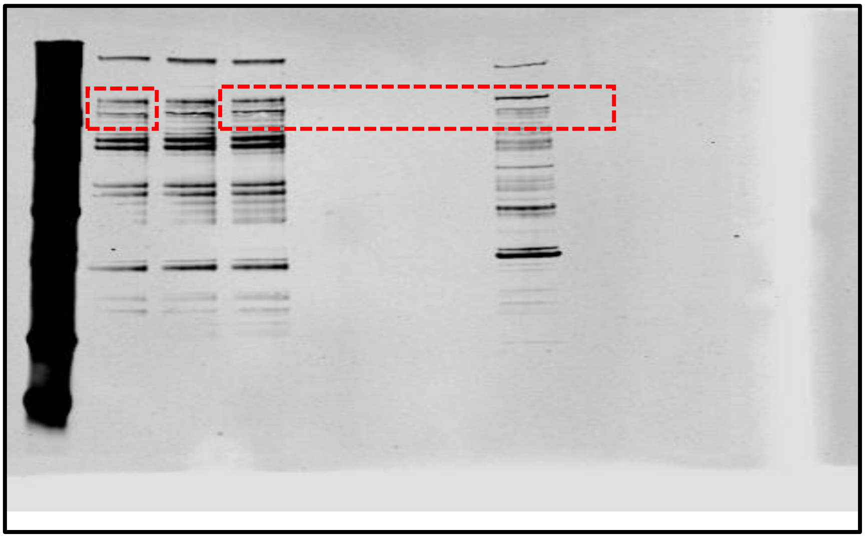

**Figure S16**

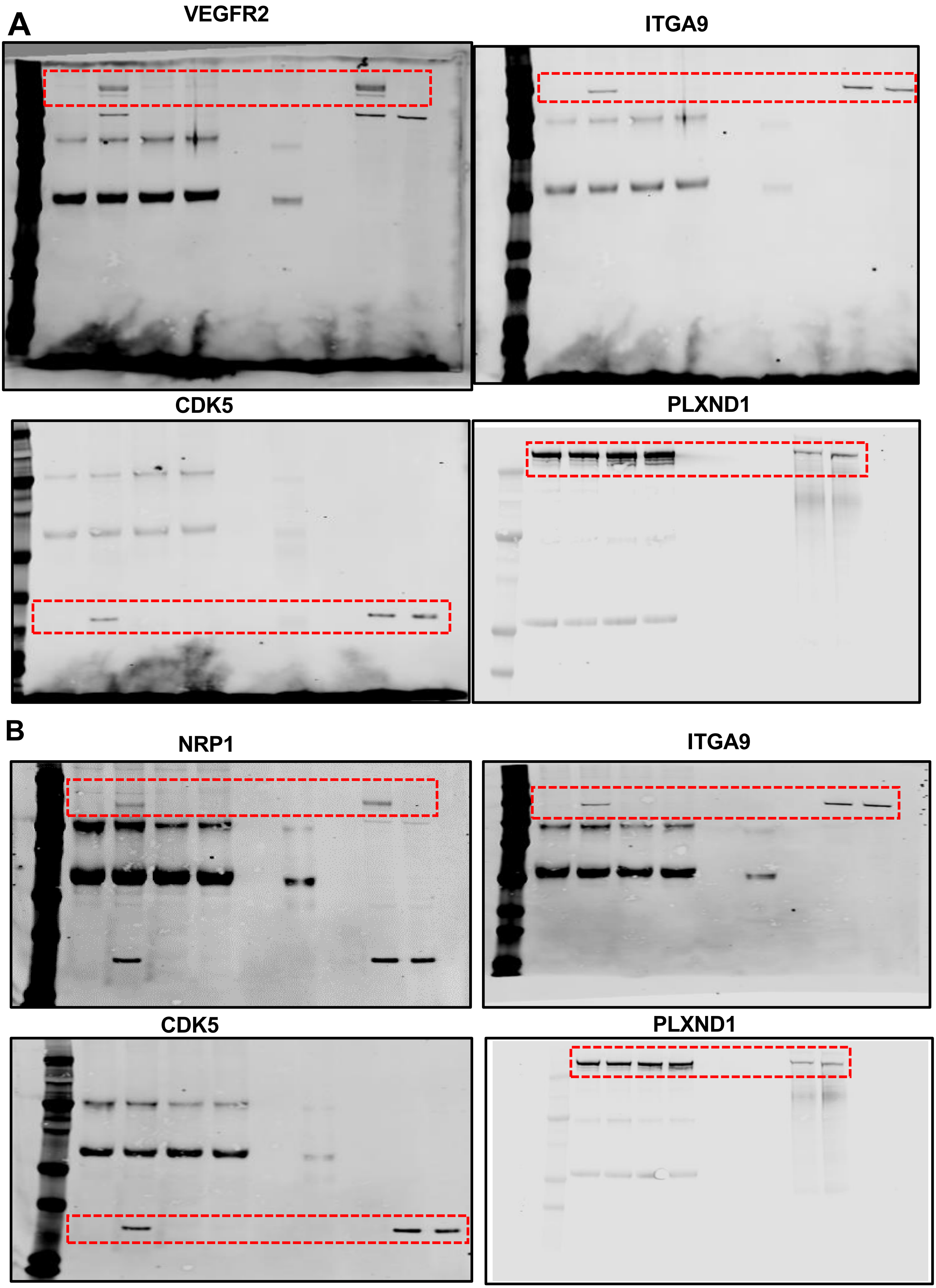

## Figure S17

CDK5

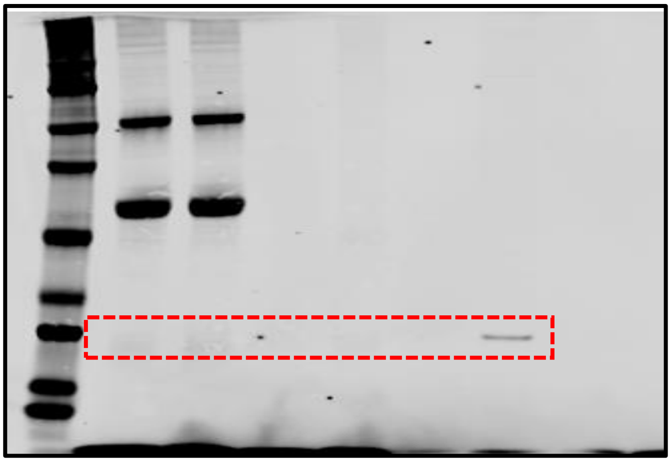

ITGA9

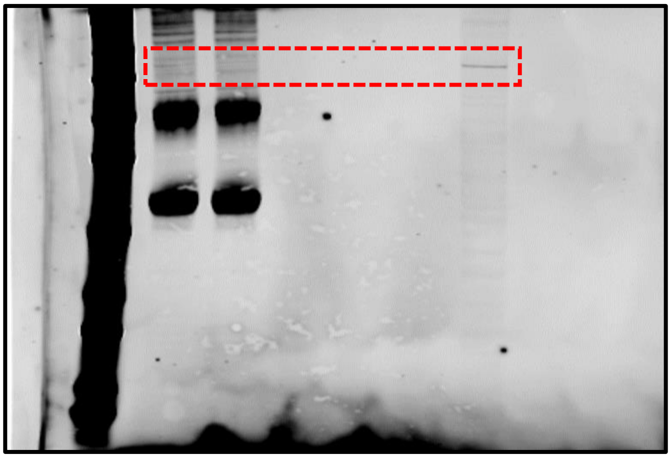

VEGFR2

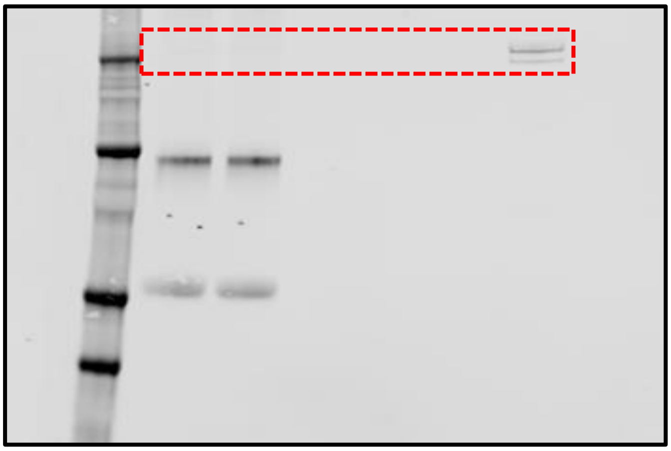

NRP1

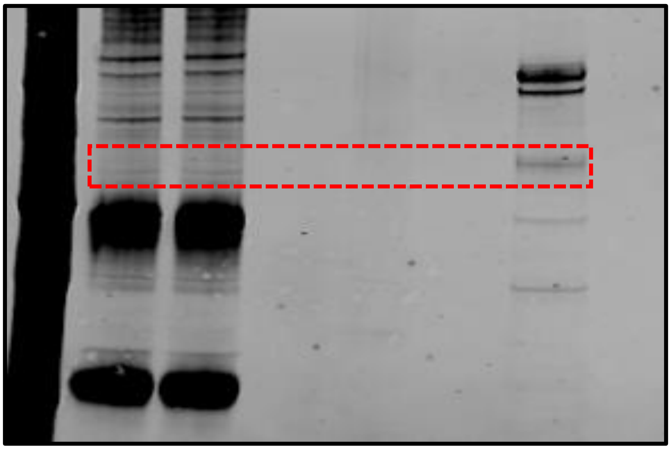

PLXND1

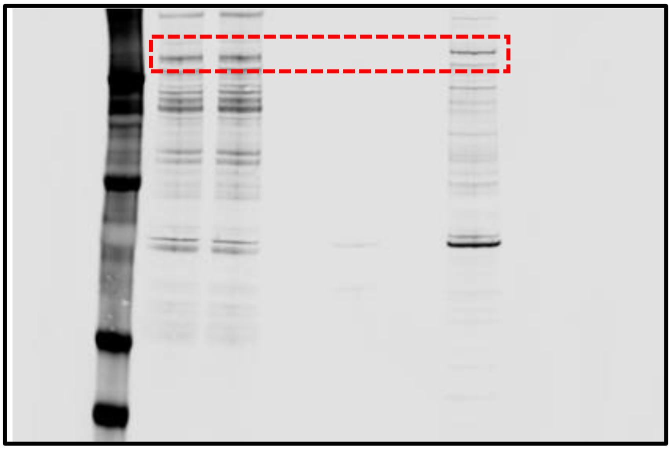

# Figure S19

PLXND1

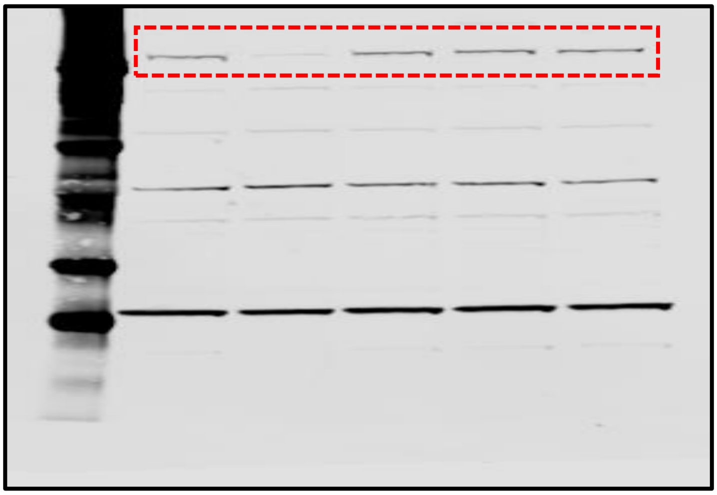

GAPDH

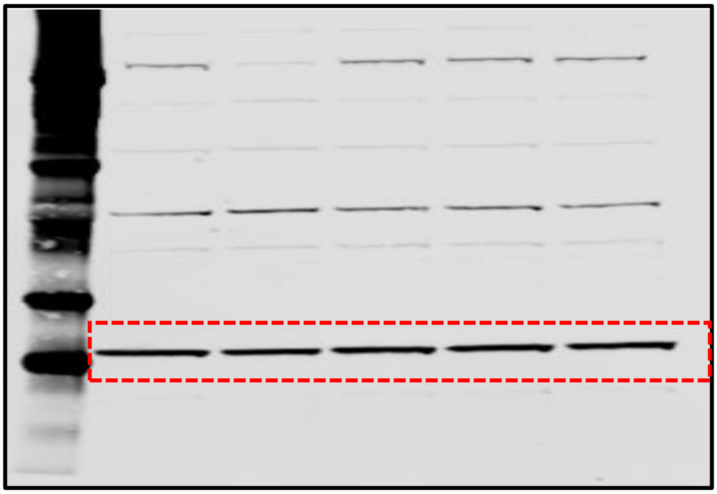

## Figure S21

NRP1

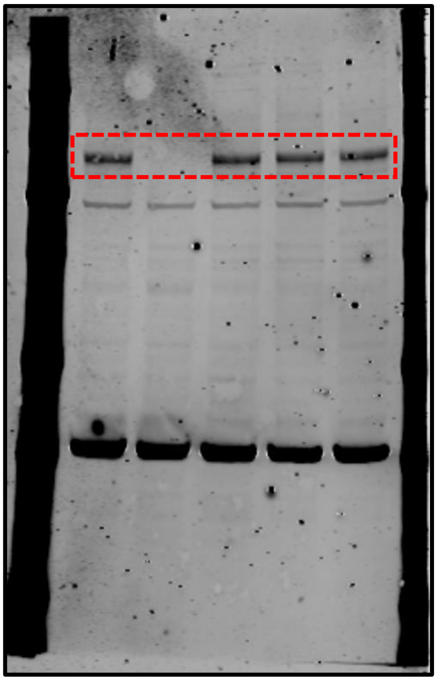

GAPDH

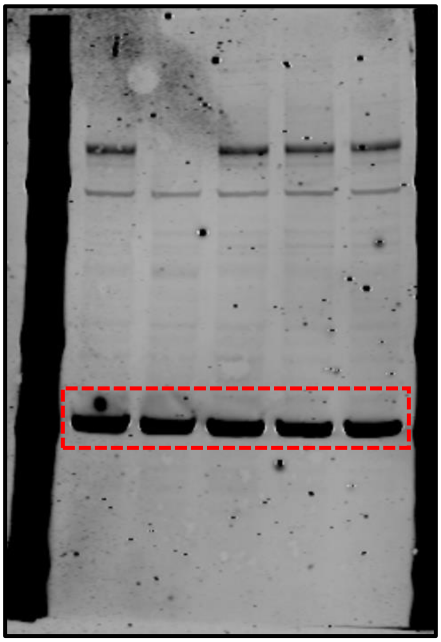

Supplement: Unedited blot and gel images [file jci-136-193385-s021.pdf]
